# Catheter Injectable Multifunctional Biomaterial for the Treatment of Infected Enterocutaneous Fistulas

**DOI:** 10.1002/advs.202414642

**Published:** 2025-02-14

**Authors:** Jinjoo Kim, Zefu Zhang, Hassan Albadawi, Hyeongseop Keum, Joseph L. Mayer, Erin H. Graf, Rahmi Oklu

**Affiliations:** ^1^ The Laboratory for Patient‐Inspired Engineering Mayo Clinic 13400 East Shea Blvd. Scottsdale AZ 85259 USA; ^2^ Department of Laboratory Medicine and Pathology Mayo Clinic 5777 E Mayo Blvd Phoenix AZ 85054 USA; ^3^ Division of Vascular & Interventional Radiology Mayo Clinic 5777 E Mayo Blvd Phoenix AZ 85054 USA

**Keywords:** anti‐bacterial, biomaterial, enterocutaneous fistula, hydrogel, sepsis

## Abstract

Enterocutaneous fistulas (ECF) are challenging to treat contributing to high morbidity and high mortality rates, significantly impacting the quality of life of the patients. Its susceptibility to antibiotic‐resistant infections often leads to chronic inflammation, complicating treatment with conventional methods. Here, 18NC75‐10P‐1IL is reported, which is a multi‐functional shear‐thinning hydrogel comprised of gelatin and nanosilicates for injectability, an ionic liquid for bactericidal effects, and platelet rich fibrin fraction for pro‐healing properties; this biomaterial is engineered for the treatment of ECFs. Through rigorous testing, the mechanical properties of 18NC75‐10P‐1IL were tailored for catheter injection to achieve durable occlusion of fistulous tracts under external pressures simulating clinical scenarios. 18NC75‐10P‐1IL demonstrated pro‐healing effects and anti‐microbial activity against highly resistant patient‐derived bacteria known to be associated with ECF. Subcutaneous implantation and anorectal fistula models confirmed its biocompatibility, pro‐healing, anti‐inflammatory, and anti‐microbial properties compared to control materials, suggesting promising potential for clinical translation in the treatment of human ECFs.

## Graphical abstract

A multi‐functional shear‐thinning hydrogel is engineered to treat enterocutaneous fistulas. This hydrogel can resist external pressures allowing it to remain within the fistula tract and is shown to have significant antimicrobial, pro‐healing, and anti‐inflammatory properties, which were validated in in vitro and in vivo experiments.

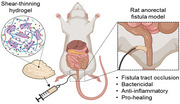

## 1 Introduction

ECF is an abnormal connection between the bowel and the skin. It can be associated with discharge of pus, feces, and stomach contents and can even lead to incontinence.^[^ ^1^ ^]^ ECFs are referred to as surgical tragedies in the literature; up to 85% are the result of intra‐abdominal surgical complications, such as missed enterotomies or anastomotic leaks.^[^ ^2^ ^]^ They can also result from inflammatory bowel disease, diverticulitis, radiotherapy, trauma, or malignancy.^[^ ^2^, ^3^ ^]^ Patients with Crohn's disease are particularly affected; ≈40% (280000) develop a fistula.^[^ ^4^ ^]^ Although mortality rates have improved over the last several decades, modern series still report overall ECF mortality of 15–20% secondary to sepsis from frequent infectious complications, nutritional deficiencies leading to severe malnutrition, and electrolyte disturbances.^[^ ^5^ ^]^ The main ECFs involve small bowel (63%) and colon (26%),^[^ ^5b^ ^]^ where wide neck, deep fistulous cavities are typically non‐healing. Only 20–30% of ECFs close spontaneously, while the remaining requires intervention.^[^ ^1^, ^5^ ^]^ Despite advances in surgical and interventional radiology techniques, no successful treatment for ECF exists. Patients are often subject to repeat interventions with postoperative morbidity of up to 86%; frequent follow‐up imaging with X‐ray fluoroscopy and computed tomography (CT) imaging are also common, exposing the patient to excess radiation. ECF often results in prolonged intensive care unit stays increasing health care costs which can exceed $500000 per visit.^[^ ^6^ ^]^

ECFs can lead to constant leakage of enteric and fecal contents from the skin, sometimes up to many liters per day. The foul enteric contents act as a chemical irritant to the fistula tract, causing chronic damage and elevating susceptibility to infections.^[^ ^2^, ^3^, ^5^ ^]^ Infections associated with ECF typically involve bacteria originating from the gut or the skin, including *Escherichia coli* (*E. coli*), *Klebsiella pneumoniae* (*K. pneumoniae*), and *Enterococcus spp*., *Pseudomonas aeruginosa* (*P. aeruginosa*), and *Staphylococcus spp*.^[^ ^7^ ^]^ More importantly, it was recently reported that 96.3% of gram‐negative isolates from ECF exhibited multi‐drug resistance (MDR), underscoring the difficulty in medically addressing infected ECFs.^[^ ^8^ ^]^

Treatment of ECF involves controlling sepsis, providing fluid, electrolyte, and nutrition replacement, protecting the skin from enteric effluent, defining anatomy, and planning a definitive procedure.^[^ ^9^ ^]^ Medical management to reduce fistula output can be attempted with medications, such as antidiarrheals or somatostatin (a natural anti‐secretory hormone), and dressings, such as ostomy appliances or negative pressure wound therapy. Nonoperative therapies, including fibrin glue, endoscopic clips, and fistula plugs, have also been investigated in the treatment of ECF, however, they have low efficacy. Fibrin glue has demonstrated some success in decreasing time to fistula closure. However, these data are based on small case reports.^[^ ^10^ ^]^ Fibrin glue often fails due to its liquid consistency, causing it to leak out after the patient becomes mobile; in one study, the failure rate was reported to be 85%.^[^ ^11^ ^]^ Endoscopic clip closure has shown increased interest in enteric fistula treatment with long‐term success rates of 40%. However, it is not suited for chronic fistulas and for those not accessible through endoscopy.^[^ ^12^ ^]^ Fistula plugs, such as the Biodesign Fistula Plug (Cook Medical, Indiana, USA) have also been trialed for ECFs with some initial success, however, data is based on small case series and outcomes are based on short‐term results.^[^ ^10a^ ^]^ These treatments fail due to a number of factors, most commonly due to dislodgement of the material and to recurring sepsis. Plugs are unsuitable for complex fistulas with multiple branches, blind endings, and secondary openings. For some, the best treatment option is definitive surgery, which results in healing rates of 82–86%. However, recurrences are reported at 21–32% with postoperative morbidity of 86%.^[^ ^1^, ^2^, ^13^ ^]^ Additionally, not all patients are operative candidates as surgical approaches are also options depending on the fistula anatomy and the ability of the patient to tolerate the procedure. The prevalence, complexity, and current inability to treat ECFs effectively demand the development of a new approach, a new direction for our patients. A creative bioengineered biomaterial that can be delivered through image guidance using minimally invasive techniques may offer control over bacterial infection and provide time for a sustained sterile occlusion of the fistula tract to promote the healing process.

In recent decades, injectable hydrogels have emerged as promising materials in a range of biomedical applications such as drug delivery and tissue engineering for their exceptional mechanical properties, capacity to encapsulate and release diverse bioactive agents, and potential for administration into challenging or inaccessible areas using minimally invasive techniques.^[^ ^14^ ^]^ Shear‐thinning hydrogel is one type of these injectable hydrogels, which exhibits fluid‐like behavior under shear stress (i.e., shear‐thinning) and regains its mechanical properties (i.e., self‐healing) once the stress is removed.^[^ ^15^ ^]^ These unique properties confer several advantages, including uniform encapsulation of cargo, facile injection via needles or catheters, and sustained retention at the injection site,^[^ ^16^ ^]^ thereby enhancing drug delivery efficiency while minimizing systemic side effects.^[^ ^15b^ ^]^

Platelet‐rich fibrin (PRF), an autologous concentrate of platelets and growth factors obtained through the centrifugation of a patient's own blood, has emerged as a promising treatment for wound healing due to its therapeutic efficacy, simplified preparation,^[^ ^17^ ^]^ safety, and cost‐effectiveness.^[^ ^18^ ^]^ The therapeutic efficacy of PRF has been primarily attributed to the roles of growth factors (e.g., PDGF, TGF‐β, VEGF, and bFGF) in cell proliferation, migration, extracellular matrix synthesis, and angiogenesis. Recently, the anti‐inflammatory activity of PRF has also been acknowledged as a significant contributor to its therapeutic efficacy.^[^ ^19^ ^]^ Its ability to expedite healing process has been investigated across in vitro,^[^ ^20^ ^]^ in vivo,^[^ ^21^ ^]^ and various clinical situations such as hard‐to‐treat skin ulcers,^[^ ^20^, ^22^ ^]^ periodontal tissue defects, and urethracutaneous fistula.^[^ ^23^ ^]^ To broaden the applicability of PRF, Izzet et al. conducted a study revealing that the lyophilization of PRF does not compromise the function of bioactive factors or impede its clinical effects packaged in an injectable biomaterial in a vascular embolization model.^[^ ^24^ ^]^

**Figure 1:**
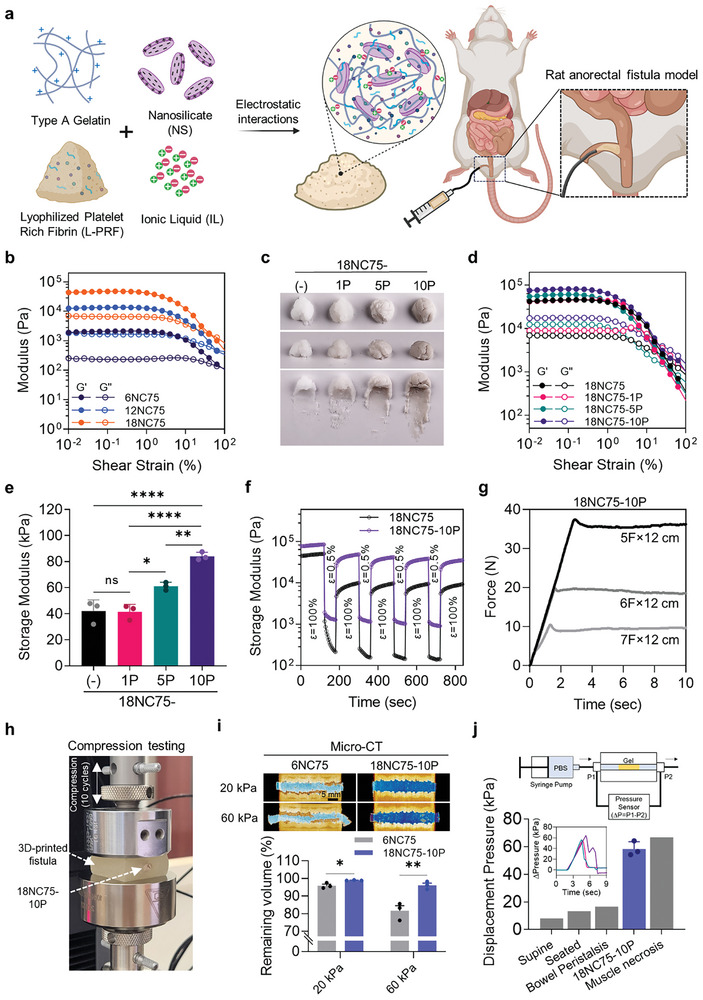
Mechanical optimization and characterization of 18NC75‐10P. a) Schematic illustrating the preparation of the multifunctional 18NC75‐10P‐1IL hydrogel for the occlusion of ECF tracts featuring antimicrobial, pro‐healing, and anti‐inflammatory properties. b) Representative flow curves of oscillatory strain sweeps for 6NC75, 12NC75, and 18NC75 at a constant angular frequency of 10 rad s^−1^. Shear strain ranges from 10^−2^ to 10^2^%. c) Image of 18NC75 with varying L‐PRF concentrations (0, 1, 5, and 10 w/w%), demonstrating visual material differences. d) Representative flow curves of oscillatory strain sweeps for 18NC75, 18NC75‐1P, 18NC75‐5P, and 18NC75‐10P at a constant angular frequency of 10 rad s^−1^. Shear strain ranges from 10^−2^ to 10^2^%. e) Graph depicting the storage modulus generated by 18NC75, 18NC75‐1P, 18NC75‐5P, and 18NC75‐10P at a shear strain of 10^−1^% (*n* = 3). f) Thixotropy test measuring the storage modulus of 18NC75 and 18NC75‐10P under alternating 100% high strain for 2 min and 0.5% low strain for 1 min at a constant angular frequency of 10 rad s^−1^ demonstrating recoverability. **g**, Representative time‐dependent injection force flow curves displaying the compression force generated after injecting 18NC75‐10P through catheters of various sizes. h) Photograph of the compression testing setup used to apply 10 cycles of compression forces to the fistula‐mimicking 3D‐printed model filled with 18NC75‐10P hydrogel. i) Reconstructed micro‐CT images illustrating fistula models filled with either 6NC75 or 18NC75‐10P hydrogel before and after applying 10 cycles of compression pressure ranging between 0 – 20 kPa or 0 – 60 kPa. The graph shows the percentage of remaining volume inside the fistula model after cyclic compression (*n* = 3). j) Illustration of the experimental setup for measuring displacement pressure, accompanied by flow curves and a quantitative analysis highlighting the maximum pressure needed to displace 18NC75‐10P from a 3D‐printed model mimicking a fistula (*n* = 3). Data are presented as mean ± s.e.m. Statistical significance was determined by one‐way ANOVA with Tukey's post‐hoc tests in e or unpaired Student's t‐test in i. ns, not significant, *p < 0.05, **p < 0.01, ***p < 0.001, ****p < 0.0001.

Here, we developed a shear‐thinning biomaterial for the effective treatment of ECF capable of durably occluding fistula tracts and exhibiting pro‐healing, antimicrobial, and anti‐inflammatory properties (**Figure** **1**a). The basic composition includes porcine gelatin (type A) and nanosilicate (NS; Laponite XLG) at an optimized ratio to create a shear‐thinning nanocomposite hydrogel with desired mechanical properties, including high mechanical strength, recoverability, injectability, and displacement resistance under external pressure. To enhance the pro‐healing regenerative properties, L‐PRF was incorporated into the formulation, exhibiting cell proliferation, migration, and angiogenesis in vitro, and expediting healing of fistula tracts in a rat anorectal fistula model. For broad‐spectrum antimicrobial effects against numerous microorganisms, including MDR bacteria, we incorporated choline and geranate ionic liquid (IL) into the formulation.^[^ ^25^ ^]^ We validated the efficacy of the bactericidal effects in vitro against *E. coli* and various patient‐derived antibiotics‐resistant bacteria and in vivo in the rat model of anorectal fistula.

## 2 Results and Discussion

### 2.1 Optimization and Characterization of Gelatin + NS Nanocomposite Hydrogel

Porcine gelatin mixed with NS forms physical cross‐linking through strong electrostatic interactions between negatively charged surfaces of NS and positively charged gelatin chains, resulting in a shear‐thinning behavior.^[^ ^16^, ^26^, ^27^ ^]^ Since the mechanical property of this mixture can be tuned by altering the ratios of gelatin and NS, varying gelatin + NS formulations were tested for optimization of viscosity and modulus. Three different concentrations of total solids (NS + gelatin; x = 6, 12, and 18% w/w) comprising three different percentages of NS in the total solid mass (y = 25, 50, and 75% w/w) were used for testing, respectively. All nanocomposite (NC) formulations tested (i.e., xNCy) exhibited shear‐thinning properties, showing reduced viscosity as the shear rate increased, which is crucial for injectability (Figure S1a, Supporting Information). The maximum viscosity was found to increase with higher total solid concentration and NS ratio (Figure S1b, Supporting Information). Additionally, the storage modulus (G’) of xNCy increased consistently as x and y increased (Figure 1b; Figure S2a,b, Supporting Information), which correlated with the observed texture of the hydrogel (Figure S3, Supporting Information).

The SEM images analyzed for each NC formulation showed that the pore diameter and porosity tend to decrease with increasing total solid mass and NS ratio, suggesting an increase in cross‐linking density (Figure S4a–c, Supporting Information). These results correlate with the variation in the mechanical strength of different NCs, as demonstrated by the viscosity and storage modulus measurements. Notably, 18NC75 exhibited desirable mechanical properties (viscosity: 355.67 ± 45.39 kPa s^−1^ at maximum; storage modulus: 44.88 ± 4.26 kPa at a shear strain of 10^−1^%) among the tested formulations to minimize the likelihood of displacement within the ECF.

### 2.2 Incorporation of Various Concentrations of L‐PRF into 18NC75

To enable pro‐healing properties, L‐PRF was prepared from freshly collected porcine whole blood through centrifugation, followed by lyophilization (Figure S5a, Supporting Information).^[^ ^24^ ^]^ The weight yields of PRF and L‐PRF compared to whole blood were measured at 50.20 ± 0.87 and 4.12 ± 0.19%, respectively (Figure S5b, Supporting Information). In a cell proliferation assay using rat fibroblast cells (Rat2), L‐PRF exhibited a concentration‐dependent cell proliferative effect, suggesting that even after lyophilization, PRF retains its biological activity (Figure S6, Supporting Information). Next, we incorporated L‐PRF into the 18NC75 formulation at varying concentrations (1, 5, and 10% w/w), resulting in formulations termed 18NC75‐1P, 18NC75‐5P, and 18NC75‐10P (Figure 1c). The modulus of these formulations increased with higher L‐PRF content (Figure 1d,e). However, the viscosity remained constant regardless of the added L‐PRF concentration (Figure S7a,b, Supporting Information). The addition of L‐PRF to 18NC75 formulation led to an increase in the material's zeta potential (Figure S8, Supporting Information). This phenomenon is likely attributed to electrostatic interactions between NS and L‐PRF, which offset the negative charges of NS. After the addition of L‐PRF to 18NC75, the microporous structures were rarely observed in 18NC75‐10P, suggesting that L‐PRF was incorporated into the porous structures (Figure S9, Supporting Information). Based on its superior mechanical strength and the highest L‐PRF incorporation, 18NC75‐10P was selected for further characterization and optimization.

### 2.3 Assessment of Mechanical Properties of 18NC75‐10P

18NC75‐10P was further tested for recoverability, injectability, and resistance to displacement against external pressures such as perpendicular pressure (i.e., sitting) or axial pressure (i.e., peristalsis). The results demonstrated that 18NC75‐10P exhibited recoverability under oscillating strains (Figure 1f) and injectability through catheters of various sizes (5, 6, and 7F × 12 cm length) (Figure 1g; Figure S10, Supporting Information). To assess the mechanical suitability of 18NC75‐10P for occluding ECF, its resistance to displacement was evaluated under simulated perpendicular or axial pressure applied to a 3D‐printed model mimicking a fistulous tract surrounded by soft tissue. The Young's modulus of the 3D‐printed fistula model (Figure S11, Supporting Information) was 217.69 ± 2.88 kPa, which closely matched that of the measured rat dorsal tissue (201.56 ± 15.48 kPa), indicating that the 3D‐printed fistula model mimics the mechanical strength of mammalian soft tissue (Figure S12a–c, Supporting Information). To assess displacement against perpendicular pressure (Figure 1h), the 3D‐printed fistula model filled with 18NC75‐10P was repeatedly applied 10 times at 20 or 60 kPa using a mechanical tester (Figure S13, Supporting Information). 6NC75 was chosen as the control based on its modulus value, as shown in Figure 1b. Post‐compression testing, the material volume remaining within the tract measured using micro‐CT image analysis revealed that 18NC75‐10P experienced minimal displacement from the tract at both 20 and 60 kPa (1.10 ± 0.12% and 3.92 ± 1.25%, respectively). In contrast, the control samples exhibited significantly higher volume loss (4.10 ± 0.88% and 18.29 ± 2.84% at 20 and 60 kPa, respectively) (Figure 1i). Next, we assessed the axial pressure required to displace the material, as depicted in Figure S14a (Supporting Information). The axial pressure required to displace 18NC75‐10P loaded inside the tract of the 3D‐printed fistula model was 58.22 ± 3.05 kPa, which was higher than that of 6NC75 (28.64 ± 7.12) (Figure S14b,c, Supporting Information). Notably, the required displacement pressure exceeds the estimated pressure exerted by the human bowel peristaltic movement (5 – 16.6 kPa)^[^ ^28^ ^]^ and approaches the non‐physiologic pressures that can lead to muscle necrosis in pigs (66.7 kPa) (Figure 1j).^[^ ^29^ ^]^ These findings imply that 18NC75‐10P demonstrates desirable mechanical characteristics that can potentially occlude fistulous tracts in vivo.

### 2.4 Assessment of Biological Functionality of 18NC75‐10P In Vitro

**Figure 2:**
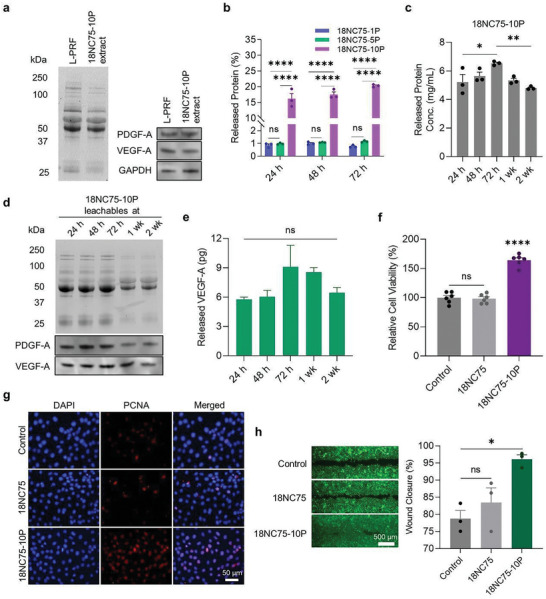
Evaluating the biological functions of 18NC75‐10P. a) SDS‐PAGE blot image compares protein fractions in L‐PRF and 18NC75‐10P extract. The Western blot images depict specific protein bands for PDGF‐A, VEGF‐A, and GAPDH. b) Graph illustrates the percentage of released proteins from 18NC75‐1P, 18NC75‐5P, and 18NC75‐10P, relative to the initially loaded amount, at 24, 48, and 72 h post‐incubation at 37 °C in DPBS (*n* = 3). c) Graph displays the total released protein concentrations from 18NC75‐10P incubated in DPBS at 37 °C, measured up to 2 weeks (*n* = 3). d) SDS‐PAGE and Western blot images depict PDGF‐A and VEGF‐A in the 18NC75‐10P leachables up to 2 weeks after incubation. e) Quantitative analysis of the time‐dependent release of VEGF‐A from 18NC75‐10P (*n* = 3). f) Plot illustrates the relative cell viability of Rat2 cells after incubation with 18NC75‐10P leachables (*n* = 6). g) Representative PCNA^+^ immunofluorescent images of Rat2 cells, comparing untreated control with cells treated with 18NC75 or 18NC75‐10P leachables. h) Fluorescence image and graph demonstrating enhanced migration of Rat2 cells at 24 h after treatment with leachables from 18NC75 or 18NC75‐10P compared to control. Data are presented as mean ± s.e.m. Statistical significance was determined by one‐way ANOVA (c, e, f, and h) or two‐way ANOVA (b) with Tukey's multiple‐comparison test. ns, not significant; *p < 0.05, **p < 0.01, ***p < 0.001, ****p < 0.0001.

To assess the preservation of protein integrity of L‐PRF after the incorporation into 18NC75 formulation, extracts from native L‐PRF and 18NC75‐10P were analyzed using SDS‐PAGE gel electrophoresis and Western blots to detect the most prominent growth factors involved in tissue healing, PDGF‐A and VEGF‐A (**Figure** **2**a). The protein fractions in both L‐PRF and 18NC75‐10P extracts were similar, with no notable signs of protein degradation. The presence of comparable PDGF‐A, VEGF‐A, and GAPDH protein bands in both samples suggests that proteins derived from L‐PRF maintained their integrity post‐incorporation into 18NC75. To assess the impact of L‐PRF concentration within 18NC75 on protein release, the percentage of total released protein was measured for 18NC75‐1P, 18NC75‐5P, and 18NC75‐10P at 24, 48, and 72 h after incubation with DPBS at 37 °C (Figure 2b). While the protein release for 18NC75‐1P and 18NC75‐5P remained near 1% over 72 h, 18NC75‐10P demonstrated a higher protein release (19.65 ± 0.36% at 72 h). This is likely attributed to the saturation of electrostatic interactions between L‐PRF and NS as the concentration of L‐PRF increased, which is also supported by the zeta potential measurements (Figure S8, Supporting Information). The concentration of released proteins showed slight fluctuations over 2 weeks but generally remained constant, suggesting sustained stabilization and release of the incorporated proteins (Figure 2c). To evaluate the time‐dependent integrity preservation of proteins released from 18NC75‐10P in DPBS at 37 °C, leachables collected at different timepoints (24 h, 48 h, 72 h, 1 wk, and 2 wk) were analyzed by Western blot targeting PDGF‐A and VEGF‐A (Figure 2d). Reductions in band intensity were observed starting from 1 wk, however, the bands continued to appear up to 2 wk, suggesting a sustained biological activity up to 2 weeks. Quantitative analysis of VEGF‐A released from 18NC75‐10P showed a similar trend with total protein release, increasing up to 72 h and decreasing at later time points, although no statistical significance was observed (Figure 2e). For the assessment of biological activity, leachables obtained from 18NC75 and 18NC75‐10P at 24 h post‐incubation were tested for their impact on proliferation and migration of Rat2 cells.^[^ ^30^ ^]^ Upon treating Rat2 cells for 24 h, the leachables from 18NC75‐10P led to higher cell proliferation (Figure 2f),^[^ ^20^, ^31^ ^]^ increased expression of proliferating cell nuclear antigen (PCNA) (Figure 2g), and enhanced cell migration (Figure 2h) compared to control. In contrast, 18NC75 leachables did not demonstrate any significant enhancement, suggesting that L‐PRF in 18NC75‐10P provides the pro‐healing functionalities.^[^ ^20b^ ^]^

### 2.5 Incorporation of IL into 18NC75‐10P for Bactericidal Activity

**Figure 3:**
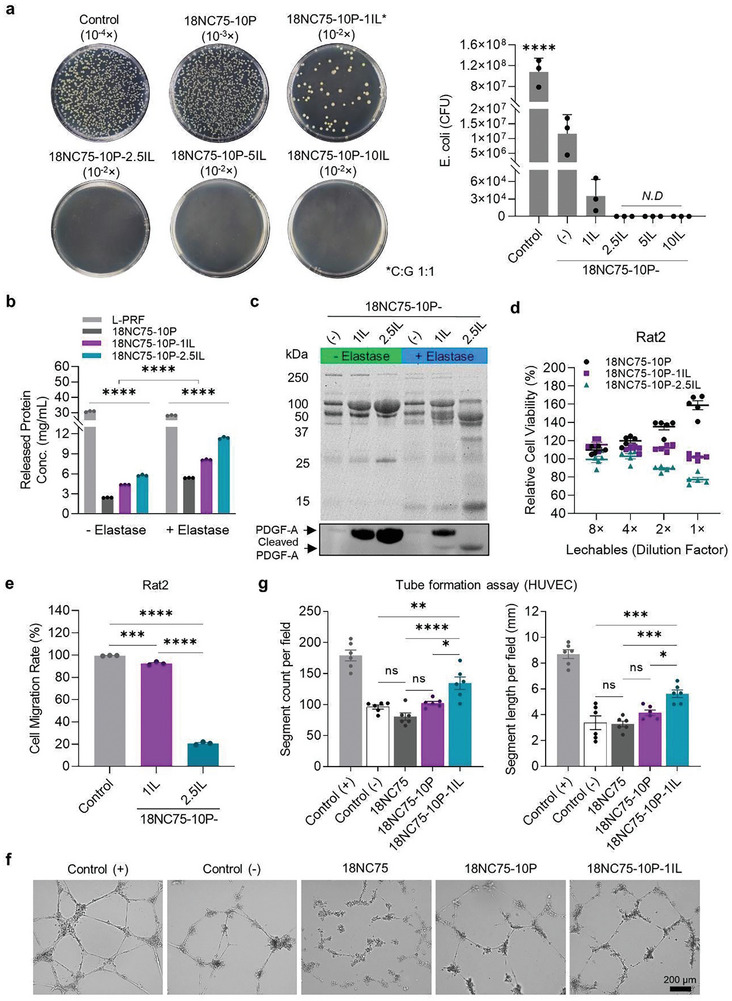
Optimization of IL concentration. a) Images of LB agar plates demonstrating the bactericidal effect of 18NC75‐10P hydrogels incorporated with different concentrations of IL (1, 2.5, 5, or 10% w/w), labeled as 18NC75‐10P‐1IL, 18NC75‐10P‐2.5IL, 18NC75‐10P‐5IL, and 18NC75‐10P‐10IL, respectively. After incubation with each hydrogel, *E. coli* cultures were diluted at indicated factors (10^−4^ to 10^−2^ folds) before inoculation onto LB agar plates. The graph presents the average colony forming units (CFU) counted for each material (*n* = 3). b) Graph depicting protein concentrations released from L‐PRF, 18NC75‐10P, 18NC75‐10P‐1IL, and 18NC75‐10P‐2.5IL, all containing the same amount of L‐PRF, after 24 h of incubation at 37 °C with or without Elastase (*n* = 3). c) SDS‐PAGE and Western blot images for leachables from 18NC75‐10P, 18NC75‐10P‐1IL, and 18NC75‐10P‐2.5IL with and without Elastase. The images illustrate the impact of IL concentration on protein release and enzymatic degradation. d) Assessment of Rat2 cell viability post exposure to leachables from 18NC75‐10P, 18NC75‐10P‐1IL, and 18NC75‐10P‐2.5IL at 1 – 8‐fold dilutions (*n* = 5). e) Graph depicts the migration rate of Rat2 cells treated with leachables from 18NC75‐10P‐1IL and 18NC75‐10P‐2.5IL for 24 h (*n* = 3). f) Representative images from tube formation assay using HUVECs demonstrate the angiogenic effect following treatment with leachables from 18NC75, 18NC75‐10P, and 18NC75‐10P‐1IL compared to controls. g) Quantitative analyses of the tube formation assay presenting segment counts and total segment length measured per field (*n* = 6). Data are mean ± s.e.m.; statistical significance determined by one‐way ANOVA (a, e, and g) or two‐way ANOVA (b) with Tukey's multiple‐comparison test. ns, not significant, *p < 0.05, **p < 0.01, ***p < 0.001, ****p < 0.0001.

IL composed of choline and geranate exhibits potent antimicrobial effects across various bacterial, fungal, and viral species. Its efficacy varies depending on the ratio of choline‐to‐geranate.^[^ ^32^ ^]^ To determine the optimal IL that provides a potent antimicrobial effect, we evaluated the cytotoxicity of ILs with various choline‐to‐geranate ratios (Choline: Geranate (C:G) = 1:1, 1:2, 1:3, and 1:4 molar ratio) against *E. coli* (Figure S15a, Supporting Information) and Rat2 cells (Figure S15b, Supporting Information). The selectivity index, calculated as CC_50_/IC_50_ (where CC_50_ is the 50% cytotoxicity concentration against Rat2 cells, and IC_50_ is the 50% inhibitory concentration against *E. coli*), was highest at C:G = 1:1 (Figure S15c, Supporting Information). Consequently, C:G = 1:1 was chosen for further optimization of the formulation and is now referred to as IL. Different IL concentrations (1, 2.5, 5, 10% w/w) were incorporated into 18NC75‐10P to generate 18NC75‐10P‐1IL, 18NC75‐10P‐2.5IL, 18NC75‐10P‐5IL, and 18NC75‐10P‐10IL, respectively. The bactericidal effect of each material was assessed against *E. coli* through direct contact, as depicted in Figure S16 (Supporting Information). *E. coli* was completely eradicated with IL concentration as low as 2.5% (w/w) in 18NC75‐10P, and 99.9% of *E. coli* was eliminated with only 1% of IL in the formulation (**Figure** **3**a).

### 2.6 Assessment of IL Effects on Growth Factor Release and Degradation

To maximize the biocompatibility while preserving antimicrobial efficacy, 18NC75‐10P‐1IL and 18NC75‐10P‐2.5IL were selected for further characterization. The potential impact of IL incorporation on protein release properties and enzymatic degradation was assessed by incubating L‐PRF, 18NC75‐10P, 18NC75‐10P‐1IL, and 18NC75‐10P‐2.5IL at 37 °C in the presence or absence of elastase for 24 h, and the concentrations of released proteins were quantified (Figure 3b; Figure S17, Supporting Information). Elastase is used in these experiments to simulate the protease‐rich environment often seen with chronic inflammation, such as in ECF; thus, in this experimental setup, the integrity of the leachable proteins is also assessed.^[^ ^33^ ^]^ The released proteins from 18NC75‐10P exhibited an increase corresponding to the increase in IL concentration. This suggests a possibility that IL may weaken interactions among other components, facilitating protein release from the complex. The IL concentration‐dependent increase in zeta potential observed in 18NC75‐10P‐1IL and 18NC75‐10P‐2.5IL compared to 18NC75‐10P implies that IL forms electrostatic complexes with other components (Figure S18, Supporting Information). The presence of elastase led to increased protein release, indicating accelerated degradation of the formulation due to enzymatic activity. These leachables were further analyzed using Western blotting for PDGF‐A detection to determine if the IL concentration in the formulation affects the enzymatic degradation of released proteins with or without elastase (Figure 3c). The appearance of new protein bands ≈15 and 25–37 kDa, accompanied by decreased protein band intensities ≈100 kDa, is attributed to the elastase‐mediated degradation of the released proteins. These alterations in protein fractions became more pronounced with increasing IL concentration. Similarly, PDGF‐A release was evident with increasing IL concentration in the absence of elastase. However, in the presence of elastase, enzymatic degradation was increased and became more pronounced with increasing IL.^[^ ^34^ ^]^ These results suggest that 18NC75‐10P‐1IL is the more favorable formulation balancing protein release with lower degradation in an ECF environment where protease activity is typically high.

### 2.7 Optimization of IL Concentration for Balancing Pro‐Healing and Antimicrobial Effects

Next, Rat2 cells were treated with serially diluted leachables from 18NC75‐10P, 18NC75‐10P‐1IL, and 18NC75‐10P‐2.5IL to determine the formulation that minimizes the cytotoxic effects while maximizing bactericidal effects both resulting from IL (Figure 3d). 18NC75‐10P showed increased cell proliferation at lower dilutions. Conversely, 18NC75‐10P‐2.5IL reduced viability up to ≈23% with undiluted leachables. However, 18NC75‐10P‐1IL treatment did not demonstrate cytotoxicity and had mildly increased cellular proliferation levels at higher dilutions. In addition, 18NC75‐10P‐2.5IL leachables led to a substantial reduction in cell migration with a lower wound closure ratio of 20.64 ± 0.78% compared to control (99.51 ± 0.17%) and 18NC75‐10P‐1IL (92.30 ± 0.84%), consistent with our cell viability assay results (Figure 3e; Figure S19, Supporting Information).

Angiogenesis is an essential part of tissue healing. Next, we evaluated the leachables from 18NC75, 18NC75‐10P, and 18NC75‐10P‐1IL for stimulating angiogenesis using a tube formation assay. Human umbilical vein endothelial cells (HUVECs) seeded on growth factor‐reduced Matrigel (Figure 3f) were treated with the leachables, and tube formation was then quantitatively analyzed (Figure 3g; Figure S20, Supporting Information). Findings revealed that 18NC75‐10P‐1IL promoted tube formation compared to negative control. However, 18NC75 and 18NC75‐10P led to a significantly lower tube formation similar to the negative control. These results suggest that IL in the 18NC75‐10P‐1IL formulation likely led to a greater level of growth factor release to enhance tube formation; this is consistent with greater levels of protein release observed in Figure 3b,c. The rationale for selecting 1% w/w IL is summarized in Figure S21 (Supporting Information).

To assess the release of IL and its bactericidal effect, leachables were collected from 18NC75‐10P‐1IL at various time points (24 h, 48 h, 72 h, and 1 wk) and inoculated with *E. coli*. The leachables showed an enhanced bactericidal effect at later time points, with growth inhibition rates of 71.44% for the 24 h and 82.85% for the 1 wk leachables (Figure S22, Supporting Information). This suggests that IL can be released from 18NC75‐10P‐1IL into surrounding tissue and exert an antibacterial effect when injected into ECF tracts.

### 2.8 Assessment of Mechanical Properties of 18NC75‐10P‐1IL

Given the superior bactericidal effect with minimal cytotoxicity, greater protein release with lower degradation, and enhanced tube formation, 18NC75‐10P‐1IL was selected for further experimentation. To assess the suitability of 18NC75‐10P‐1IL for use in ECF, we re‐evaluated the mechanical property of the optimized formulation. This evaluation included measurement of storage modulus, axial displacement pressure, and injection force. The storage modulus of 18NC75‐10P‐1IL was found to be 57.35 ± 2.79 kPa at shear strain of 10^−1^%, indicating a reduction compared to 74.69 ± 1.67 kPa generated by 18NC75‐10P (Figure S23a,b, Supporting Information). However, the axial pressure required to displace 18NC75‐10P‐1IL (49.58 ± 1.33 kPa) remained notably higher than the pressures encountered during human bowel peristalsis (Figure S23c, Supporting Information). Furthermore, 18NC75‐10P‐1IL exhibited comfortable injectability with both 5F and 7F catheters (12 cm length) (Figure S23d,e, Supporting Information). Based on these findings, 18NC75‐10P‐1IL was considered mechanically suitable for potential use in ECF.

### 2.9 Rat Subcutaneous Implantation Model

**Figure 4:**
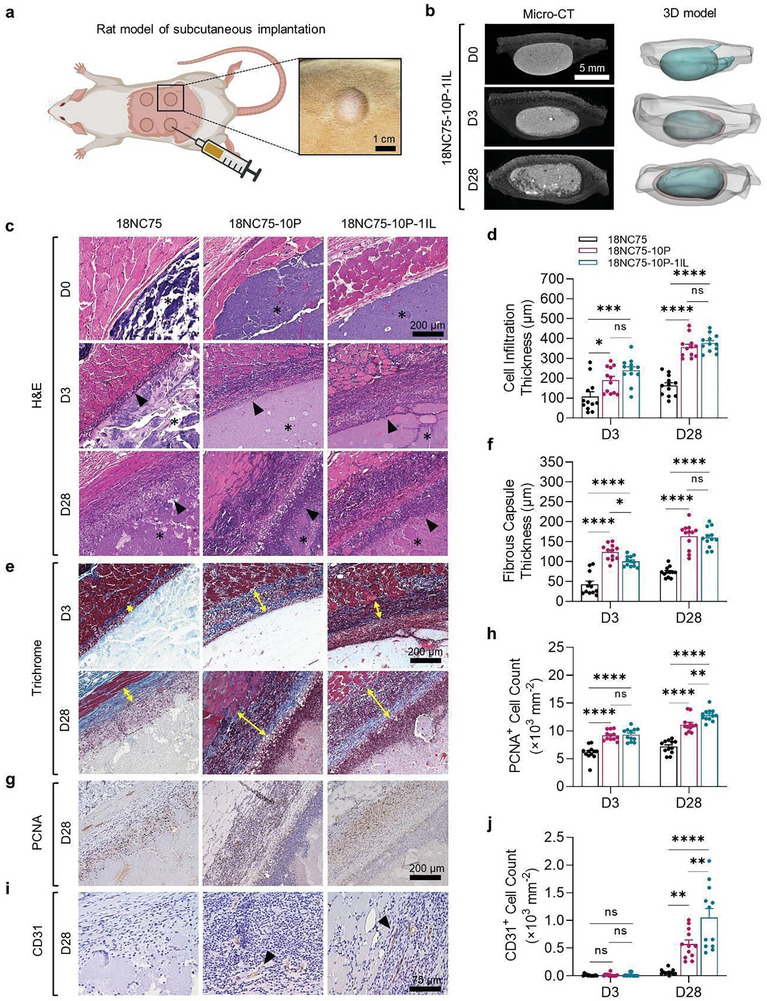
Assessing the local host response in the rat model of subcutaneous implantation. a) A schematic depicting the rat model of subcutaneous implantation and an image of the rat dorsum after subcutaneous injection. Rats received 200 µL hydrogel at four injection sites to assess biocompatibility and host response to 18NC75, 18NC75‐10P, or 18NC75‐10P‐1IL hydrogels. b) Representative sagittal views of reconstructed micro‐CT scans and the 3D‐rendered models illustrating the high opacity of subcutaneously injected 18NC75‐10P‐1IL, compared to the less radiodensity of the surrounding cutaneous tissue at D0, D3, and D28 post‐injection. c) Representative H&E‐stained images of cutaneous tissue sections from rats dorsally injected with 18NC75, 18NC75‐10P, or 18NC75‐10P‐1IL at D0, D3, and D28 post‐injection. Asterisks indicate injected materials, while arrowheads point to cells infiltrated into the injection sites. d) Graph depicts the average cell infiltration zone thickness within the dorsally injected site at D3 and D28 after the injection with 18NC75, 18NC75‐10P, and 18NC75‐10P‐1IL. e) Representative images of Masson's trichrome‐stained histology sections of the injection site at D3 and D28 post‐injection with different hydrogels. Yellow double‐headed arrows indicate fibrous capsule thickness surrounding the injected materials. f) Graph showcasing the average fibrous capsule thickness surrounding each injected hydrogel at D3 and D28. g) Immunohistochemistry images for PCNA^+^ cells surrounding the injected hydrogels at D28 post‐injection. h) Graph depicting PCNA^+^ cell counts surrounding the injected hydrogels at D3 and D28 post‐injection. i) Immunohistochemistry images for CD31^+^ cells surrounding the injected hydrogels at D28 post‐injection (arrowheads). j) Graph showing CD31^+^ cell counts in the tissues surrounding the injected hydrogels at D3 and D28 post‐injection. Data are expressed as mean ± s.e.m., and statistical significance was evaluated using a two‐way ANOVA with Tukey's multiple‐comparison test. ns, not significant, *p < 0.05, **p < 0.01, ***p < 0.001, ****p < 0.0001. The sample size for each analysis was *n* = 12.

Next, we evaluated the biocompatibility and the host response to 18NC75‐10P‐1IL after subcutaneous injection into rats using ultrasound (US), micro‐CT scanning, histomorphology, and blood analyses for markers of systemic injury. Subcutaneous injection of 18NC75, 18NC75‐10P, and 18NC75‐10P‐1IL was performed under the rat dorsal skin in three groups followed by a survival period of 0, 3, or 28 days (**Figure** **4**a). Ultrasound imaging revealed time‐dependent increase in material volume in the 18NC75‐10P and 18NC75‐10P‐1IL treated groups compared to 18NC75 with markedly higher volume at D28 in the 18NC75‐10P‐1IL group (Figure S24a,b, Supporting Information). After necropsy, 3D micro‐CT imaging of explanted tissues containing the injected site at D0 showed no difference between the groups. However, by D3 and D28, there was a gradual increase in the volume at D3 that became significant by D28 in the 18NC75‐10P and 18NC75‐10P‐1IL groups (18NC75: 205.92 mm^3^, 18NC75‐10P: 310.67 mm^3^, and 18NC75‐10P‐1IL: 358.97 mm^3^) (Figure 4b; Figure S25, Supporting Information).

To assess the local tissue response, morphometric analysis of cellular infiltration in the injected site and the surrounding border zone was conducted on H&E‐stained sections. Stained tissue sections displayed enhanced cell infiltration into the injected site starting on D3 for both 18NC75‐10P and 18NC75‐10P‐1IL groups, while minimal cell infiltration was observed for 18NC75 at D28 (Figure 4c; Figure S26, Supporting Information). There were significantly higher counts of cells associated with an increase in the cell infiltration thickness at the margin of the injection zone in both 18NC75‐10P and 18NC75‐10P‐1IL groups compared to 18NC75 at D3 and D28 (Figure 4d; Figure S27, Supporting Information). Masson's trichrome staining indicated a greater thickness of collagen‐rich fibrous capsule in 18NC75‐10P and 18NC75‐10P‐1IL compared to 18NC75 (Figure 4e,f; Figures S26 and S28a, Supporting Information). Immunostaining for PCNA (Figure 4g,h; Figure S28b, Supporting Information) and CD31 (Figure 4i,j; Figure S28c, Supporting Information) exhibited higher cell proliferation and enhanced angiogenesis in 18NC75‐10P and 18NC75‐10P‐1IL. These findings suggest that the volume increase observed on US and Micro‐CT in 18NC75‐10P and 18NC75‐10P‐1IL is potentially due to L‐PRF, which enhanced cell infiltration, proliferation, angiogenesis, and collagen accumulation.

To further evaluate the systemic effects of 18NC75‐10P‐1IL, sera collected at D3 and D28 after subcutaneous injection were analyzed for serum chemistry and serum cytokines/chemokines levels. No notable changes were observed, except for a slight increase in glucose at D3, indicating the biocompatibility of 18NC75‐10P‐1IL (Tables S1 and S2, Supporting Information).

### 2.10 Creating a Rat Model of Anorectal Fistula

**Figure 5:**
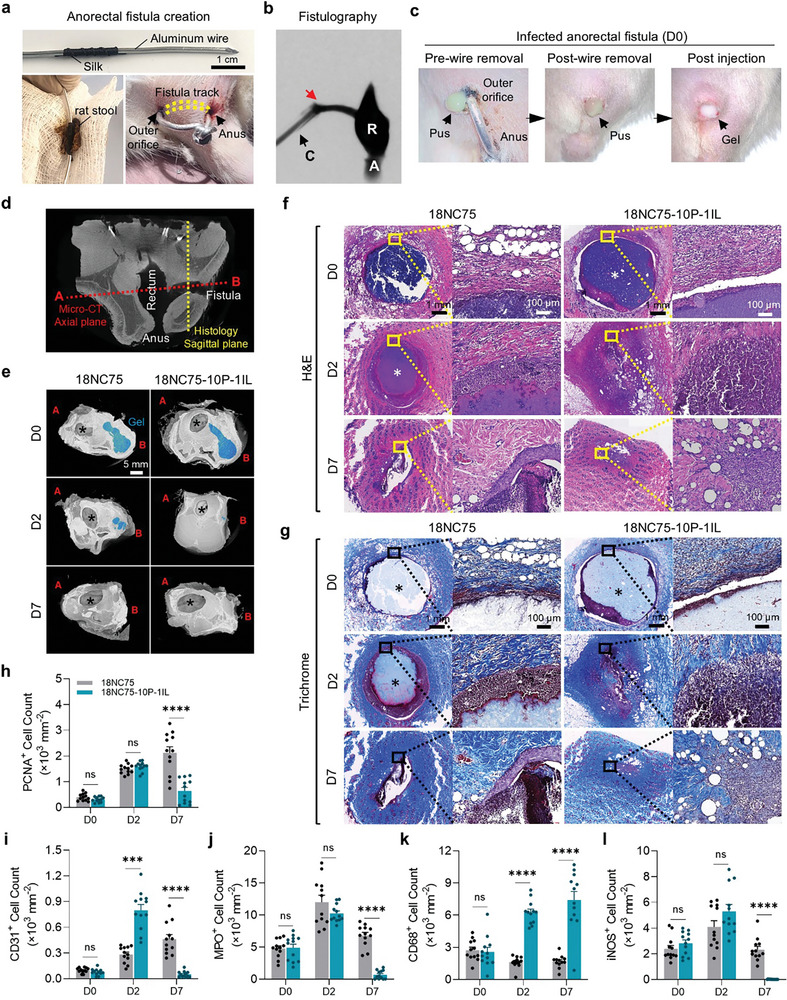
Assessing the host response to hydrogel occlusion in the rat anorectal fistula model. a) Images illustrating the creation of the rat anorectal fistula model; the aluminum wire is wrapped with silk suture, covered with rat feces, and passed through the anorectal area to establish a fistulous tract. b) Digitally subtracted fistulogram acquired during contrast injection demonstrates the fistula tract and the rectum. R (rectum), A (anus), C (catheter), and the red arrow indicates the tip of the catheter at the orifice of the fistula. c) Images depicting an infected anorectal fistula before and after wire removal, and immediately after occlusion with hydrogel (D0). d) Coronal micro‐CT view of rat anorectal fistula after wire removal. The dotted red line indicates the image plane acquired in e, and the dotted yellow line denotes the plane of the histology sections in f. e) Micro‐CT images display segmented hydrogels within the fistula tracts in blue at D0, D2, and D7 after the injection of 18NC75 or 18NC75‐10P‐1IL. f,g) Representative H&E (f) and Masson's trichrome‐stained (g) histology sections of rat anorectal fistulas injected with 18NC75 or 18NC75‐10P‐1IL at D0, D2, and D7. Asterisks indicate the injected hydrogels. h–l) Graphs depict PCNA^+^ (h), CD31^+^ (i), MPO^+^ (j), CD68^+^ (k), and iNOS^+^ (l) cell counts in the area surrounding the fistula tracts at D0, D2, and D7 post‐injection (*n* = 12). Data are mean ± s.e.m.; statistical significance was determined by two‐way ANOVA with Tukey's multiple‐comparison test. ns; not significant, *p < 0.05, **p < 0.01, ***p < 0.001, ****p < 0.0001.

ECF often demonstrates chronic bacterial infection and inflammation, resulting from constant leakage of bowel contents.^[^ ^35^ ^]^ To replicate the clinical aspects of ECF, a rat anorectal fistula model was created using a stool‐coated, silk‐wrapped aluminum wire inserted through the rectum and skin (see Experimental Section for details). (**Figure** **5**a). The fistula region was monitored over a period of up to 28 days with the wire in place (Figure S29, Supporting Information). Gross examination revealed hyperemia surrounding the fistula tract opening on the skin with oozing of pus on both the anus side and on the outer orifice of the fistula. Pus secretion peaked at 7 days after initiation, indicating the successful induction of an infected anorectal fistula. At necropsy, digital subtraction fistulography and micro‐CT imaging confirmed the successful formation of a fistulous tract connecting the rectal space to the skin (Figure 5b; Figure S30, Supporting Information). Histological analyses using H&E, Masson's trichrome, and Gram stain further validated the formation of the fistula tract and detected the presence of bacteria in the fistulous wall (Figure S31, Supporting Information).

### 2.11 Assessment of Host Responses in the Rat Model of Anorectal Fistula

Next, we evaluated the capability of 18NC75‐10P‐1IL to occlude a fistula tract while eliminating infection and promoting a pro‐healing response in a rat model of infected anorectal fistula. On D7 following the creation of the anorectal fistula (designated as D0), the wire was removed, and either 18NC75 or 18NC75‐10P‐1IL was injected using a 5F introducer catheter positioned in the outer orifice of the fistula tract, ensuring complete filling of the tract through the inner orifice within the rectum (Figure 5c).

During the next 7‐day monitoring period after occluding the fistula tract, rats injected with 18NC75‐10P‐1IL showed faster healing of the outer orifice compared to those injected with 18NC75 (Figure S32, Supporting Information). Rats were euthanized at D0, D2, and D7, and tissues housing the anorectal fistula were collected for micro‐CT imaging and histological analysis. Micro‐CT imaging and histology cross sections were acquired to visualize the material in the fistula tract and conduct morphometric analysis (Figure 5d). The 3D volume analysis on micro‐CT scans at D2 revealed a rapid reduction in 18NC75‐10P‐1IL volume, with 2.8% remaining compared to 27.9% of 18NC75 retained within the fistulous tract (Figure 5e; Figure S33, Supporting Information). However, by D7 after occlusion with 18NC75‐10P‐1IL or 18NC75, neither hydrogel was detected in the tissues.

To assess local host responses at a microscale level, histological examination on serially cut histology sections was performed. Examination of H&E‐stained sections illustrated that the fistula tract occluded with 18NC75‐10P‐1IL exhibited extensive cell infiltration by D2 and was fully resolved by D7. In contrast, 18NC75 showed partial cell infiltration into the hydrogel at D2 and incomplete healing of the tract by D7 (Figure 5f). Additionally, Masson's trichrome staining indicated that, for 18NC75‐10P‐1IL, collagen accumulation surrounding the fistula tract peaked at D2 and decreased by D7, suggesting a transition from the proliferation phase to the remodeling phase of the fistula healing process. In contrast, we observed in the 18NC75 group a persistent collagen accumulation that paralleled incomplete healing of the fistula tract at D7 (Figure 5g).^[^ ^36^ ^]^

To investigate if enhanced cellularity and collagen deposition correlate with enhanced markers of cellular proliferation, the tissue sections were immunostained for PCNA. Immunostaining for PCNA revealed a sustained increase in proliferating cells in the 18NC75 group up to D7, in contrast, the sections from the 18NC75‐10P‐1IL group showed that the PCNA expression peaked at D2 and declined by D7 (Figure 5h; Figure S34a, Supporting Information). Similarly, CD31 expression in the 18NC75 group continued to increase peaking at D7, whereas in the 18NC75‐10P‐1IL group, CD31 expressing cells peaked at D2 and sharply declined by D7, returning to a basal level (D0) (Figure 5i; Figure S34b, Supporting Information). The pattern of cellular changes in the tissue indicates that the remodeling phase in the fistula tract was accelerated by the 18NC75‐10P‐1IL treatment.^[^ ^37^ ^]^ To assess the local inflammatory response, sequential slides were immunostained for MPO and CD68 to visualize neutrophils and monocytes/macrophages respectively. Quantitative analysis of MPO^+^ cells demonstrated extensive MPO^+^ cells at D0 that were increased at D2 in both groups. However, the 18NC75‐10P‐1IL group showed a diminished count at D7 below D0 levels (Figure 5j; Figure S35, Supporting Information), indicating that 18NC75‐10P‐1IL effectively resolved the acute inflammatory response.^[^ ^38^ ^]^ Furthermore, CD68^+^ counts showed a sharp increase in the 18NC75‐10P‐1IL group at D2 that persisted at D7, whereas CD68 count did not change in the 18NC75 group throughout the 7‐days after occlusion (Figure 5k; Figure S36a, Supporting Information). To examine the phenotype of the CD68^+^ cells, the sections were stained for iNOS protein, a marker of pro‐inflammatory macrophages. The levels were comparable at D0 and D2 in both groups but exhibited a marked decrease in the 18NC75‐10P‐1IL treated fistula at D7, which was significantly lower than the 18NC75 group (Figure 5l; Figure S36b, Supporting Information).^[^ ^39^ ^]^ These histological findings indicate that the combination of L‐PRF and IL in the 18NC75‐10P‐1IL formulation plays a critical role in anti‐inflammatory and pro‐healing activities. There was no change in the body weight of rats in both groups after 7‐day survival (Figure S37, Supporting Information). Analysis for complete blood count, serum chemistry, and serum cytokines/chemokines also showed no notable changes in the 18NC75‐10P‐1IL group during the survival period, indicating no systemic adverse reactions (Tables S3–S11, Supporting Information).

### 2.12 Evaluation of 18NC75‐10P‐1IL in a Modified Rat Anorectal Fistula Model

Next, we investigated the cause of the rapid decrease in 18NC75‐10P‐1IL volume detected in the fistula tract on micro‐CT and histology. We hypothesized that this could be related to the extrusion of the hydrogel from the tract after occlusion due to contraction and pressure exerted by surrounding tissue, or due to a rapid breakdown of hydrogels during the course of healing processes mediated by the infiltrating inflammatory cells. To test this hypothesis, the infected anorectal fistula tract was occluded with 18NC75‐10P‐1IL then the outer opening was sealed using an absorbable 5‐0 Vicryl suture to prevent the possibility of material leakage and extrusion from the tract during the survival period (Figure S38, Supporting Information). Micro‐CT analysis revealed the presence of the hydrogel at D2; however, no hydrogel was detected at D7, which is consistent with our previous results, where the outer fistula tract orifice remained open without a suture (Figure S39, Supporting Information). Histological evaluation of H&E and Masson's trichrome stained sections indicated a gradual increase in cell infiltration into the injected hydrogel, resulting in high cell infiltration and a significant reduction in tract size by D7 (Figure S40, Supporting Information). Immunohistochemical analyses for PCNA, CD31, MPO, CD68, and iNOS (Figure S41a–e, Supporting Information) showed a similar trend to the previous results in Figure 5f–j, indicating a transition to the healing process from D2 to D7.

### 2.13 Assessment of Antimicrobial Effect in the Rat Anorectal Fistula Model

**Figure 6:**
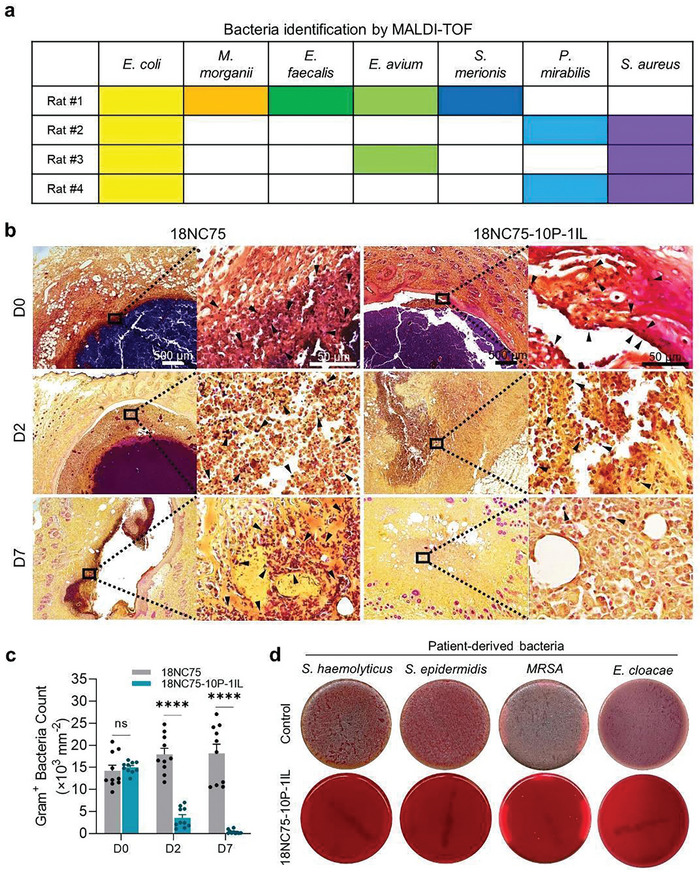
Assessing Bactericidal Effects of 18NC75‐10P‐1IL in vivo and in vitro. a) MALDI‐TOF mass spectrometry analysis displaying the bacteria identity in pus collected from the outer orifices of anorectal fistula tracts in rats (*n* = 4). b) Representative Gram stain images of tissue sections from rat anorectal fistulas injected with 18NC75 or 18NC75‐10P‐1IL at D0, D2, and D7 post‐injection. Arrowheads indicate Gram^+^ bacteria stained in purple. c) Quantitative analyses of Gram^+^ bacteria count in areas surrounding the fistula tracts at D0, D2, and D7 post‐injection with 18NC75 or 18NC75‐10P‐1IL (*n* = 10). d) Images of blood agar plates illustrating the robust bactericidal effect of 18NC75‐10P‐1IL against selected patient‐derived bacteria, including *S. haemolyticus*, *S. epidermidis*, *MRSA*, and *E. cloacae*, tested in direct contact with the hydrogel compared to the control. Data presented as mean ± s.e.m.; statistical significance determined by two‐way ANOVA with Tukey's multiple‐comparison test. ns, not significant, *p < 0.05, **p < 0.01, ***p < 0.001, ****p < 0.0001.

Seven days after creating the anorectal fistula model in rats, pus was collected from the fistula tract for the identification of microorganisms using Matrix‐assisted laser desorption ionization‐time of flight mass spectrometry (MALDI‐TOF). The analysis revealed seven strains of bacteria, with two prominent bacteria strains (*E. coli* and *S. aureus*) correlating with infected ECF fistula in humans (**Figure** **6**a).^[^ ^8^, ^40^ ^]^ To assess the in vivo antimicrobial properties of 18NC75‐10P‐1IL, tissue sections from rat anorectal fistulas treated with 18NC75‐10P‐1IL were compared with those treated with 18NC75 (lacking IL) using Gram staining (Figure 6b). At D0, before occlusion, both groups exhibited a comparable count of Gram^+^ bacteria in the fistula wall. However, by D2 and D7, the 18NC75‐10P‐1IL group showed a substantial decrease in bacteria count with a reduction of 76.4% and 97.5%, respectively, compared to D0 (Figure 6c). In contrast, the fistula treated with 18NC75 had an elevated bacterial count that persisted up to D7. The same trend was observed with Grocott's methenamine silver (GMS) staining which is commonly used for detecting fungal stains (Figure S42, Supporting Information). These results suggest that 18NC75‐10P‐1IL is effective in ameliorating the pathogenic burden of an infected anorectal fistulas or ECF.

### 2.14 Antimicrobial Effect Against Patient‐Derived Antibiotics‐Resistant Bacteria

Next, we evaluated the efficacy of 18NC75‐10P‐1IL against prominent highly resistant bacteria common in infected human ECF, namely *S. haemolyticus, S. epidermidis, MRSA*, and *E. cloacae*.^[^ ^41^ ^]^ Each bacterial strain (7.5 × 10^7^ CFU) was inoculated either into an empty well (Control) or onto 18NC75‐10P‐1IL and incubated overnight. To verify bacterial growth, an aliquot from each culture was inoculated onto blood agar plates for another overnight incubation. In contrast to the control group, no bacterial colonies were observed in any of the tested strains that came in contact with 18NC75‐10P‐1IL, demonstrating a potent bactericidal effect against clinically challenging‐to‐treat bacteria (Figure 6d). Given that chronic infection is a major barrier to the effective healing of ECFs,^[^ ^42^ ^]^ the antimicrobial properties of 18NC75‐10P‐1IL, validated through rigorous evaluation in in vitro studies, an in vivo rat anorectal fistula model, and against patient‐derived antibiotics‐resistant bacteria, underscore its strong translational potential for addressing the persistent clinical challenges in ECF treatment.

## 3 Conclusion

Here, we engineered a multi‐functional injectable biomaterial (18NC75‐10P‐1IL) specifically designed to facilitate durable and aseptic occlusion of fistula tracts, thereby expediting the healing process. Rigorous mechanical optimization rendered 18NC75‐10P‐1IL easily injectable using catheters, while also demonstrating resilience against repeated external pressures. In vitro assessment confirmed its pro‐healing functionalities and antimicrobial properties. Extensive evaluation of biocompatibility and host responses using various rat models demonstrated that 18NC75‐10P‐1IL is not only biocompatible but also exerts simultaneous anti‐inflammatory and antimicrobial effects. These results led to accelerated fistula healing compared to a control material (18NC75), suggesting potential therapeutic benefit in managing infected ECF and anorectal fistulas in humans.

This study had several limitations. A clinically relevant rat model for chronic ECF is lacking due to the challenge of inducing chronic infection and delayed healing in rats, given their high resistance to pathogen infections.^[^ ^43^ ^]^ Despite observing the superiority of 18NC75‐10P‐1IL, our attempt to simulate a chronically infected anorectal fistula in rats led to closure of the outer orifice of the fistula tract within ≈7 days. Future studies will focus on establishing a large animal model that is prone for chronic infection and delayed healing, mimicking the clinical scenario more closely and allowing for a more thorough evaluation of the therapeutic potential of 18NC75‐10P‐1IL. Upon successful evaluation in this model, the focus will shift to clinical trials in patients with ECFs.

## 4 Experimental Section

### Lyophilized Platelet‐Rich Fibrin (L‐PRF) Preparation

Whole blood was collected from Yorkshire pigs and aliquoted into BD Vacutainer Serum tubes (BD, Franklin Lakes, NJ, USA) and immediately centrifuged at 900 × g for 12 min at room temperature (Scilogex DM0412, Rocky Hill, CT, USA). The resultant straw‐colored PRF layer was collected, subsequently frozen at ‐80 °C, and freeze‐dried using a lyophilizer (Labconco FreeZone, Kansas City, USA) for 2 days.

### Ionic Liquid (IL) Preparation

IL was prepared from choline and geranate through a salt metathesis process according to previously described methods at equal molar ratio (C:G 1:1).^[^ ^25b^ ^]^ Neat geranic acid (Sigma‐Aldrich, St. Louis, MO) was purified via five cycles of repeated recrystallization at ‐80 °C in an acetone bath. The purified geranic acid was then mixed with an equal molar ratio of choline bicarbonate (Sigma‐Aldrich) and allowed to react at room temperature with constant stirring until the cessation of the CO_2_ byproduct. Subsequently, residual H_2_O was eliminated using a rotary evaporator (R‐300, Buchi, New Castle, DE) at 60 °C for 1 h. The resulting chemical structure of the synthesized neat IL was characterized using attenuated total reflectance‐Fourier transform infrared (ATR‐FTIR) spectroscopy (Lumos II with Alpha II add‐on, Bruker, Kontich, Belgium).

### Shear‐Thinning Biomaterial Preparation

The initial step involved the preparation of a 30% (w/v) nanosilicate (NS) hydrogel, achieved through the efficient mixing of Laponite‐XLG (BYK USA, Inc., Gonzales, TX, USA) with ice‐cold ultrapure water, using a Speed Mixer (FlackTek, Inc., Landrum, SC, USA). Subsequently, the NS hydrogel was mixed with solubilized gelatin from porcine skin (Type A, ≈300 g Bloom, Sigma‐Aldrich), which melted in ultrapure water at 60 °C. Subsequently, this gelatin solution was combined with 30% NS, L‐PRF, and IL in a predetermined weight ratio. The entire mixture underwent thorough mixing using the Speed Mixer to yield a homogeneous shear‐thinning biomaterial.

### Rheological Tests

A rotational rheometer (MCR 302, Anton Paar, Austria) equipped with a 25 mm parallel sand‐blasted plate (PP25/S, Anton Paar, Austria) was used for all rheological assessments. The gap between the upper and bottom plates was set at 1 mm, and the temperature was maintained at 37 °C. The solvent trap was filled with water to prevent drying of the tested materials. Viscosity measurements were conducted over a shear rate range of 10^−3^ – 10^3^ s^−1^. To assess storage and loss modulus, large amplitude oscillation sweeps (LAOS) were performed under a shear strain range between 10^−2^ to 10^2^%, while maintaining a constant angular frequency of 10 rad s^−1^. In the thixotropy test, cyclic strain changes were applied, alternating between low‐magnitude (0.5%) for 2 min and high‐magnitude (100%) for 1 min, at a fixed angular frequency of 10 rad s^−1^.

### Fabrication of 3D‐Printed Fistula‐Mimicking Model

To create 3D‐printed fistula‐mimicking models that replicate the irregular surfaces of fistulous tracts in humans, a cylindrical digital model with a total length of 60 mm, made up of a series of cylinders with varying widths of 1 to 2 mm and diameters of 2.5 to 3.5 mm, was designed using Autodesk Fusion 360 software (Autodesk, San Francisco, CA). The cylindrical digital model was then exported as a Standard Tessellation Language (STL) file to 3‐Matics software (Materialise NV, Leuven, Belgium). To create a fistula‐mimicking model for the axial displacement pressure test, a digital box model measuring 30 mm × 15 mm × 60 mm was generated to encompass the cylindrical model and the cylindrical model was subtracted from the digital box model, leaving an open space that reflects the fistulous tract. Two digital models of Luer lock end block were imported into 3‐Matrics software and aligned with the subtracted digital box model at both ends (Figure S14a, Supporting Information). To create a fistula‐mimicking model for the compressive displacement pressure test, the cylindrical digital model with a total length of 30 mm was imported into 3‐Matics software. Subsequently, a digital box model measuring 28 mm × 15 mm × 30 mm was generated to encompass the cylindrical model and the cylindrical model was subtracted from the digital box model (Figure S11, Supporting Information). The resulting fistula‐mimicking models were exported as STL files. These models were imported into GrabCad software (Stratasys, Rehovot, Israel) and the Stratasys J750 Digital Anatomy Printer (DAP, Stratasys) was used for printing. To mimic the stiffness of soft tissue, anatomical presets were utilized, employing a predetermined mixture of resins, including Agilus30 Clear (FLX935, Stratasys), Bone Matrix (RGD516, Stratasys), Gel Matrix (FLG110, Stratasys), Tissue Matrix (MED310c, Stratasys), Vero Magenta (RGD851, Stratasys), and Vero Pure White (RGD837, Stratasys). The Subcutaneous Fat preset was chosen for the subtracted box models, and the Luer lock end blocks were set to print in pure Bone Matrix resin. Following printing, excess support material was manually removed, and the models were submerged in a 2% Sodium Hydroxide solution to dissolve any remaining support material. Subsequently, after rinsing with water and drying, the models were prepared for use in the experiment.

### Perpendicular Displacement Pressure

A fistula‐mimicking model without Luer locks was filled with each test material and was subjected to 10 cyclic compressions. The compression conditions included ranges of 0 – 20 kPa or 0 – 60 kPa, applied at rates of 40 and 120 kPa min^−1^, respectively, using Compression testing system equipped with a 100 N loading cell (Instron 5942, Instron Corp., Norwood, MA, USA). The compression pressure ranges of 0 ‐20 kPa and 0 – 60 kPa were chosen to mimic bowel peristaltic pressure and pressure causing muscle necrosis, respectively. The models were scanned with micro‐CT (Skyscan 1276, Bruker, Kontich, Belgium) before and after the compression cycles, and the total volume of each material within the model tract was quantified using CTAn software (Bruker, Kontich, Belgium) after applying appropriate threshold levels to segment the material from the surrounding block matrix. The remaining volume % was calculated as V_C_/V_0_ × 100, where V_0_ represents the initial volume before compression, and V_C_ represents the volume after compression.

### Axial Displacement Pressure

A fistula‐mimicking model with Luer locks was linked to a 60 mL syringe (BD, Franklin Lakes, NJ, USA) and secured to a syringe pump (GenieTouchTM, Kent Scientific Corp., CT, USA). A pressure sensor (PX409, Omega Engineering, Inc., Norwalk, CT, USA) was attached via tubing to each end of the model through the Luer locks, establishing a closed system. Subsequently, a 1 mL aliquot of each material was introduced into the fistula‐mimicking model. PBS was then infused into the model utilizing the syringe pump at a flow rate of 50 mL min^−1^. The pressure applied to the model was continuously monitored and recorded using the pressure sensor and Digital Transducer Application software (Omega Engineering, Inc., Norwalk, CT, USA). The maximum pressure required to displace the test materials from the model was measured and defined as the displacement pressure.

### Compressive Young's Modulus

Rat tissues, ≈1.5 cm thick, were harvested from the dorsal region and trimmed to a size of 3 cm (width) × 3 cm (length). The rat tissue and the fistula‐mimicking model (with no Luer locks) underwent compression at a strain rate of 5 mm min^−1^ using compression testing equipment (Instron 5942, Instron Corp., Norwood, MA, USA). The resulting strain‐stress flow curves were recorded with Bluehill3 software (Instron Corp., Norwood, MA, USA). Young's modulus was calculated as stress/strain at a strain of 0.1, within the linear elastic region generated by each material.

### Injection Force

Medallion syringes (1 mL) (Merit Medical Systems, Inc., South Jordan, UT, USA) or BD syringes (BD, Franklin Lakes, NJ, USA) were filled with test materials and connected to 5, 6, or 7F catheters (Cook Medical, Bloomington, IN, USA) with a length of 12 cm. The syringes underwent compression at a rate of 60.17 mm min^−1^ using Compression testing equipment (Instron 5942, Instron Corp., Norwood, MA, USA), and the force required to inject the material through the catheter was recorded using Bluehill3 software (Instron Corp., Norwood, MA, USA). The break‐loose force denotes the maximum force necessary to initiate movement of the plunger from its resting position (peak force), and the injection force represents the force required to sustain the movement (force at plateau).

### Hydrogel Extraction

Each hydrogel was combined with 0.01 mm HCl in water at 1:9 w/w ratio and sonicated with a 60 Sonic Dismembrator (Thermo Fisher Scientific, Waltham, MA, USA) for 1 min on ice. The resulting homogenate was mixed with Protease Inhibitor Cocktail tablet (Roche, Basel, Switzerland) + RIPA buffer (Abcam, Cambridge, UK) at 1:1 v/v ratio and placed on ice for 30 min. Subsequently, the mixture was centrifuged at 15000 × g for 15 min (accuSpin Micro 17, Thermo Fisher Scientific, Waltham, MA, USA). The supernatant was collected, and the amount of extracted protein was analyzed using BCA Protein Assay Kit (Thermo Fisher Scientific).

### Preparation of Hydrogel Leachables and Protein Release Test

To prepare hydrogel leachables for protein release testing, aliquots of each hydrogel were submersed in DPBS at a concentration of 40 w/v% and then incubated at 37 °C. At each predetermined time point (24, 48, 72 h, 1, 2 wk), the samples were centrifuged at 2400 × g for 15 min (Centrifuge 5804 R, Eppendorf, Hamburg, Germany), and the supernatants were collected. The total amount of protein in the leachables was measured using BCA Protein Assay Kit, and the percentage of released protein was determined by comparing it to the amount of total protein of L‐PRF included in the hydrogels. The amount of VEGF‐A in the leachables was measured using a porcine VEGF‐A ELISA Kit (RAB1135, Sigma‐Aldrich), following the manufacturer's instructions.

### Polyacrylamide Gel Electrophoresis (SDS‐PAGE)

The extracted protein samples or leachables were diluted and mixed with 10% β‐Mercaptoethanol (Sigma‐Aldrich, St. Louis, MO, USA) + 4× Laemmli Sample Buffer (Bio‐Rad, Hercules, CA, USA) at a 3:1 v/v ratio, followed by boiling for 5 min. Subsequently, either 20 µg of the denatured total protein extracts, or 10 µL of the denatured leachables were loaded into individual wells of an 8 – 16% sodium dodecyl sulfate‐polyacrylamide gel (Bio‐Rad, Hercules, CA, USA) and resolved through electrophoresis at 100 V for 1.5 h. Precision Plus Protein Kaleidoscope Protein Standards (Bio‐Rad) served as a reference to determine the size of the protein band fractions. Following protein fractionation, the gels were incubated in a transfer buffer (DI water: Methanol: 10× Tris/Glycine Buffer (Bio‐Rad) = 7:2:1 volume ratio) for 15 min and subsequently imaged using Gel Doc XR+ Imager (Bio‐Rad). Subsequently, the resolved proteins in the gels were transferred onto a PVDF membrane using Trans‐Blot Turbo Transfer Pack (Bio‐Rad). To minimize non‐specific antibody binding, the membranes were incubated in a protein blocking buffer comprising 5% Blotting‐Grade Blocker (Bio‐Rad) in phosphate buffered saline and 0.1% Tween‐20 (PBS‐T) for 1 h at room temperature. This step was followed by an overnight incubation at 4 °C with antibodies targeting PDGF‐A (1:1000, ABIN2774382, antibodies‐online), VEGF‐A (1:1000, PAA143Po01, Cloud‐Clone Corp.), or GAPDH (1:2000, ab70699, Abcam) diluted in the blocking buffer. After three rinses in PBS‐T, the membranes were incubated with an HRP‐conjugated Goat anti‐Rabbit secondary antibody (1:1000, ab97051, Abcam) in the blocking buffer for 1 h at room temperature, followed by rinsing in PBS‐T. The membranes were then reacted using the SuperSignal West Femto Maximum Sensitivity Substrate (Thermo Fisher Scientific, MA, USA), and the specific protein bands were visualized using the Amersham Imager 680 (Global Life Sciences Solutions USA LLC, MA, USA).

### Preparation of Hydrogel Leachables for In Vitro Assays

Various hydrogel preparations were immersed in serum‐free DMEM at 15 w/v% concentration and incubated at 37 °C for 24 h. After incubation, the sample underwent centrifugation at 2400 × g for 15 min. The resulting supernatant was collected and further supplemented with 10% FBS.

### Cell Migration

Rat fibroblast cells (Rat2, ATCC Manassas, VA) were cultured in DMEM (Thermo Fisher Scientific), supplemented with 10% fetal bovine serum (FBS) and 1% penicillin/Streptomycin, and incubated at 37 °C in a 5% CO_2_ cell culture incubator. The cells were initially seeded at a density of 2 × 10^4^ cells per well inside each compartment of the culture‐Insert 2‐Well in a 35 mm µ‐Dish (Ibidi USA Inc., Fitchburg, WI, USA), and allowed to incubate for 24 h. Following the incubation period, the insert was removed to create a consistent gap area between the two chambers separating the cell monolayers. The culture media were then replaced with 2 mL of fresh complete growth media (Control) or hydrogel leachables in growth media. Brightfield images were captured to calculate the gap area at 0 h (baseline). After 24 h, the cells were labeled with CellTracker Green CMFDA (Thermo Fisher Scientific) and imaged using fluorescence microscopy (Evos FL Auto 2, Thermo Fisher Scientific). The remaining gap area in each µ‐Dish was measured using QuPath software (University of Edinburgh), and the migration rate was calculated relative to baseline as follows:

(1) $$\left[1-\left(\left(\mathrm{Are}a_{0h}-\mathrm{Are}a_{24h}\right)/\mathrm{Are}a_{0h}\right)\right]\times 100\left(\%\right)$$

### Cell Proliferation

Rat2 cells in complete growth media were seeded at a density of 5 × 10^3^ cells per well in 96‐well tissue culture plates (Celltreat, Pepperell, MA, USA) and incubated for 24 h. Subsequently, the media was replaced with either fresh complete growth media (Control) or hydrogel leachables, followed by an additional 24 h of incubation. Cell viability was assessed with WST‐1 Cell Proliferation Assay Kit (Cayman Chemical, Ann Arbor, MI, USA), in accordance with the manufacturer's instructions.

### Immunofluorescent Detection of PCNA Expression in Rat2 Fibroblasts

Rat2 cells were seeded on μ‐slide 8‐well chambers (Ibidi, 80 826) at a density of 1.5 × 10^4^ cells per well and incubated overnight. The control group was treated with growth media, whereas the experimental groups were incubated with prepared leachables of 18NC75 or 18NC75‐10P in serum‐free DMEM supplemented with 10% FBS. After 24 h treatment, cells underwent rinsing in DPBS, fixation in 4% paraformaldehyde, permeabilization using 0.1% Triton X‐100 in DPBS, blocking with 1% bovine serum albumin (BSA) in DPBS, incubation with rabbit anti‐PCNA monoclonal IgG (1:100, Rb mAb anti‐PCNA, AB92552) in 1% BSA, followed by incubation with Alexa Fluor 594 conjugated goat anti‐rabbit IgG (R37117). Subsequently, cells were covered using Vectashield Hardset Antifade mounting medium (H‐1500, Victor Laboratories), and imaged using fluorescence microscopy (Evos FL Auto 2, Thermo Fisher Scientific).

### Tube Formation Assay

Human umbilical vein endothelial cells (HUVECs, ATCC) were seeded onto growth factor‐reduced Matrigel (Corning, NY, USA)‐coated 96‐well tissue culture plates (Celltreat) at a density of 1.5 × 10^4^ cells per well. Designated wells were treated with full growth factors‐supplemented cell culture medium, EBM‐2 (Control (+)), non‐supplemented EBM‐2 (Control (‐)), or leachables prepared in non‐supplemented EBM‐2 (Experimental samples). After 18 h of incubation, the wells were imaged using brightfield microscopy (Evos FL Auto 2, Thermo Fisher Scientific), and analyzed using the Angiogenesis Analyzer plug‐in of ImageJ software. The angiogenesis was assessed based on parameters such as number of Junctions, indicating multi‐intersection points with three or more furcated branches in angiogenic structures; and Segment, denoting an angiogenic capillary with two ends connected to two junction points (Figure S20, Supporting Information).^[^ ^44^ ^]^

### Evaluating Bacterial Fractional Viability in Response to IL Treatment

GFP‐expressing Escherichia coli (GFP‐E. coli, 25922GFP, ATCC) were cultured in LB broth microbial growth medium (Thermo Fisher Scientific) at a concentration of 4 × 10^7^ CFU mL^−1^. The bacterial culture was then treated with various choline (C) and geranic acid (G) ILformulations at different molar ratios of C:G (including 1:1, 1:2, 1:3, 1:4), which were subsequently serially diluted to achieve final concentrations ranging from 0 to 20%. The treated cultures were incubated overnight at 37 °C. Following incubation, the bacterial cultures were transferred to 96‐well black solid plates (Celltreat). Fluorescence intensity in each well was measured using excitation at 475 nm and emission at 507 nm with a SpectraMax iD5 microplate reader (Molecular Devices, San Jose, CA, USA). Relative cell viability was determined based on fluorescence intensity measurements, and the 50% cytotoxicity concentration (CC50) was calculated using nonlinear regression sigmoidal curve analysis performed with GraphPad Prism 9 software.

### Assessing the Cytotoxicity of IL in Rat Fibroblasts

Rat2 cells cultured in complete growth media were seeded at a density of 5 × 10^3^ cells per well in 96‐well tissue culture plates (Celltreat) and incubated for 24 h. The media was then replaced with either fresh complete growth media (Control) or growth medium containing various ILs of different molar ratios (C:G 1:1, 1:2, 1:3, 1:4). The IL containing mediums were serially diluted to achieve a concentration range of 0 – 5%. Following treatment, the cells were incubated for additional 24 h, and cell viability was assessed using the water‐soluble tetrazolium‐1 (WST‐1, Cayman Chemical) reagent as per the manufacturer's instructions. The concentration that induces 50% cytotoxicity was extrapolated from nonlinear regression sigmoidal curve using GraphPad Prism 9 software.

### Evaluating the Antimicrobial Efficacy of Hydrogels under Contact Conditions In Vitro

A 500 µL aliquot of each hydrogel was added into designated wells in a 12‐well tissue culture plate (Celltreat) and centrifuged to ensure even distribution. To prevent drying during the incubation period, empty wells were filled with sterile water. Subsequently, 4 × 10^4^ CFU of *E. coli* in 50 µL of LB broth was inoculated onto each hydrogel in selected wells, followed by overnight incubation at 37 °C. The supernatants containing the *E. coli* were collected by rinsing ten times with 1 mL LB broth. The collected *E. coli* aliquots were diluted to 10^2^ – 10^4^ folds with LB broth, and 100 µL of each sample was inoculated onto LB agar plate. The plates were incubated overnight at 37 °C, and the number of colonies were counted and compared to the control group.

### Evaluating Protein Release Kinetics from Hydrogels with and without Elastase

A 40 mg of L‐PRF preparation, or a 400 mg aliquot of each hydrogel (18NC75‐10P, 18NC75‐10P‐1IL, or 18NC75‐10P‐2.5IL, each containing 40 mg of L‐PRF) were immersed in 1 mL of PBS, with or without 0.6 units of Elastase (E1250, Sigma‐Aldrich, St. Louis, MO, USA), and then incubated at 37 °C for 24 h. Following the incubation period, the supernatants were collected and underwent centrifugation at 2400 ×g for 15 min. The concentration of released protein was measured using the BCA Protein Assay Kit (BioRad). Subsequently, 20 µg of total protein from each sample was loaded onto an 8 – 16% sodium dodecyl sulfate‐polyacrylamide gel for protein fractionation and analyzed via Western blot for PDGF‐A levels as previously described.

### Evaluating the Zeta Potential of the Hydrogels

Aliquots of each hydrogel were dispersed in distilled water to attain a concentration of 10% (w/v) through vigorous vortexing. The zeta potential of the resulting suspended mixture was then measured using folded capillary cells (DTX1070, Malvern) and the Zetasizer Ultra instrument (Malvern).

### Characterizing Hydrogels Porosity using Scanning Electron Microscopy

Aliquots of hydrogels were frozen at ‐80 °C overnight and then subjected to freeze‐drying using a lyophilizer (Labconco FreeZone, Kansas City, USA) for 24 h. A sample of each lyophilized hydrogel was placed on top of a carbon mount pin (16 711, Ted Pella, Inc., Redding, CA, USA) over a carbon conductive adhesive tape (PELCO Tabs, 16084‐2, Ted Pella). Subsequently, the lyophilized material was sputter coated with gold/palladium using a Vacuum coater (Leica EM ACE200, Leica Microsytems, Wetzlar, Germany). The coated samples were imaged using a scanning electron microscope (JCM‐6000PLUS Benchtop SEM, JEOL, Tokyo, Japan), and measurements of pore size and porosity were conducted on the acquired images using ImageJ software. Pore size was analyzed with 46 measurements, and the porosity was measured in three individual fields.

### Evaluating the Antimicrobial Efficacy of 18NC75‐10P‐1IL Leachables

An 800 mg aliquot of 18NC75‐10P‐1IL was immersed in 2 mL of saline (0.9% Sodium Chloride Irrigation USP, Baxter), and incubated at 37 °C for predetermined durations (24 h, 48 h, 72 h, and 1 wk). After the incubation period, the supernatants were collected by centrifugation at 2400 ×g for 15 min. Subsequently, 4 × 10^4^ CFU of *E. coli* were inoculated into 1 mL of saline (Control) or each leachables, followed by overnight incubation at 37 °C. The *E. coli* aliquots were then diluted to 10^4^ folds with saline, and 100 µL of each sample was inoculated onto LB agar plates. The plates were incubated overnight at 37 °C, and the number of colonies was counted and compared to the control group.

### Rat Model of Subcutaneous Implantation of Hydrogels

All animal procedures were approved by the institutional animal care and use committee (IACUC) at Mayo Clinic, under protocol number A00004804‐19‐R22. Male Sprague Dawley rats (Charles River Laboratories, Wilmington, MA, USA) at three to five weeks of age, received subcutaneous injections with 0.2 mL aliquot of hydrogel at four locations in the dorsal region, comprising 18NC75, 18NC75‐10P, or 18NC75‐10P‐1IL under isoflurane anesthesia. The rats survived for 28 days after the injection and the injection sites were serially imaged using ultrasound (ACUSON S2000, Siemens Medical Solutions USA, CA, USA). The subcutaneous hydrogel volume in the subcutaneous space was calculated based on measurements of three axis of an ellipsoid shape using the following formula: 4/3 × π × (a/2) × (b/2) × (c/2), where a, b, and c represent the lengths of each axis of the hydrogel, measured in the ultrasound images. Subgroups of rats were euthanized at 0, 3, or 28 days post‐injection and the tissue of each injection site was harvested then fixed in 10% buffered formalin (Fisher Healthcare, Waltham, MA, USA). The fixed tissues were scanned using Skyscan 1276 micro‐CT scanner (Bruker, Waltham, MA) using the following parameters: 40 Volts, 200 µA, 0.4 angular rotation, 2 frame averaging, at 180‐degree axial rotation, and 10 µm voxel resolution. The image stacks were reconstructed using the InstaRecon reconstruction software after applying correction for misalignment, ring artifacts, and beam hardening artifacts to create 3D volumes. 3D volume analysis of the implanted hydrogels was performed using CTAn software. Digital Imaging and Communications in Medicine (DICOM) image stacks were loaded into Materialise Mimics software (Materialise NV, Leuven, Belgium) to create 3D model rendering of each injection site after segmentation. Segmentation masks were generated for both the tissue sample and injected hydrogel based on their density to create respective parts using standardized global threshold values. The segmented masks were then manually verified and adjusted to ensure the accuracy of the segmented models. These segmented models were exported as Standard Tessellation Language (STL) files. The STL files of each model were imported into 3‐Matics software (Materialise NV, Leuven, Belgium), and different colors were assigned to each part for differentiation. Post‐processing steps were performed to eliminate any artifacts in the models. Transparency was adjusted, and the models were manually aligned to create a collage that exhibited all segmented parts.

### Establishing a Rat Model of Anorectal Fistula

The animal procedures in this study received approval from the institutional animal care and use committee (IACUC) at Mayo Clinic, under protocol number A00006297‐21‐R24. Five‐week‐old male Sprague Dawley rats were employed from Charles River Laboratories (Wilmington, MA, USA) to establish an anorectal fistula model. To create the model, a 1.3 mm diameter aluminum wire was wrapped 30 times with 2‐0 PERMA‐HAND silk suture (Ethicon). The wire's end was sharpened with pliers, and the wrapped silk was coated with freshly collected rat feces to inoculate the silk‐coated wire with bacteria. This wire was then inserted through the anus, piercing the rectal wall and traversing the outer skin, thus forming a tract connecting the skin to the rectum with an outer orifice ≈1 cm from the anus. The segment of the wire wrapped with the inoculated silk was placed inside the fistula tract to induce infection. To confirm fistula tract formation, subgroup rats were euthanized at 28 days, and the anorectal fistula region was harvested for ex vivo fluoroscopy (OEC Elite C‐Arm, GE Healthcare Systems), and micro‐CT (SkyScan 1276, Bruker) imaging. Fluoroscopy involved injecting Iohexol (OMNIPAQUE 350 mg mL^−1^, GE Healthcare, Chicago, IL, USA) into the fistula tract via a 5F catheter tip positioned in the outer orifice. To assess the host response to the hydrogels, the wire was removed at 7 days after fistula creation, and one of the hydrogel formulations (18NC75 or 18NC75‐10P‐1IL) was injected through the fistula's outer orifice using a 5F introducer catheter (Cook Medical) until the hydrogel completely filled the tract. In a separate set of rats, the fistula tract was created and occluded with 18NC75‐10P‐1IL hydrogels, but the outer orifice was closed using a 5‐0 polypropylene suture (PROLENETM, Ethicon) immediately after occlusion to prevent hydrogel extrusion. Subgroups of rats were euthanized at 0‐, 2‐, and 7‐days post‐injection, and the tissue harboring the fistula was harvested and fixed in 10% buffered formalin, then processed for ex vivo micro‐CT (Skyscan 1276) scanning and histological evaluation. Micro‐CT scanning parameters included: 50 Volts, 200 µA current, 0.4 angular rotation, 2 frame averaging, 180 degrees of axial rotation, and 20 µm voxel resolution. Hydrogel volumes were measured on reconstructed image stacks using CTAn software, applying standardized global threshold values for accurate segmentation from surrounding tissues.

### Histological Analysis

Formalin‐fixed tissue samples obtained from the rat subcutaneous injection model and the rat anorectal fistula model were embedded in paraffin blocks and serially sectioned at a thickness of 6 µm on positively charged histology slides (ThermoFisher). Histology sections from the rat subcutaneous injection model were stained with H&E (epredia, Kalamazoo, MI, USA), Masson's trichrome (Thermo Fisher Scientific), or immunostained for markers of proliferation and vascularization using anti‐PCNA IgG (1:250, ab92552, Abcam, Cambridge, MA, USA), and anti‐CD31 IgG (1:1000, ab182981, Abcam), respectively. Digital images were acquired using Evos FL Auto 2 (Thermo Fisher Scientific), and QuPath software was employed for morphometric analysis, including infiltrated cell count, the thickness of the zone with enhanced cellular infiltration surrounding the subcutaneously injected hydrogel, fibrous capsule thickness around the injected zone, PCNA^+^ cell count, and CD31^+^ cell count in 12 randomly selected fields surrounding the injected zone in four independent sections. Tissue sections from the rat anorectal fistula model were stained with H&E (epredia), Masson's trichrome (Thermo Fisher Scientific), and the Gram Brown‐Brenn modified stain (Newcomer Supply, WI, USA) to visualize tissue morphology and the presence of gram‐negative or gram‐positive bacteria, respectively. Immunohistochemistry was additionally performed to assess proliferation, vascularization, neutrophil, and macrophage cell infiltration using IgGs specific for detecting PCNA (1:250, ab92552, Abcam), CD31 (1:1000, ab182981, Abcam), Myeloperoxidase (MPO, 1:500, ab208670, Abcam), CD68 (1:250, ab125212, Abcam). Anti‐iNOS IgG (1:500, ab283655, Abcam) was used to identify pro‐inflammatory macrophages. Microscopic images were acquired using the Evos FL Auto 2 microscope (Thermo Fisher Scientific). Morphometric analyses of PCNA^+^ cell count, CD31^+^ cell count, MPO^+^ cell count, CD68^+^ cell count, and iNOS^+^ cell count were performed using QuPath software in 12 randomly selected fields from three independent tissue sections.

### Complete Blood Count (CBC) and Blood Chemistry

For CBC analysis, rat whole blood was collected from the vein into a BD Vacutainer EDTA tube (BD) and analyzed with Veterinary hematology analyzer (Heska, Loveland, CO, USA). For serum collection, rat whole blood was collected in a Vacuette CAT serum clot activator tube (Greiner Bio‐One, Kremsmünster, Austria) and centrifuged at 2000 × g for 10 min. Serum chemistry was analyzed using Veterinary Chemistry Analyzer (DRI‐CHEM 4000, Heska) and serum cytokines/chemokines levels were analyzed using Rat Cytokine/Chemokine Multiplex Discovery Assay Array (RD27) (Eve Technologies, Calgary, CA, USA).

### Bacterial Identification

Seven days after creating the anorectal fistula model, pus was collected from the outer orifices of four rats using a sterile cotton‐tipped swab and cultured in LB broth overnight. The cultured bacteria were plated onto Columbia agar with 5% sheep blood (Becton Dickinson, Sparks, MD, USA) using a standard 4‐quadrant streak for isolation. Isolated colonies exhibiting unique morphologies were identified using the FDA‐approved Biotyper MALDI‐TOF mass spectrometry system (Bruker Scientific, Billerica, MA, USA) following the manufacturer's protocol. Briefly, individual colonies were spotted via stick onto a stainless‐steel target and overlayed with the Biotyper matrix (Bruker Scientific). The target was processed on the Biotyper Sirius using the Clinical Application software with a library extension of up to claim 6.

### Antimicrobial Test with Patient‐Derived Bacteria

A 500 µL aliquot of 18NC75‐10P‐1IL was dispensed into a well of a 12‐well tissue culture plate (Celltreat) and centrifuged to ensure uniform distribution. Following this, four distinct patient‐derived pathogens (*S. haemolyticus, S. epidermidis, MRSA*, and *E. cloacae*) were inoculated either onto the hydrogels or into empty wells designated as the control. The inoculation was carried out at a density of 7.5 × 10^7^ CFU in 500 µL of normal saline. The setup underwent overnight incubation at 37 °C in a humidified incubator. Post‐incubation, an additional 500 µL of saline was introduced into each well to resuspend the pathogens, and the resultant suspension was collected by pipetting. Subsequently, 100 µL from each collected sample was plated on a blood agar plate (TSA w/ sheep blood, Remel, Lenexa, KS, USA) and incubated overnight at 37 °C. The plates were observed and photographed to assess bacterial growth.

### Statistical Analysis

Statistical analyses were performed using GraphPad Prism 9 software. Data were presented as mean ± standard error of the mean (s.e.m.). Statistical significance was assessed using analysis of variance (ANOVA) with Tukey's multiple‐comparison post hoc test for comparisons involving three or more groups, and unpaired Student's t‐test for comparisons between two independent groups. The p values of <0.05 were considered statistically significant. The significance levels were indicated as follows: *p < 0.05, **p < 0.01, ***p < 0.001, and ****p < 0.0001. ns indicates no significance.

## Conflict of Interest

The authors declare no conflict of interest.

## Author Contributions

R.O. conceived the idea. R.O. and J.K. designed the experiments. J.K. and H.K. performed and J.K. and R.O. analyzed in vitro experiments. Z.Z., J.K., and H.A. created the rat subcutaneous implantation model and anorectal fistula model and performed the procedural experiments; J.K. and R.O. analyzed the data. J.K. and E.G. performed the antimicrobial tests. J.M. created 3D‐rendered models of subcutaneously injected hydrogels and created 3D‐printed fistula‐mimicking models. R.O. supervised the work and is the principal investigator of the supporting grants.

## Supporting information



Supporting Information

## Data Availability

The data that support the findings of this study are available from the corresponding author upon reasonable request.
