# Supplementary material for: Catheter Injectable Multifunctional Biomaterial for the Treatment of Infected Enterocutaneous Fistulas
Source: Adv Sci (Weinh). 2025 Feb 14;12(20):2414642. doi: 10.1002/advs.202414642 (PMC12120755; doi:10.1002/advs.202414642)
Supplement: Supplementary file 1 — Supporting Information [file ADVS-12-2414642-s001.docx]

Supporting Information

**Catheter Injectable Multifunctional Biomaterial for the Treatment of Infected Enterocutaneous Fistulas**

*Jinjoo Kim, Zefu Zhang, Hassan Albadawi, Hyeongseop Keum, Joseph L. Mayer, Erin H. Graf, and Rahmi Oklu**


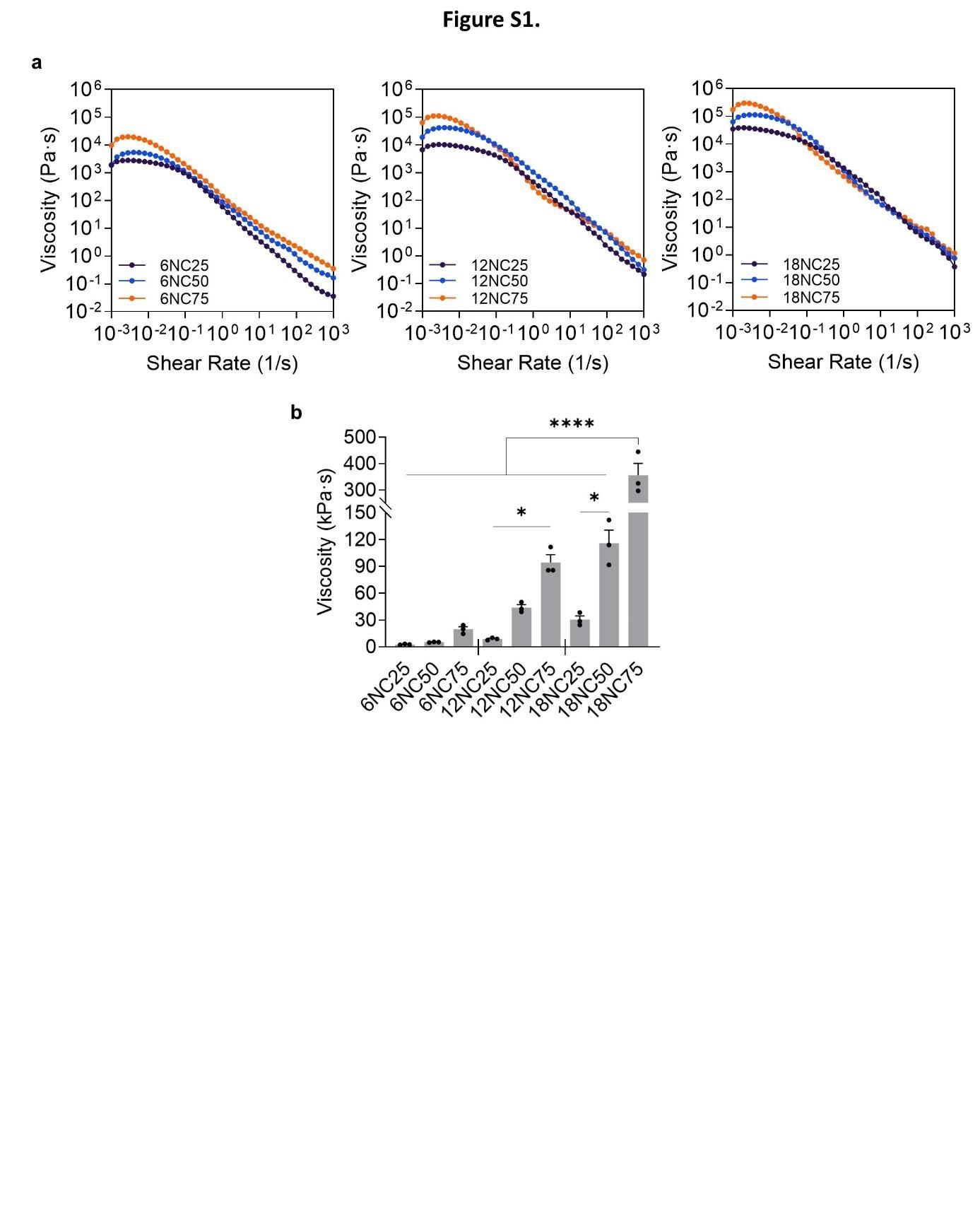


**Figure S1. Viscosity measurements of gelatin and NS nanocomposite hydrogels. a,** Rheology studies showing the viscosity flow curves of various gelatin and NS nanocomposite formulations were generated across a shear rate range of 10^-3^ to 10^3^ s^-1^. **b,** Graph depicting the maximum viscosity values of various gelatin and NS nanocomposite formulations (n=3). Data are mean ± s.e.m. Statistical significance was determined by one-way ANOVA with Tukey’s multiple-comparison test. ns, not significant, *p < 0.05, **p < 0.01, ***p < 0.001, ****p < 0.0001.


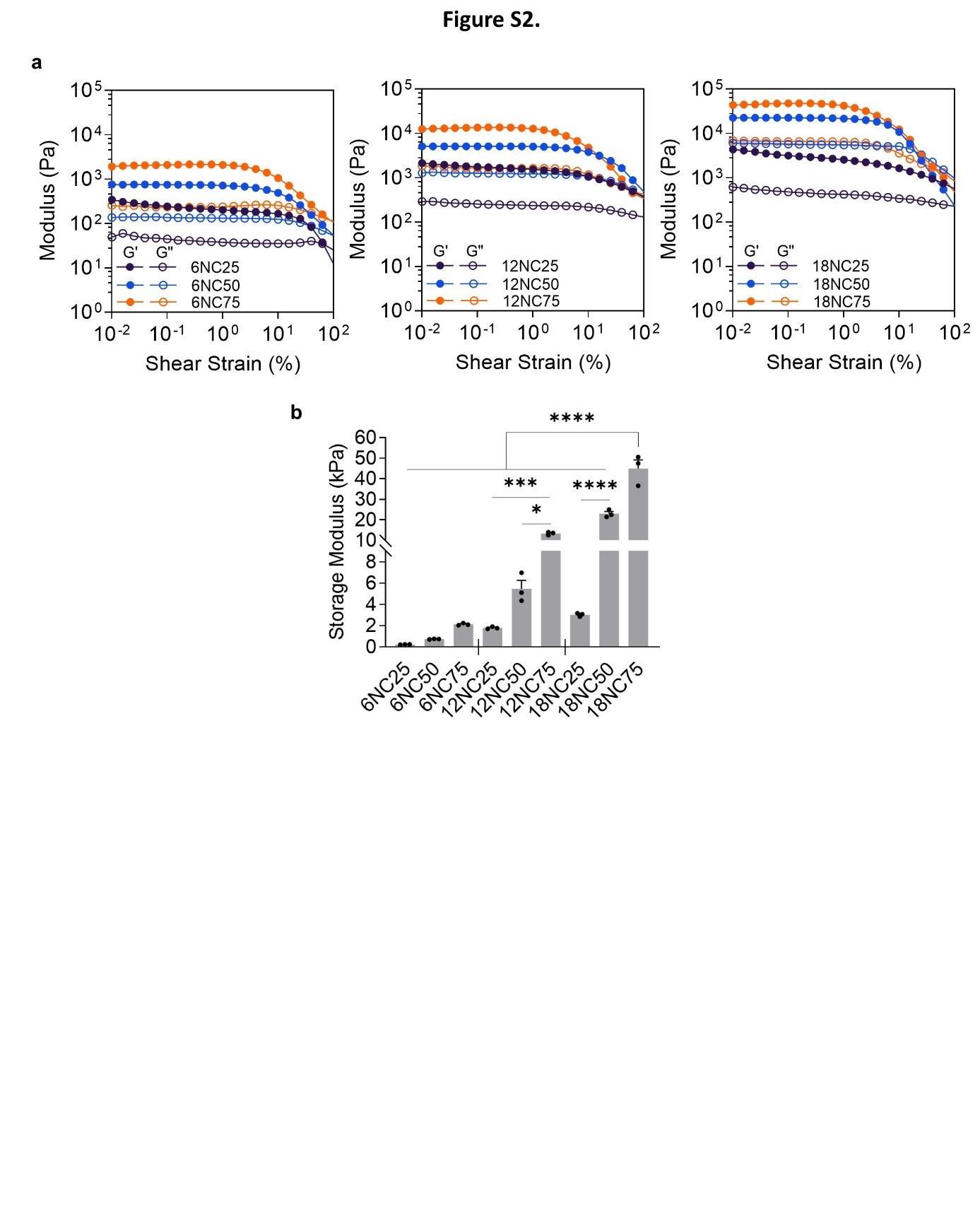


**Figure S2. Modulus measurements of gelatin and NS nanocomposite hydrogels. a,** Representative curves of oscillatory strain sweeps showing storage (G’) and loss (G’’) modulus of various gelatin and NS nanocomposite formulations measured at a constant angular frequency of 10 rad s^-1^ and shear strain range from 10^-2^ to 10^2^%. **b,** Graph illustrating the average storage modulus generated by various gelatin and NS nanocomposite formulations at shear strain of 10^-1^% (n=3). Data are mean ± s.e.m.; statistical significance was determined by one-way ANOVA with Tukey’s multiple-comparison test. ns, not significant, *p < 0.05, **p < 0.01, ***p < 0.001, ****p < 0.0001.


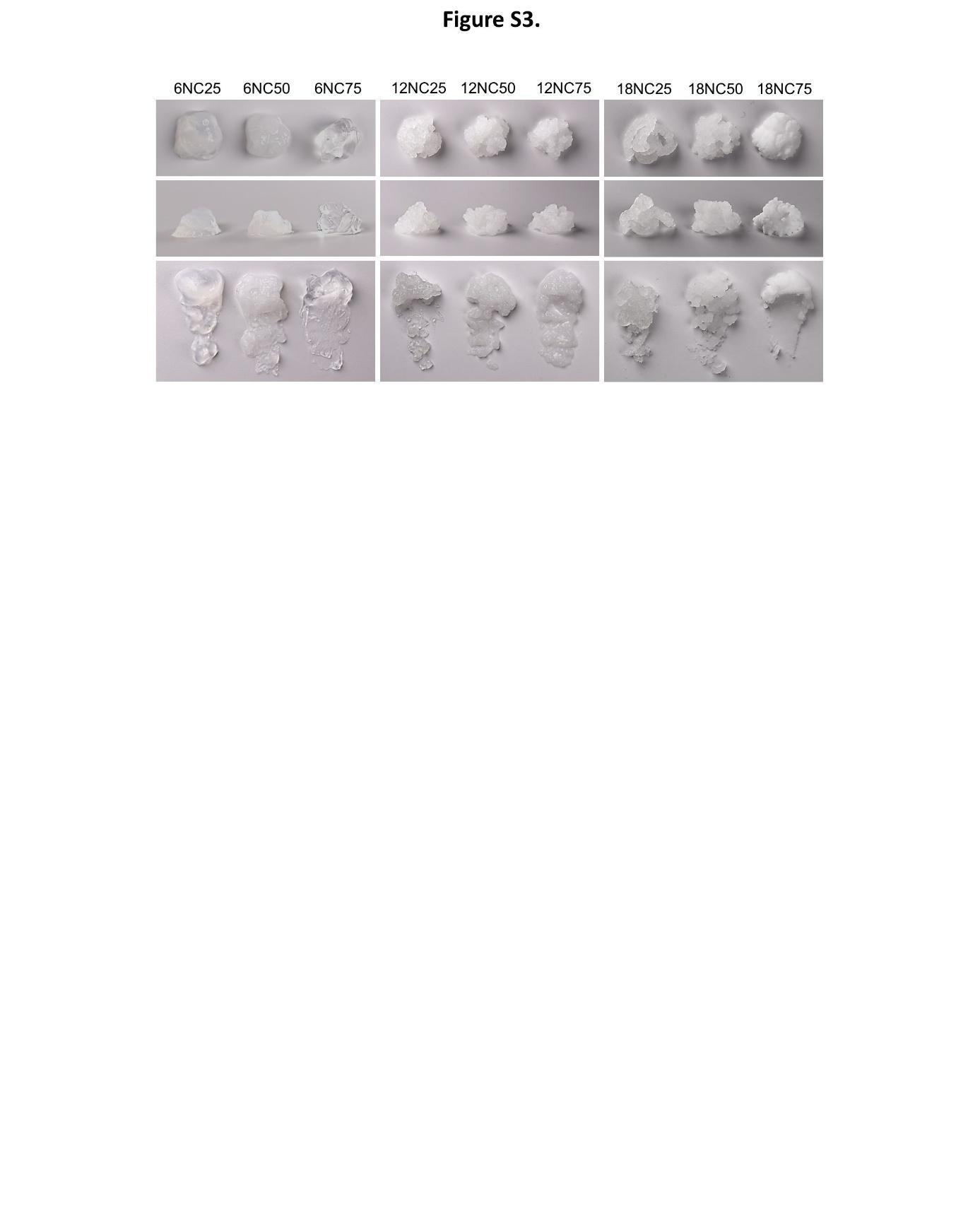


**Figure S3.** Photographs of various formulations of gelatin and NS nanocomposite hydrogels depicting visual material differences.


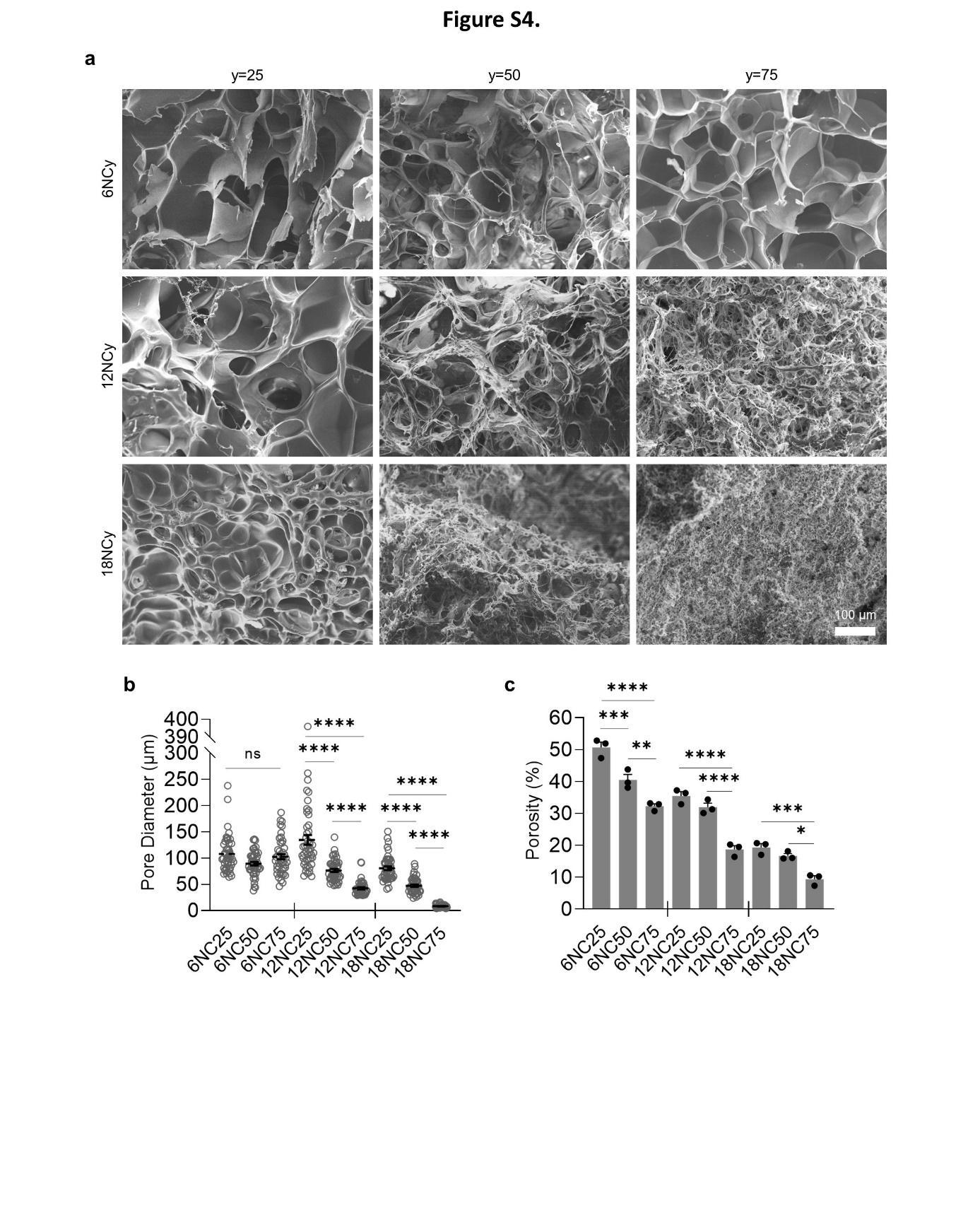


**Figure S4. Scanning electron microscopy (SEM) analysis of xNCy. a,** Representative SEM images of varying gelatin + NS nanocomposite formulations (xNCy) containing three different concentrations of total solids (NS + gelatin; x = 6, 12, and 18% w/w) and/or three different percentages of NS in the total solid mass (y = 25, 50, and 75% w/w) showing a decrease in pore size as the total solid mass and the total NS concentration increases. **b,** Graph depicting the measurement of pore diameter measured in SEM images of varying gelatin + NS nanocomposite formulations (n=46). **c,** Graph demonstrating the measured porosity in SEM images of varying gelatin + NS nanocomposite formulations (n=3). Data are mean ± s.e.m.; statistical significance was determined by one-way ANOVA with Tukey’s multiple-comparison test. ns, not significant, *p < 0.05, **p < 0.01, ***p < 0.001, ****p < 0.0001.


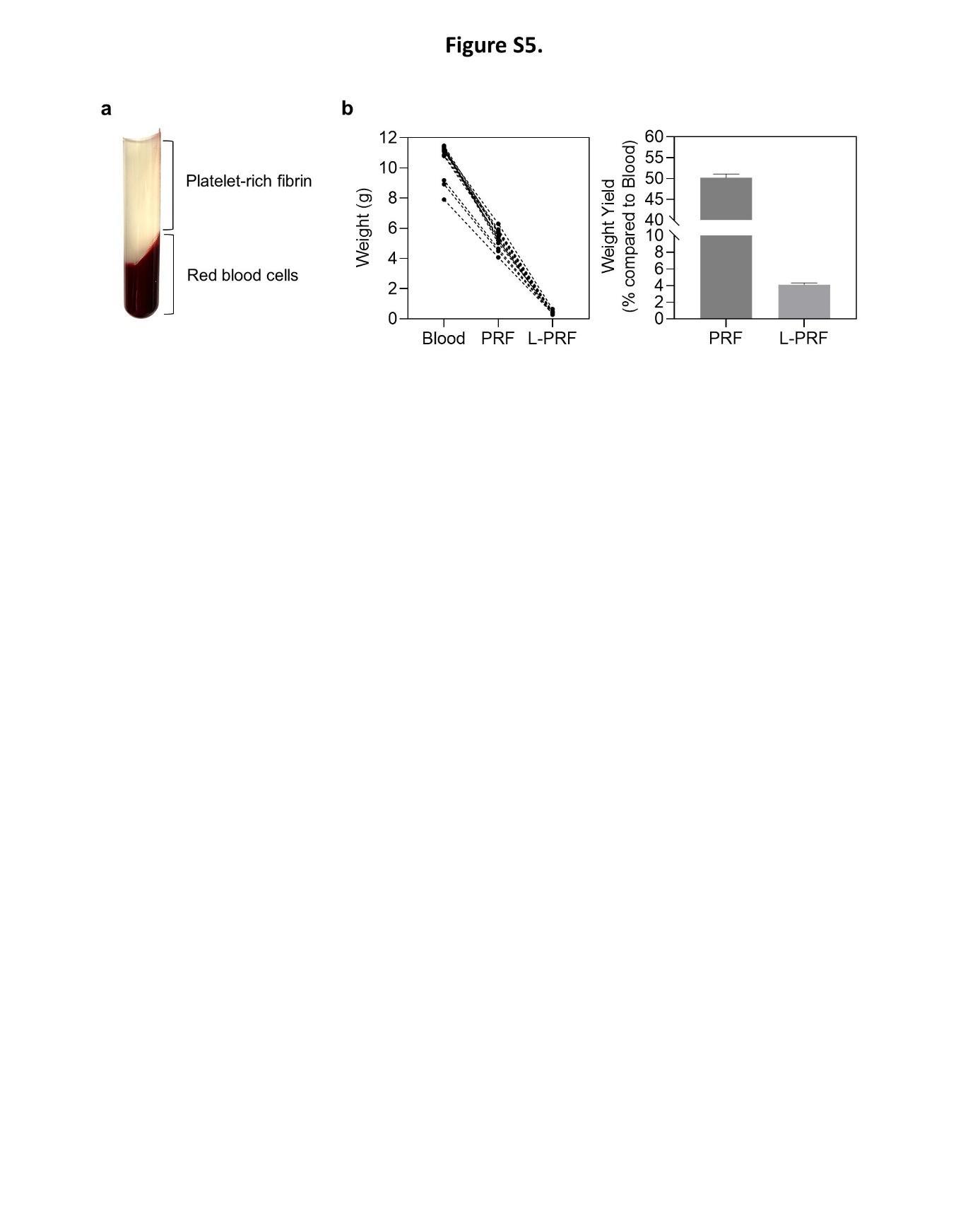


**Figure S5. Preparation of lyophilized PRF (L-PRF). a,** Photograph of whole porcine blood after centrifugation in a glass tube showing phase separation of red blood cells (lower) layer and a platelet-rich fibrin (PRF, upper) layer. **b,** Plot and a bar graph demonstrating the weight of whole porcine blood aliquot compared to the weight of collected PRF and the L-PRF, and the percentage of weight yield of PRF and L-PRF compared to the weight of the original whole blood aliquots (n=14). Data are mean ± s.e.m.


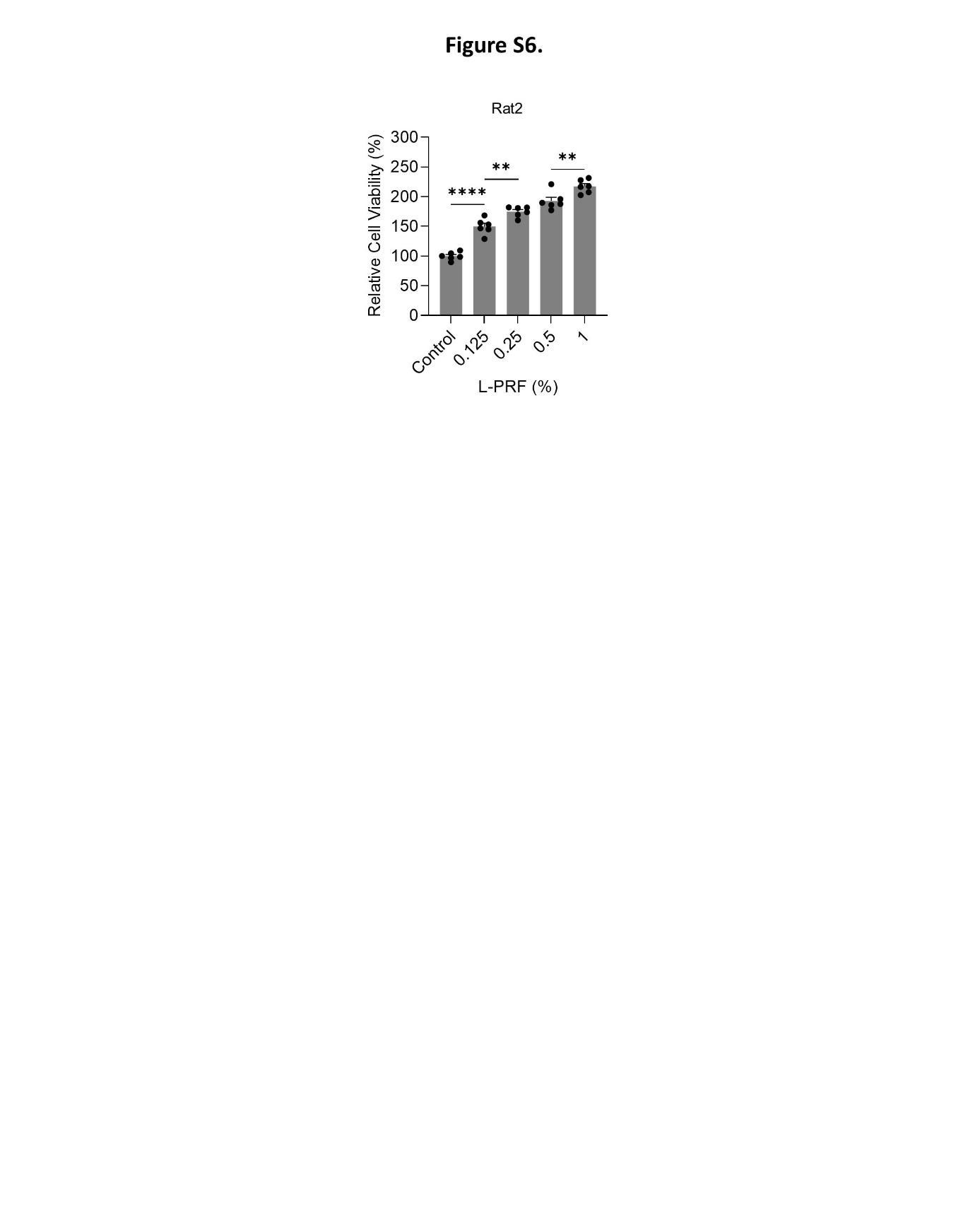


**Figure S6. Assessing the effect of L-PRF treatment on Rat2 cell proliferation.** Relative cell viability of Rat2 cells treated with varying concentrations of L-PRF (0, 0.125, 0.25, 0.5, and 1 w/v%) showing a concentration-dependent enhanced proliferation at 24 h after L-PRF treatment (n=6). Data are mean ± s.e.m. Statistical significance was determined by one-way ANOVA with Tukey’s multiple-comparison tests. ns, not significant, *p < 0.05, **p < 0.01, ***p < 0.001, ****p < 0.0001.


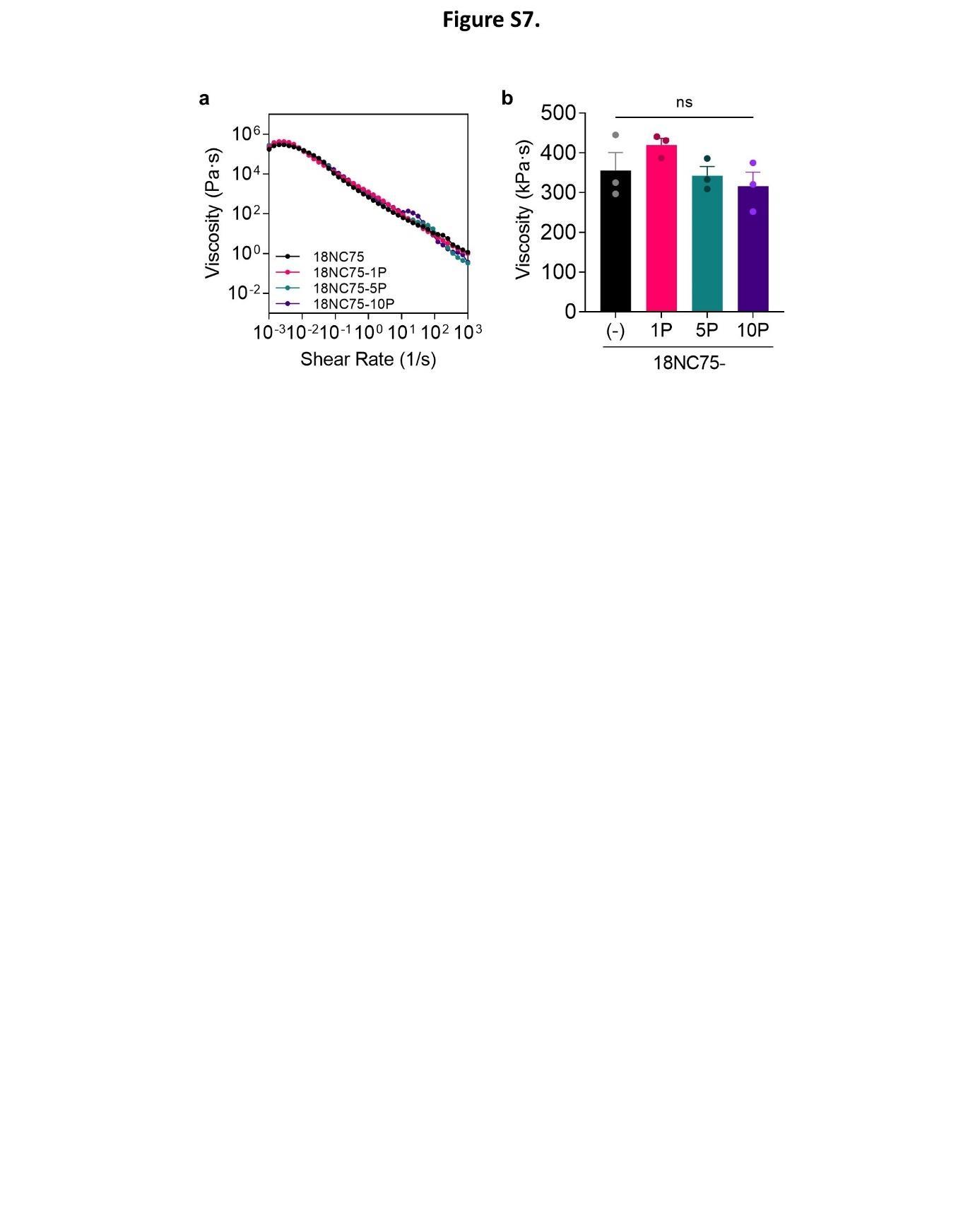


**Figure S7. Evaluating the viscosity of 18NC75 containing varying L-PRF ratios. a,** Representative viscosity curves of 18NC75, or 18NC75 containing varying L-PRF ratios of 1, 5, and 10 (w/w)%, respectively (18NC75-1P, 18NC75-5P, and 18NC75-10P) generated across a shear rate range of 10^-3^ to 10^3^ s^-1^. **b,** Graph showing the average of maximum viscosity measured with 18NC75, 18NC75-1P, 18NC75-5P, and 18NC75-10P (n=3). Data are mean ± s.e.m.; statistical significance was determined by one-way ANOVA with Tukey’s multiple-comparison tests. ns, not significant, *p < 0.05, **p < 0.01, ***p < 0.001, ****p < 0.0001.


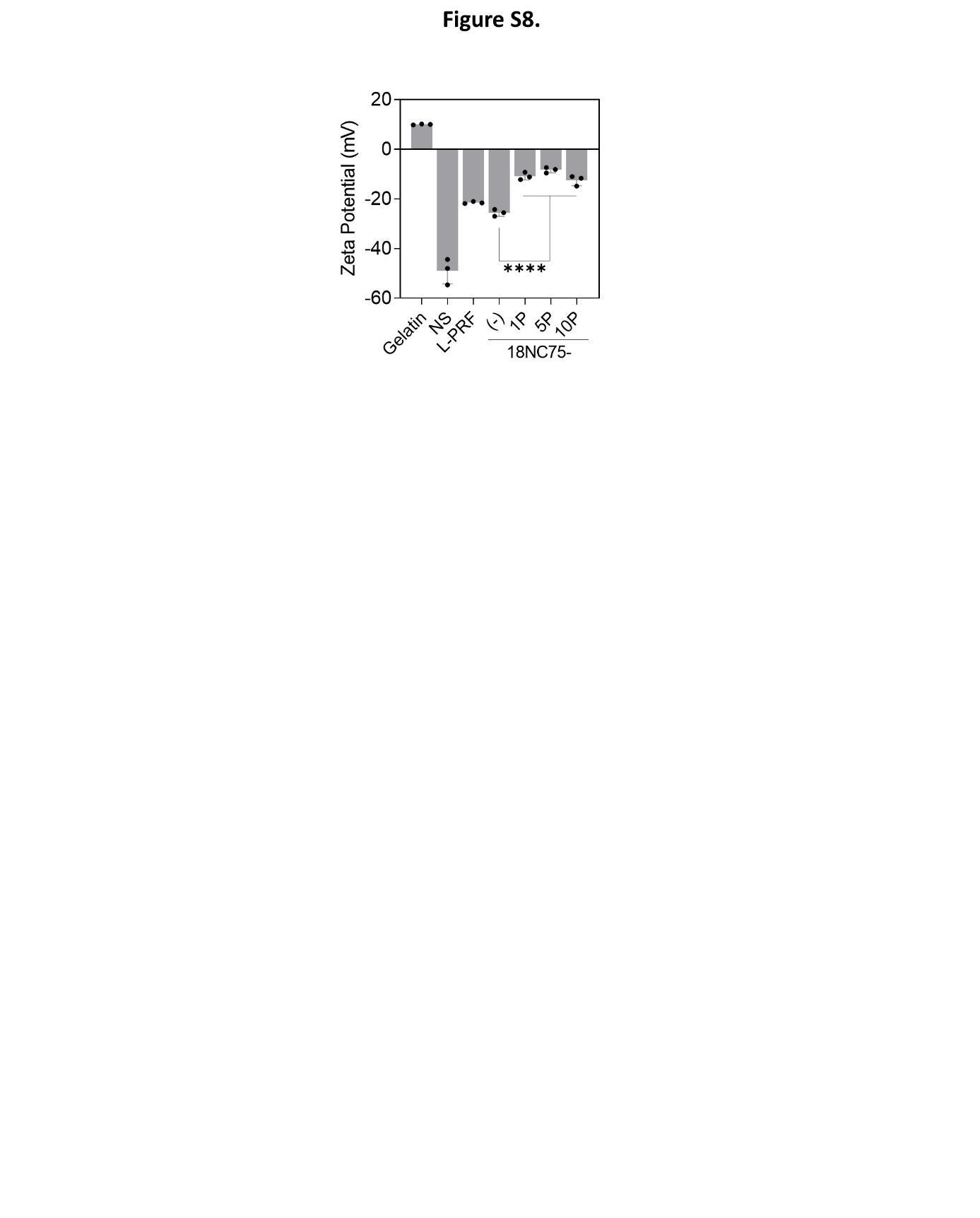


**Figure S8.** Plot depicting the zeta potential of gelatin (4.5% w/w), NS (13.5% w/w), L-PRF (10% w/w), 18NC75, 18NC75-1P, 18NC75-5P, and 18NC75-10P demonstrating a marked increase in zeta potential after the incorporation of L-PRF in 18NC75 hydrogels (n=3). Data are mean ± s.e.m.; statistical significance was determined by one-way ANOVA with Tukey’s multiple-comparison test. ****p < 0.0001.


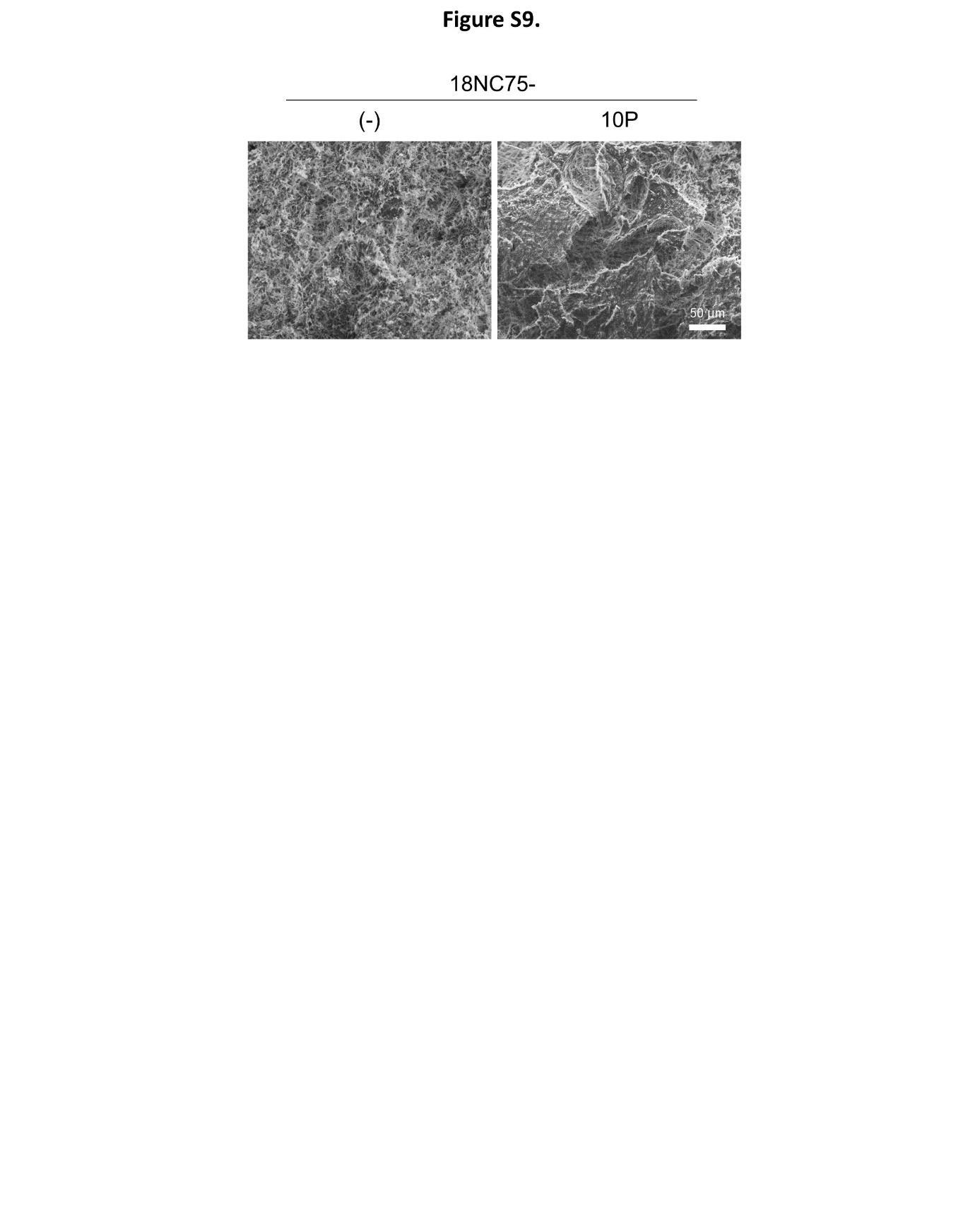


**Figure S9.** Representative SEM images of 18NC75 and 18NC75-10P showing the decrease in porosity after adding L-PRF to 18NC75.


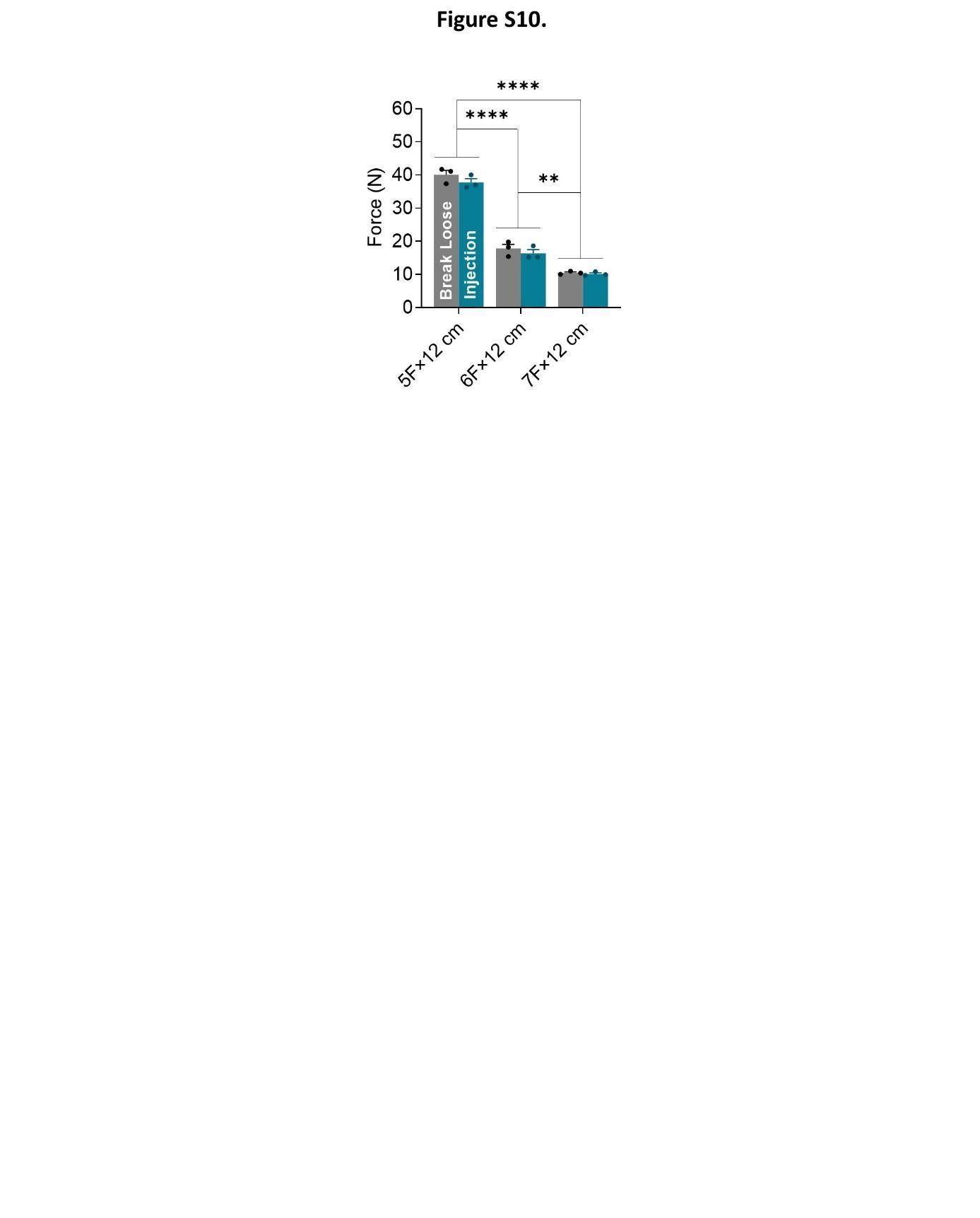


**Figure S10.** Graph depicting the break-loose and the injection forces generated by 18NC75-10P aliquots loaded in 1 mL syringes and injected through various catheters. Data are mean ± s.e.m.; statistical significance was determined by two-way ANOVA with Tukey’s multiple-comparison test. ns, not significant, *p < 0.05, **p < 0.01, ***p < 0.001, ****p < 0.0001.


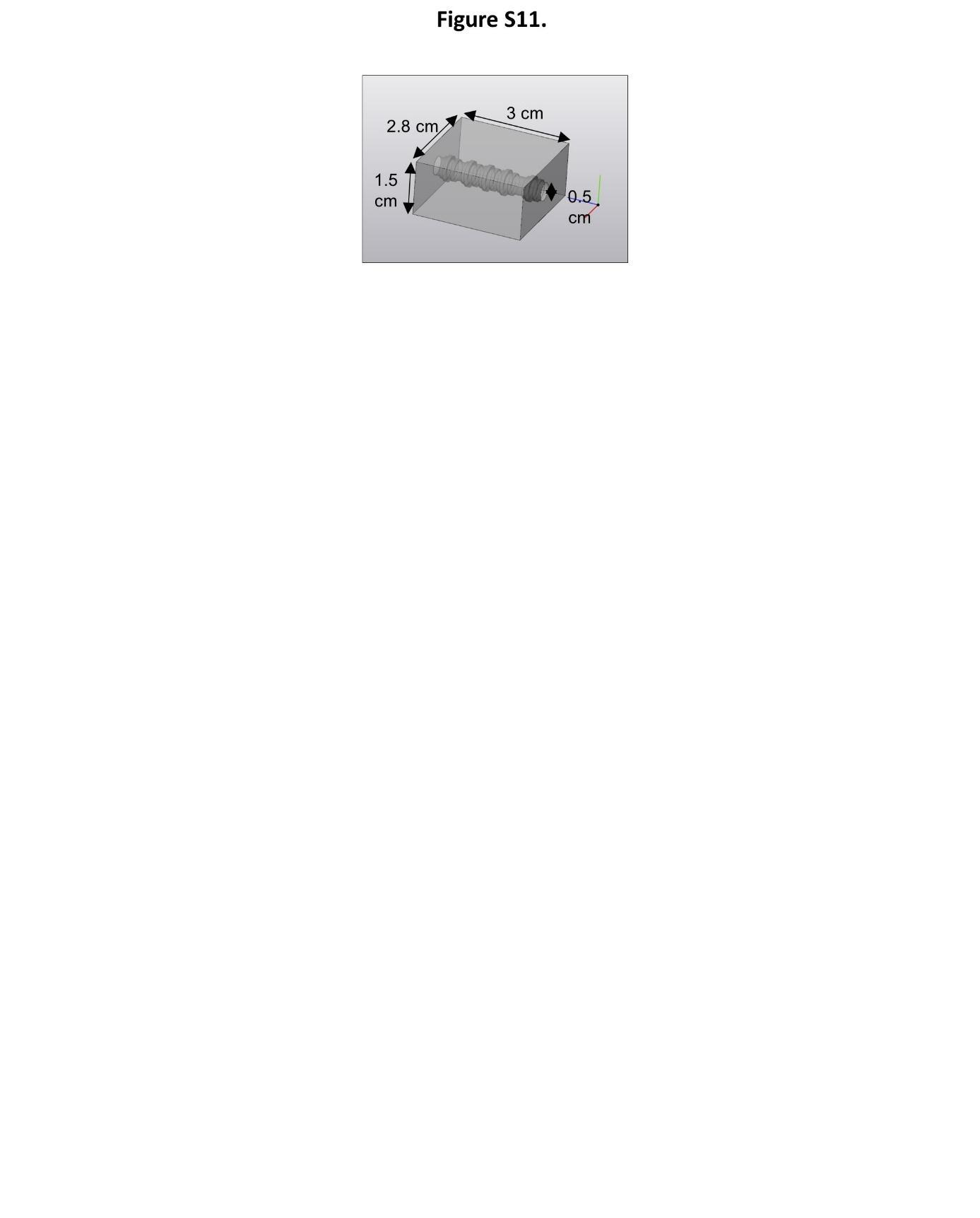


**Figure S11.** A schematic of the 3D-printed fistula model designed for the perpendicular displacement pressure testing of the hydrogels after loading into the fistula tract measuring 3 cm in length.


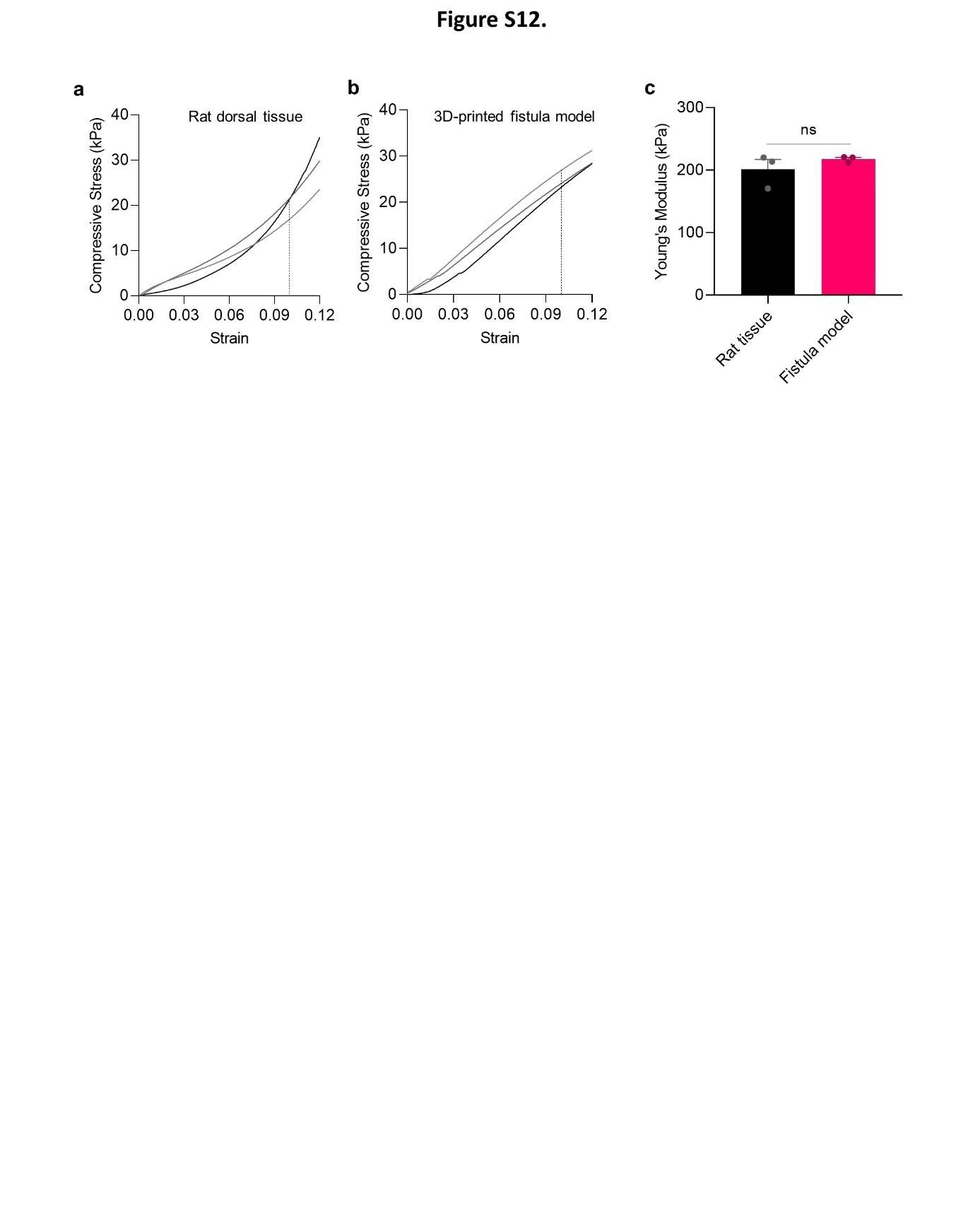


**Figure S12.** Compressive strain-stress curves for rat dorsal tissues (a) or for the 3D-printed fistula model (b), and the corresponding Young’s modulus generated by each test (c), showing the similarity in the stiffness between the fistula model and the rat dorsal tissues (n=3). Data are mean ± s.e.m. statistical significance was determined by unpaired Student’s t-test. ns, not significant, *p < 0.05, **p < 0.01, ***p < 0.001, ****p < 0.0001.


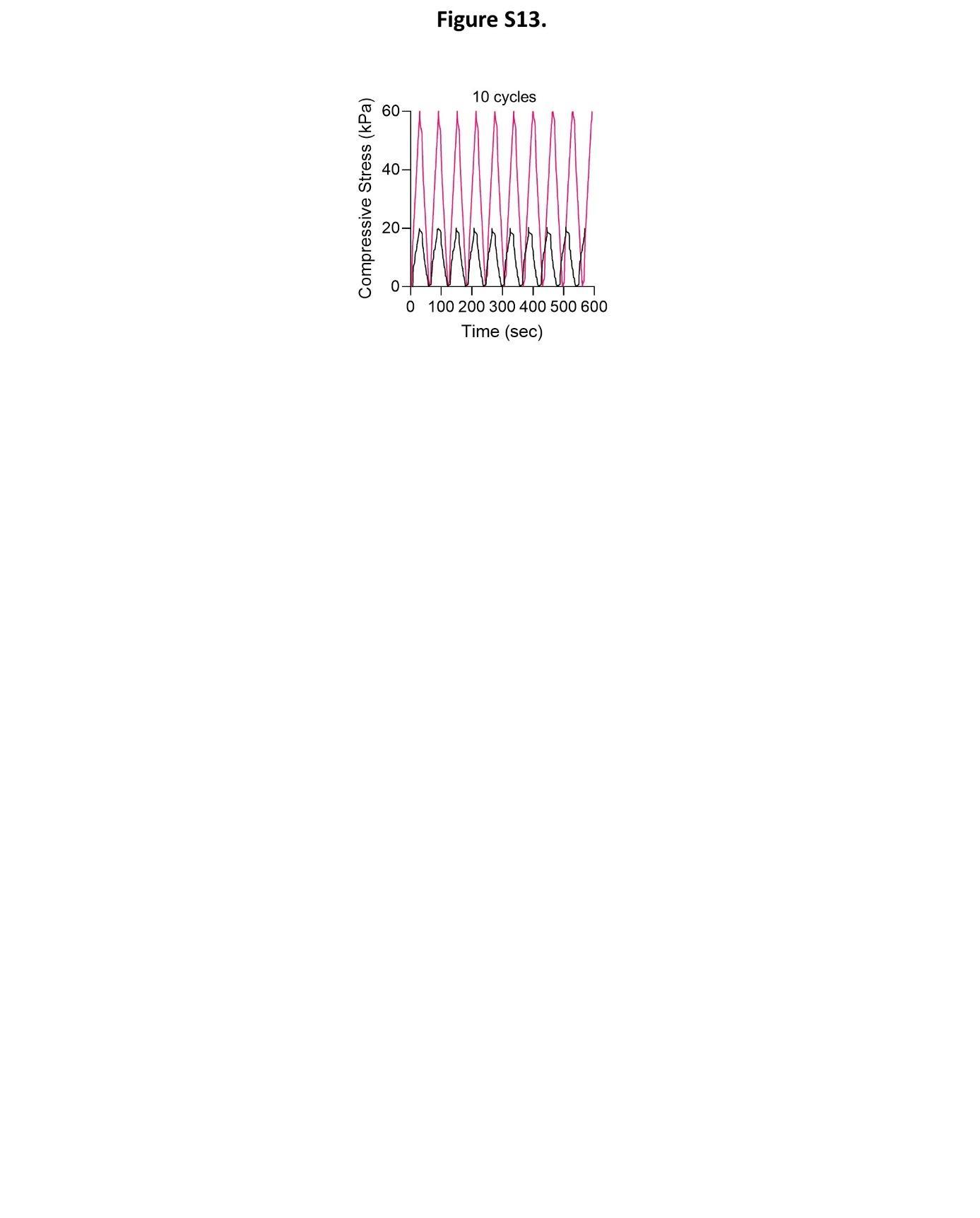


**Figure S13.** Plot illustrating the cyclic compressive pressure applied during perpendicular displacement pressure testing of the fistula-mimicking model. The pressure oscillates between 0 - 20 or 0 - 60 kPa, repeated for 10 cycles in each test.


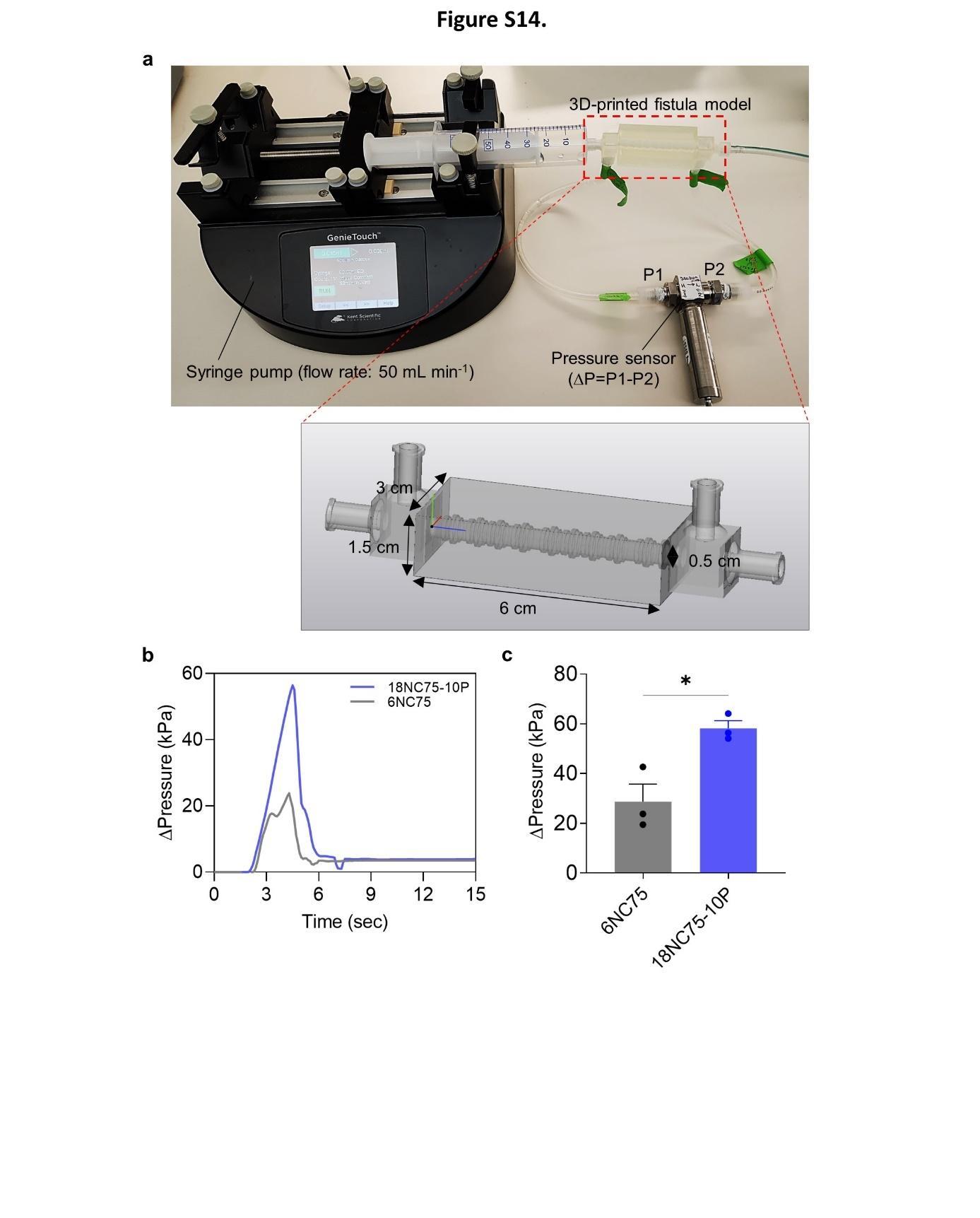


**Figure S14. Axial displacement pressure test. a,** Experimental setup of axial displacement pressure test and a schematic of 3D-printed fistula-mimicking model. **b,** Representative time-dependent curves of axial pressure required to displace 6NC75 and 18NC75-10P from the 3D-printed fistula-mimicking model. **c,** Graph depicting the average maximum pressure required to displace 6NC75 and 18NC75-10P from the 3D-printed fistula-mimicking model (n=3). Data are mean ± s.e.m.; statistical significance was determined by unpaired Student’s t-test. ns, not significant,*p < 0.05, **p < 0.01, ***p < 0.001, ****p < 0.0001.


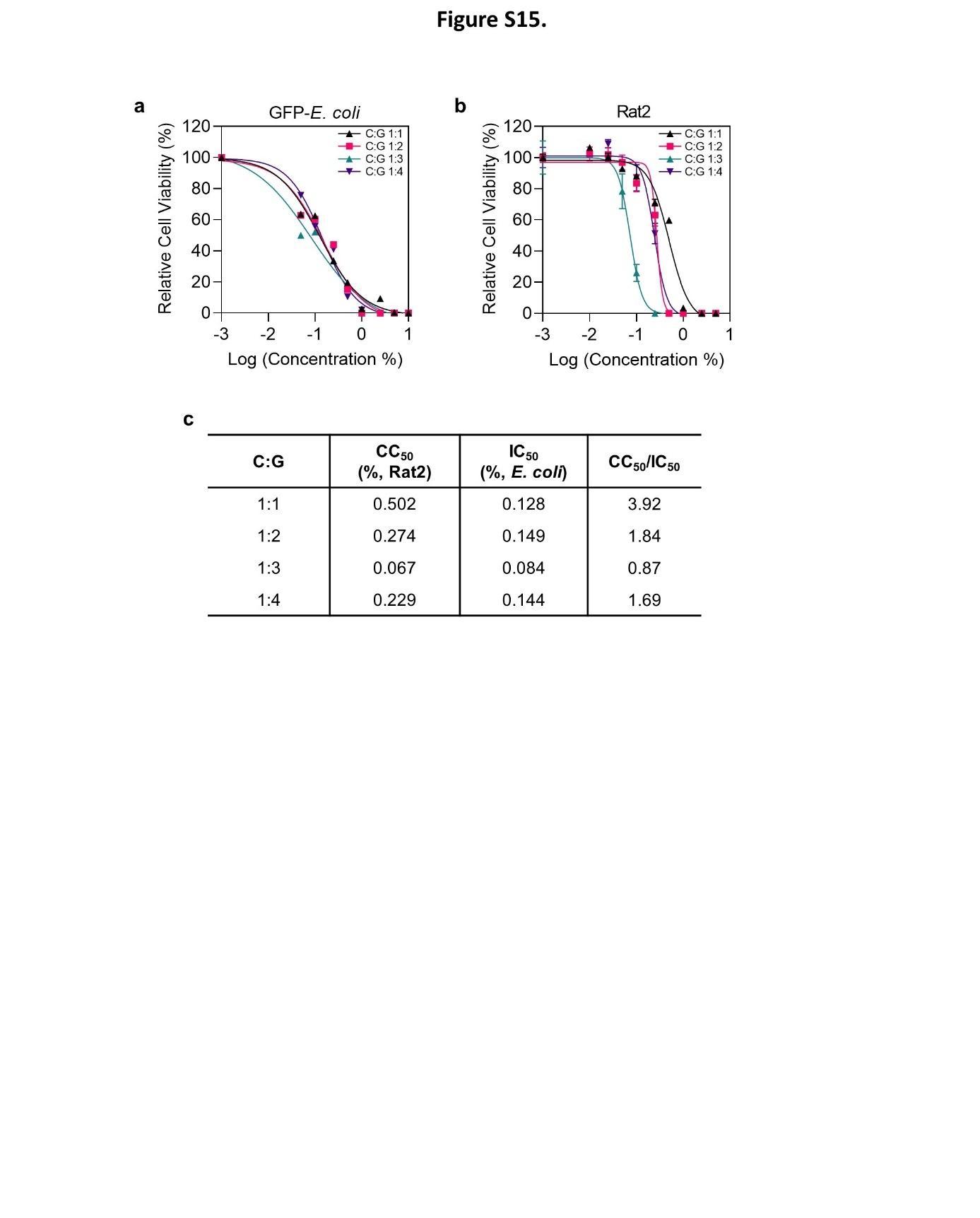


**Figure S15. *In vitro* optimization of IL’s C:G molar ratio for effective antimicrobial property. a, b,** Relative cell viability curves of GFP-*E. coli* (a) (n=5) and Rat2 cells (b) (n=6) treated with serially diluted ILs with varying Choline : Geranate molar ratios (C:G 1:1, C:G 1:2, C:G 1:3, and C:G 1:4). **c,** Table showing the CC_50_ values (50% cytotoxicity concentration) measured in Rat2 cells, and the IC50 values (50% inhibitory concentration) measure in *E. coli*, and CC_50_ to IC_50_ ratio (CC_50_/IC_50_) used for the selectivity index of Ils with varying molar ratios. Data are mean ± s.e.m.


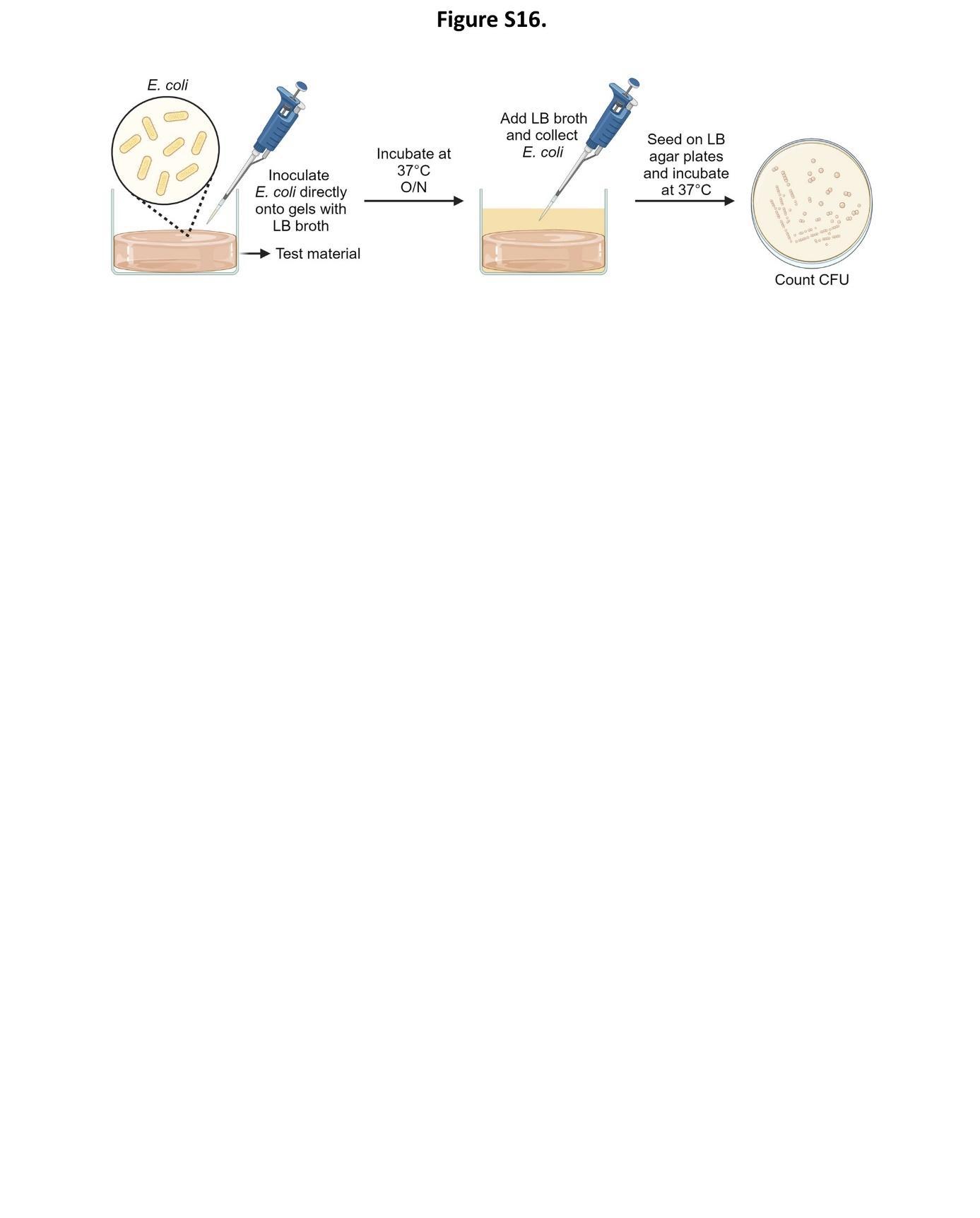


**Figure S16.** Schematic illustrating the experimental method of contact-mode antimicrobial efficacy test with *E. coli*.


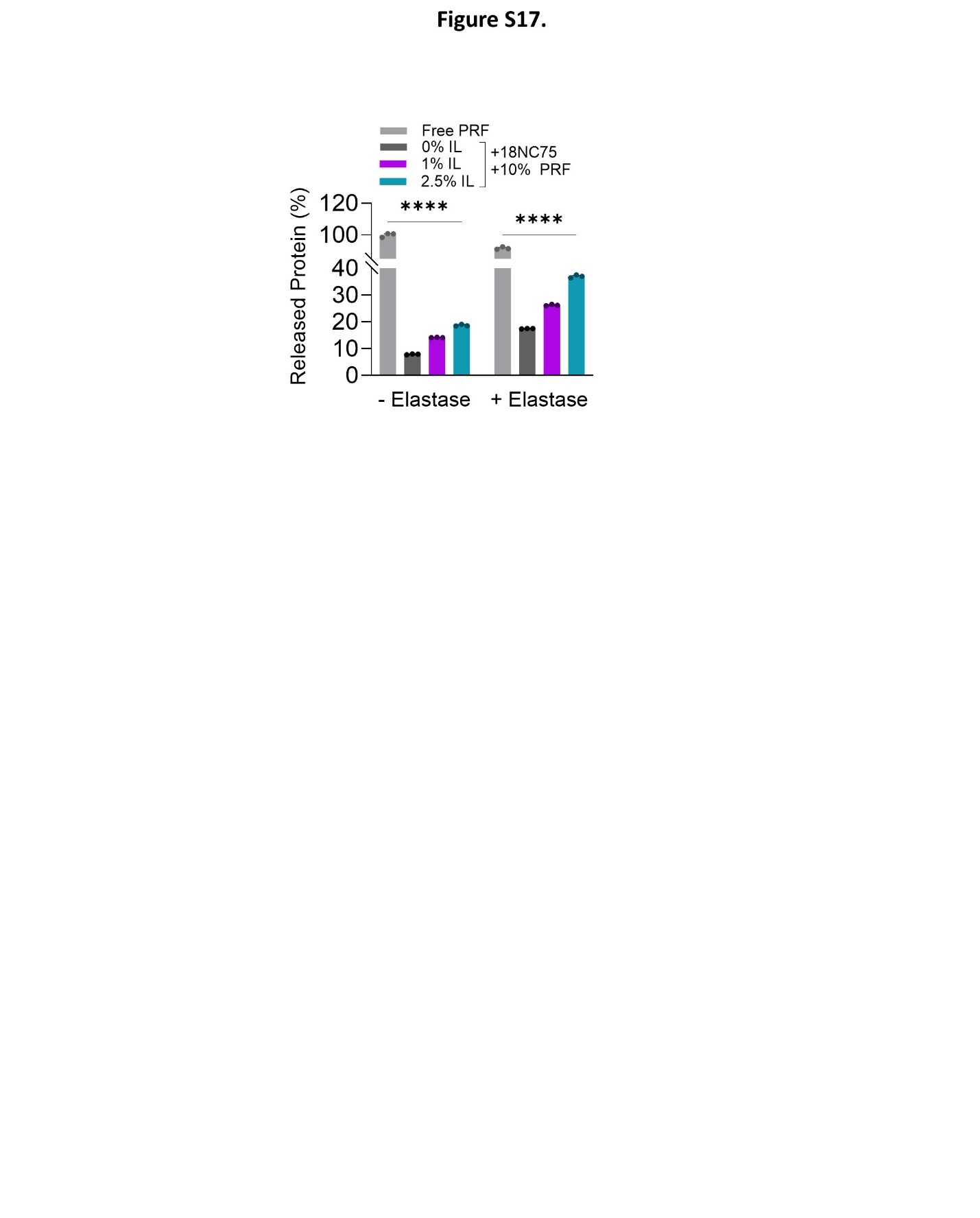


**Figure S17.** Graph depicting the percentage of released proteins from L-PRF, 18NC75-10P, 18NC75-10P-1IL, and 18NC75-10P-2.5IL, all containing the same amount of L-PRF, after 24 h of incubation at 37°C with or without Elastase (n=3). Data are presented as mean ± s.e.m. Statistical significance was determined by two-way ANOVA with Tukey’s multiple-comparison test. ns, not significant; ****p < 0.0001.


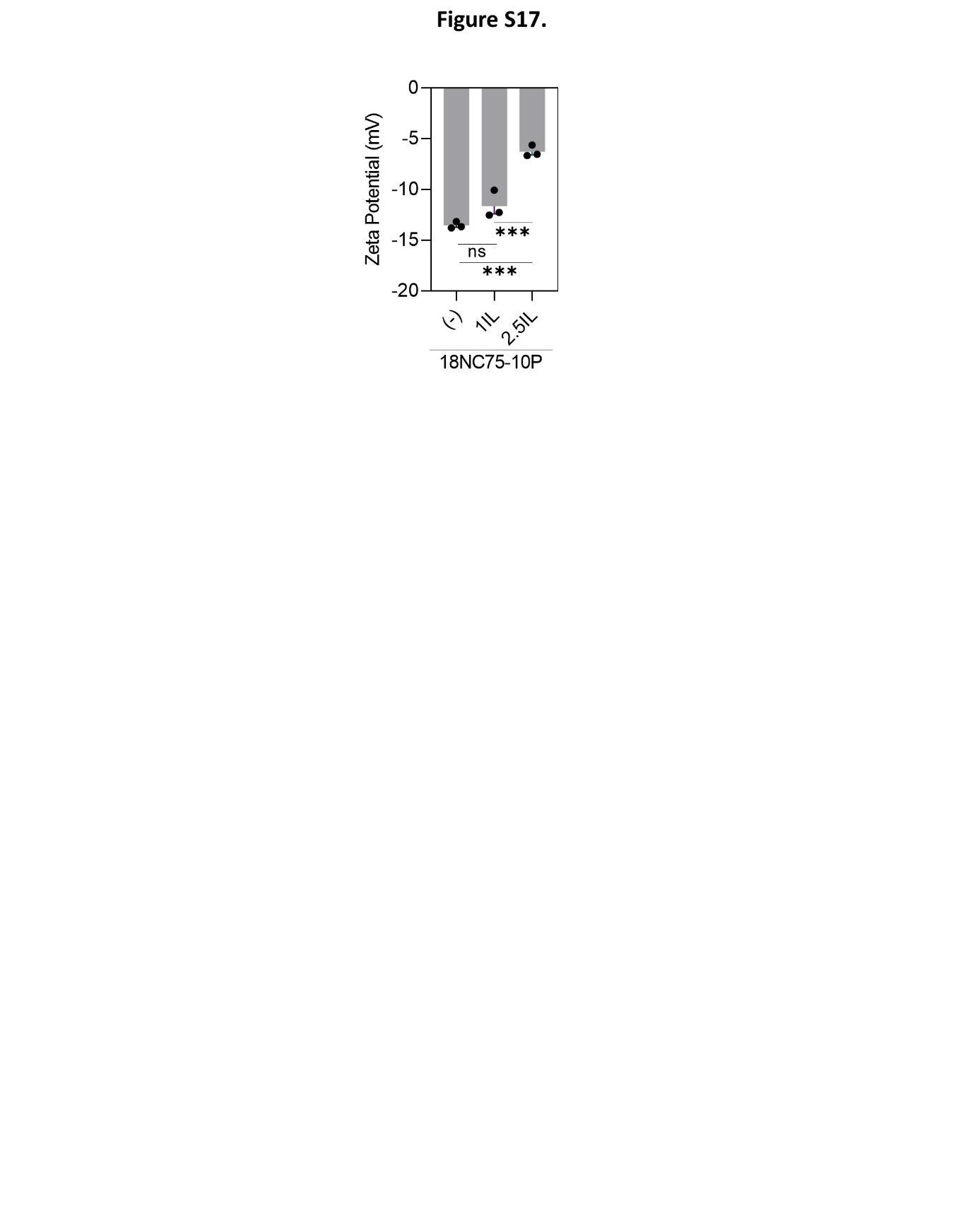


**Figure S18.** Graph showing the zeta potential of 18NC75-10P, 18NC75-10P-1IL, and 18NC75-10P-2.5IL (n=3). Data are mean ± s.e.m.; statistical significance was determined by one-way ANOVA with Tukey’s multiple-comparison test. ns, not significant,*p < 0.05, **p < 0.01, ***p < 0.001, ****p < 0.0001.


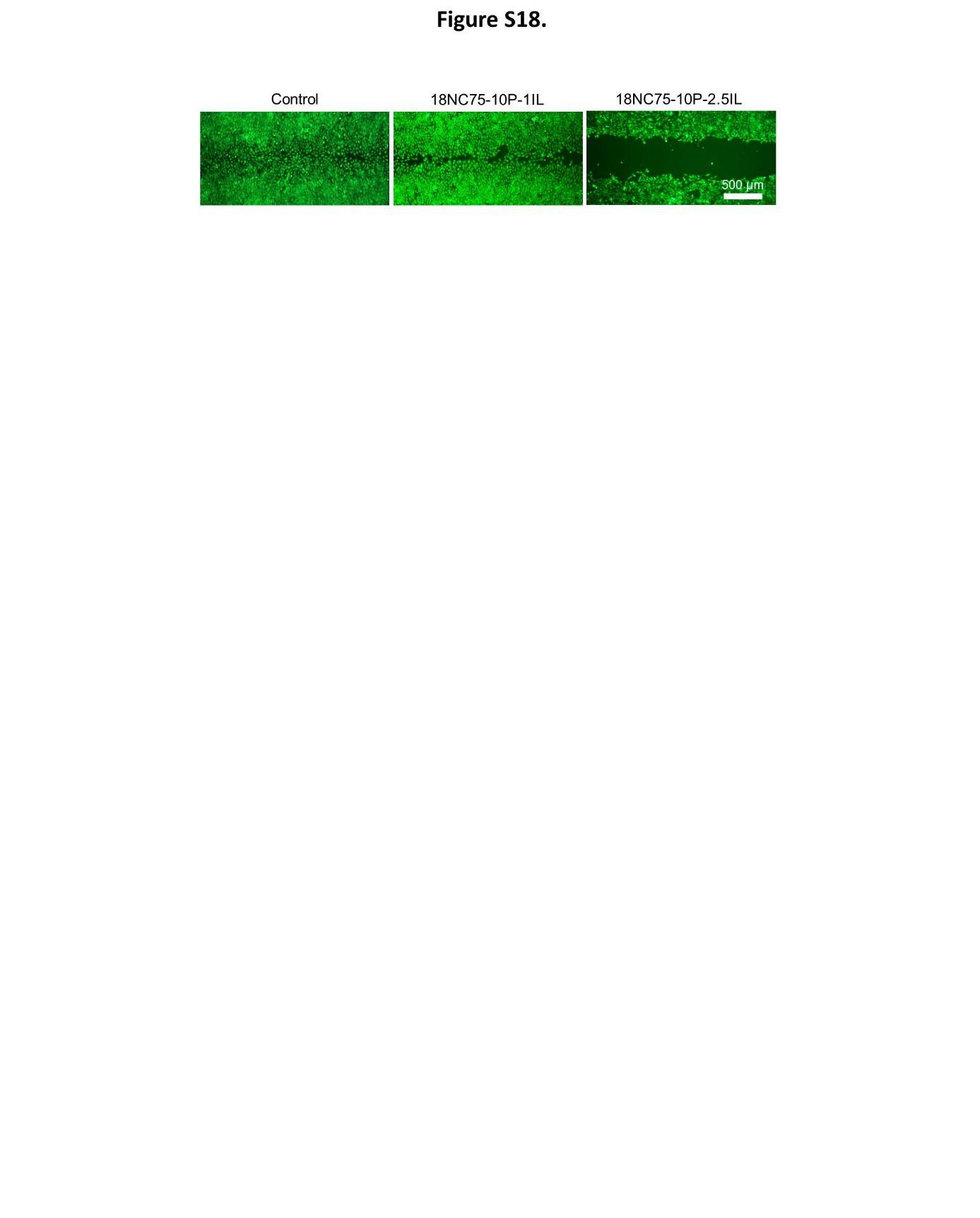


**Figure S19.** Representative fluorescence images of cell migration test with Rat2 cells incubated with growth medium (control) or growth medium containing leachables from 18NC75-10P-1IL and 18NC75-10P-2.5IL for 24 h.


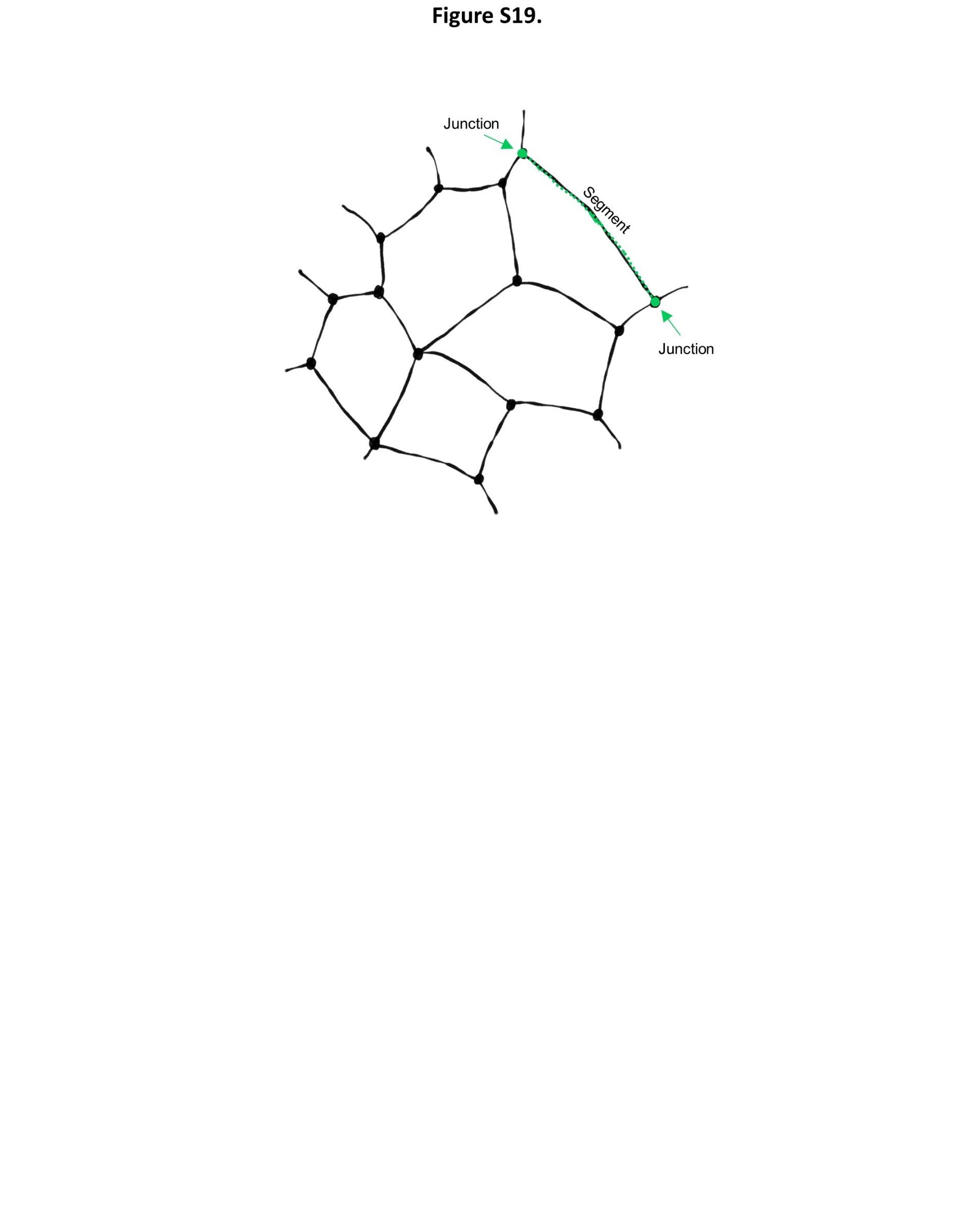


**Figure S20.** Schematic depicting Junction and Segment in the analysis of tube formation assay.


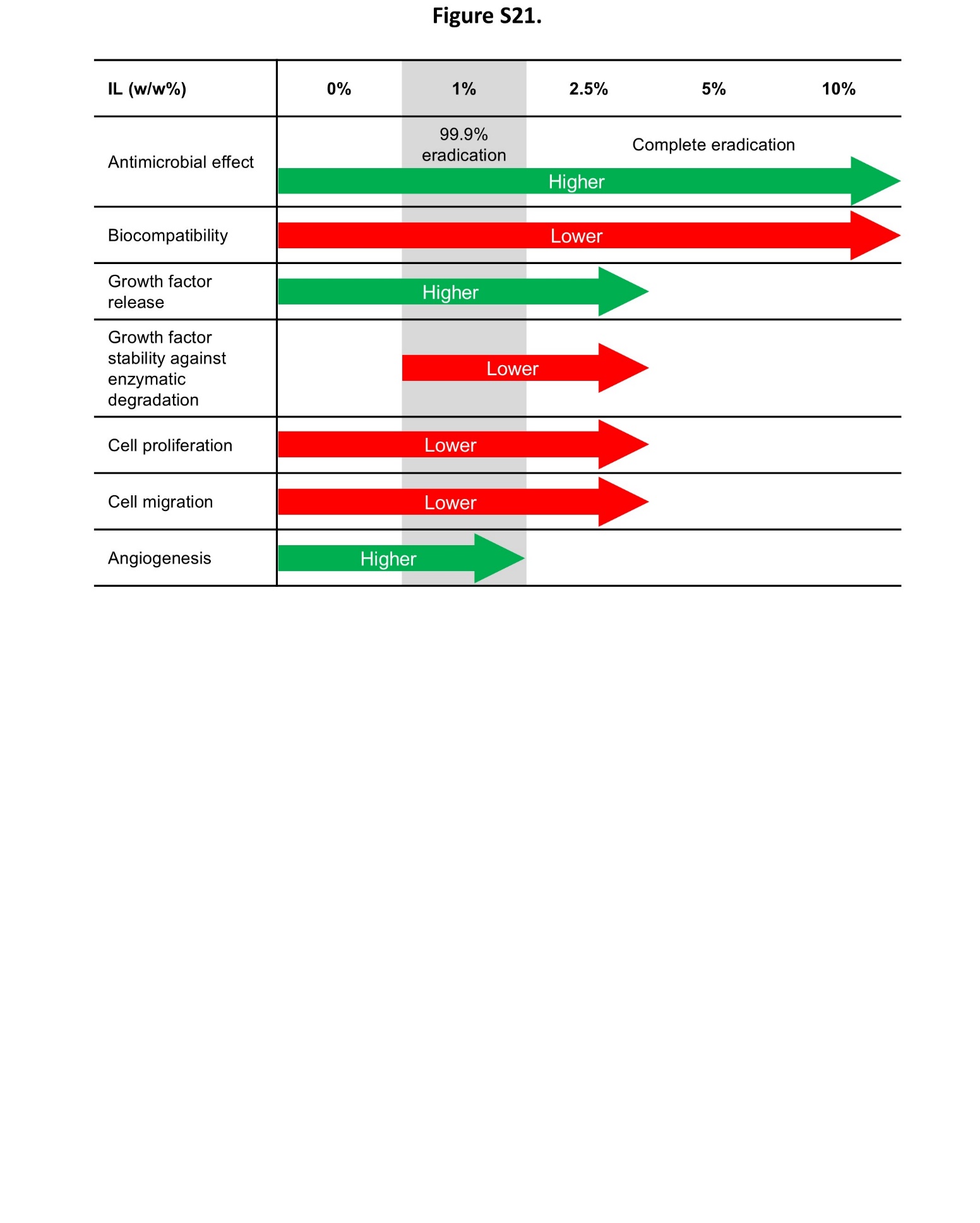


**Figure S21.** Rationale for selecting the optimal IL concentration in the 18NC75-10P hydrogel formulation.


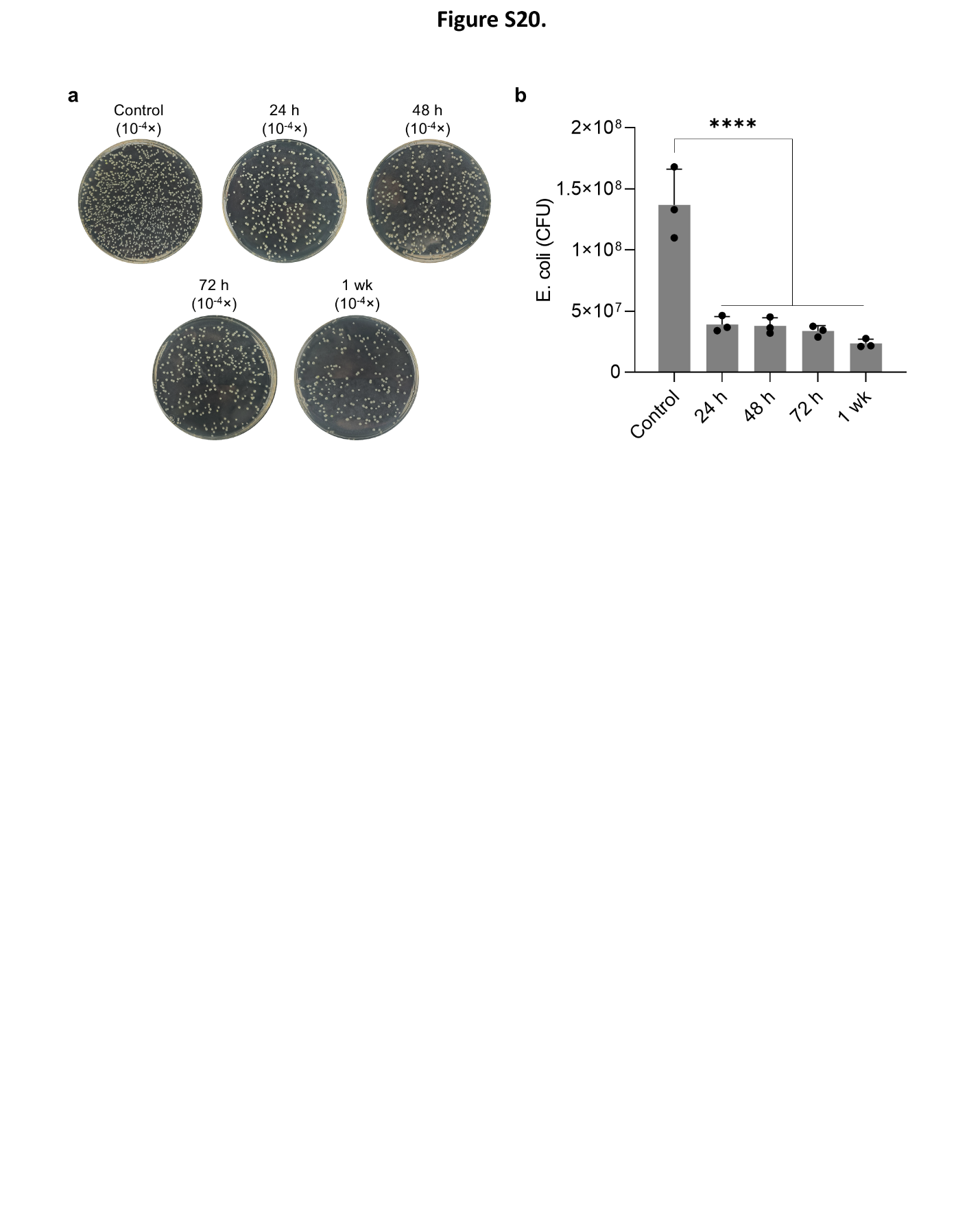
**Figure S22.** **Bactericidal effect of 18NC75-10P-1IL leachables.** **a,** Images of LB agar plates demonstrating the bactericidal effect of 18NC75-10P-1IL leachables collected at various time points (24 h, 48 h, 72 h, and 1 wk). After incubation with each leachables, *E. coli* cultures were diluted at 10^-4^ before inoculation onto LB agar plates. **b,** The graph presents the average colony forming units (CFU) counted (n=3). Data are mean ± s.e.m.; statistical significance was determined by one-way ANOVA with Tukey’s multiple-comparison test. ****p < 0.0001.


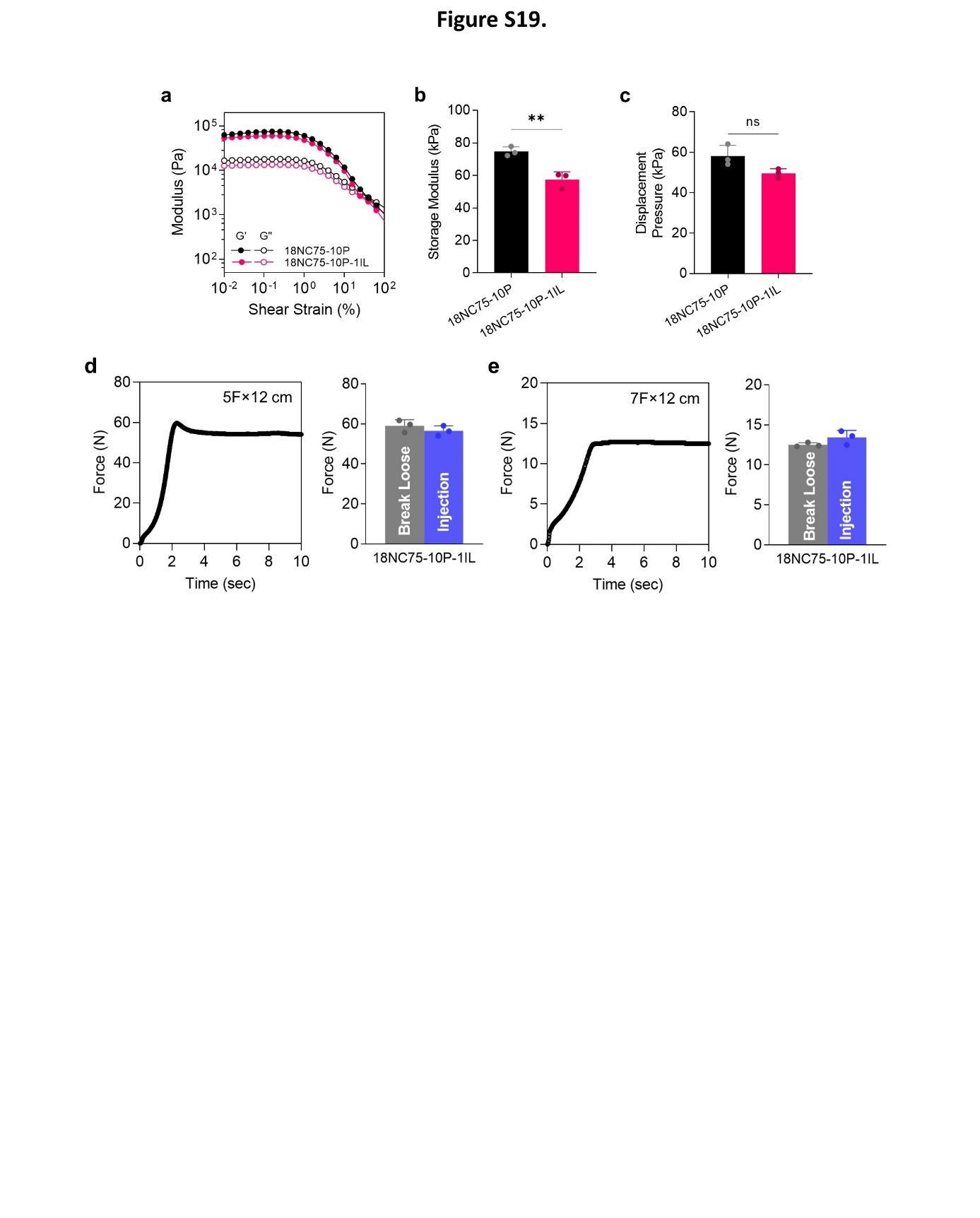


**Figure S23. Mechanical characterization of 18NC75-10P-1IL. a,** Representative curves of oscillatory strain sweeps depicting storage (G’) and loss (G’’) modulus of 18NC75-10P and 18NC75-10P-1IL under a constant angular frequency of 10 rad s^-1^ and shear strain range from 10^-2^ to 10^2^%. **b,** Average storage modulus of 18NC75-10P and 18NC75-10P-1IL at shear strain of 10-^1^% (n=3). **c,** The average maximum pressure required to displace 18NC75-10P and 18NC75-10P-1IL from the fistula-mimicking 3D-printed model (n=3). **d, e,** Representative time-dependent injection force flow curves and graphs illustrating the break loose and injection forces generated during the injection of a 1 mL aliquot of 18NC75-10P-1IL in a syringe through 5F or 7F catheters with a length of 12 cm, respectively (n=3 for each test). Data are mean ± s.e.m.; statistical significance was determined by unpaired Student’s t-test. ns, not significant,*p < 0.05, **p < 0.01, ***p < 0.001, ****p < 0.0001.


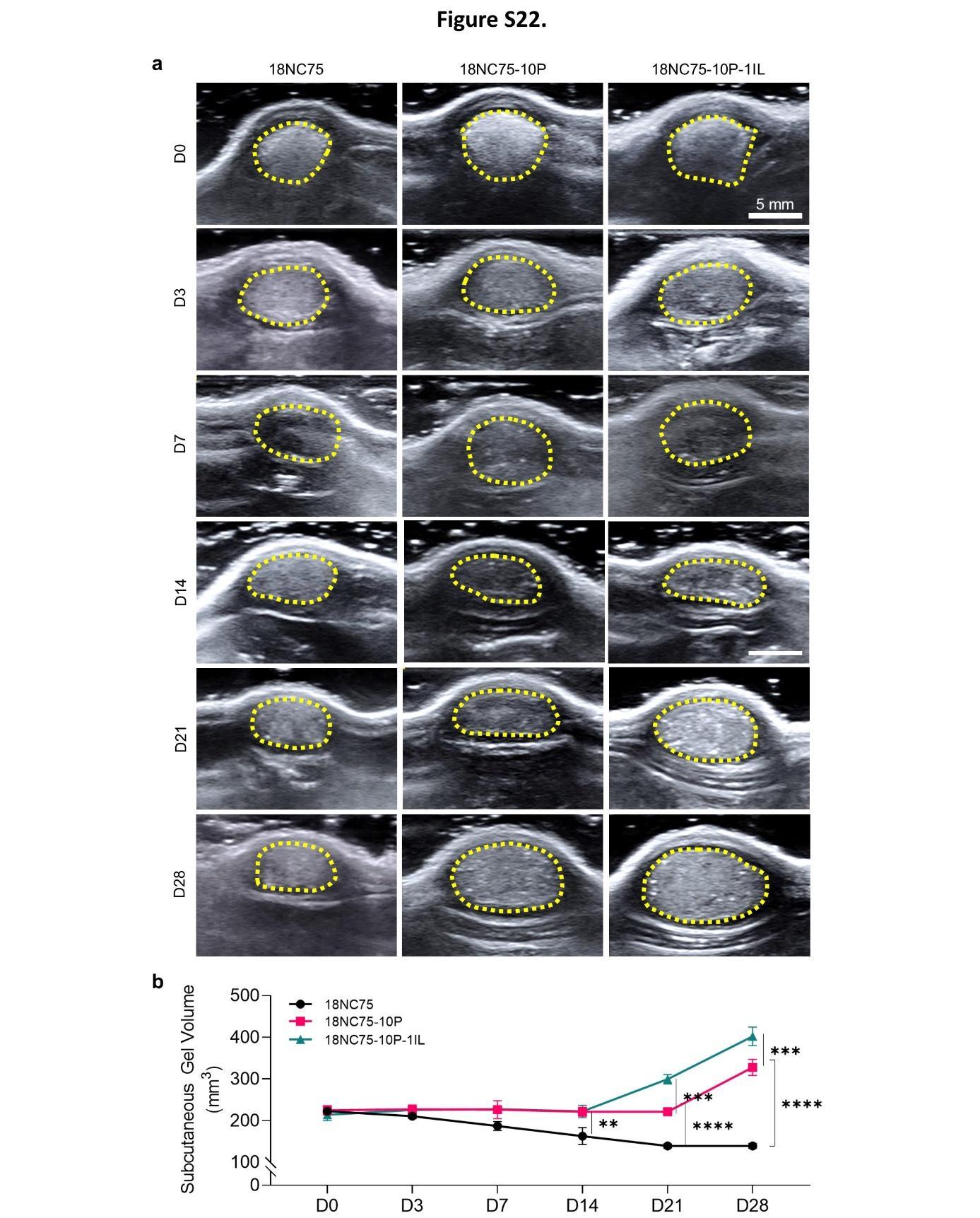


**Figure S24. Ultrasound imaging of subcutaneously injected hydrogels in rats. a,** Representative ultrasound images of rats' dorsum subcutaneously injected with 18NC75, 18NC75-10P, and 18NC75-10P-1IL at D0, D3, D7, D14, D21, and D28 post-injection. Yellow dotted lines indicate injected materials. **b,** Plot illustrating the time-dependent change in injected material volume for 18NC75, 18NC75-10P, and 18NC75-10P-1IL assessed at D0, D3, D7, D14, D21, and D28 post-injection using ultrasound imaging (n=8). Data are presented as mean ± s.e.m.; statistical significance was determined by two-way ANOVA with Tukey’s multiple-comparison tests. **p < 0.01, ***p < 0.001, ****p < 0.0001.


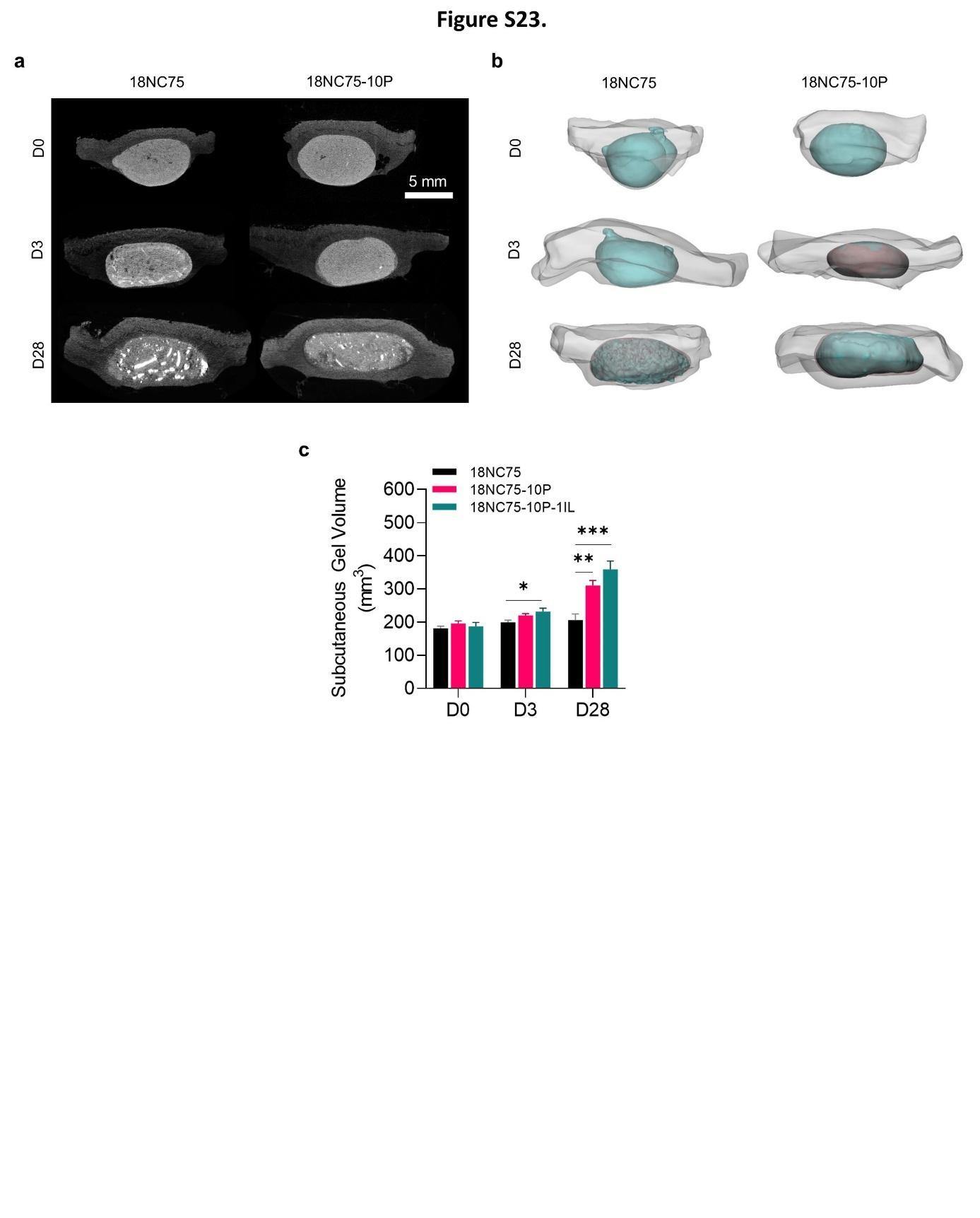


**Figure S25. Rat subcutaneous injection test. a,** Representative sagittal views of reconstructed micro-CT scans illustrating the high opacity of subcutaneously injected 18NC75 and 18NC75-10P, compared to the less radiodensity of the surrounding cutaneous tissue at D0, D3, and D28 post-injection. **b,** 3D rendering of subcutaneously injected 18NC75 and 18NC75-10P after segmentation from the surrounding cutaneous tissue at D0, D3, and D28 post-injection (see Figure S22a). **c,** Micro-CT analysis of subcutaneously injected volume of 18NC75, 18NC75-10P, and 18NC75-10P-1IL at D0, D3, and D28 post-injection based (D0; n=4, D3 and D28; n=6). Data are mean ± s.e.m.; statistical significance was determined by two-way ANOVA with Tukey’s post-hoc tests. *p < 0.05, **p < 0.01, ***p < 0.001.


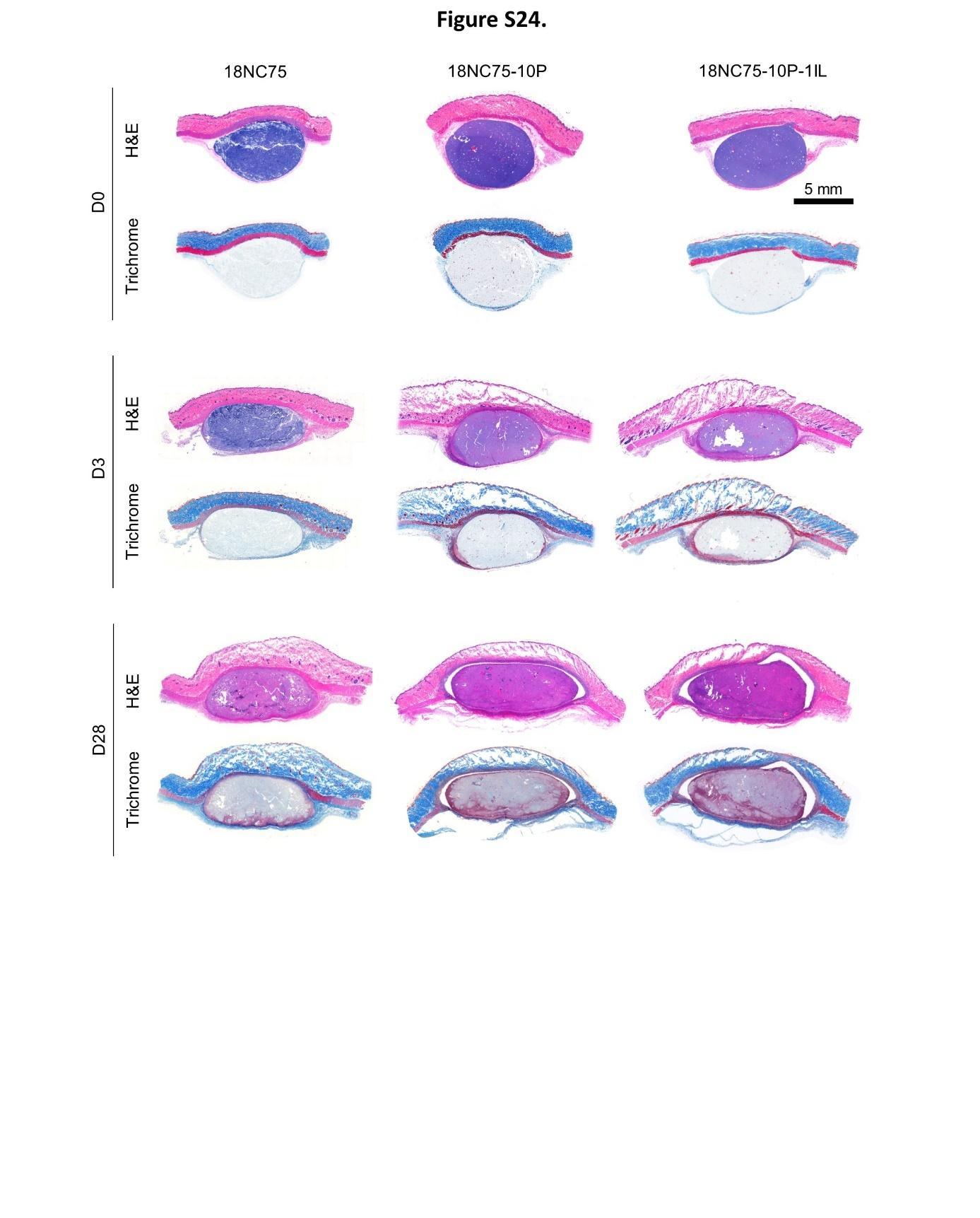


**Figure S26. Histology images of subcutaneous injection sites.** A panel of tiled images derived from histological sections stained with H&E or Masson’s trichrome, corresponding to the subcutaneous injection sites of 18NC75, 18NC75-10P, and 18NC75-10P-1IL at D0, D3, and D28 post-injection depicting time-dependent morphometric changes in each hydrogel.


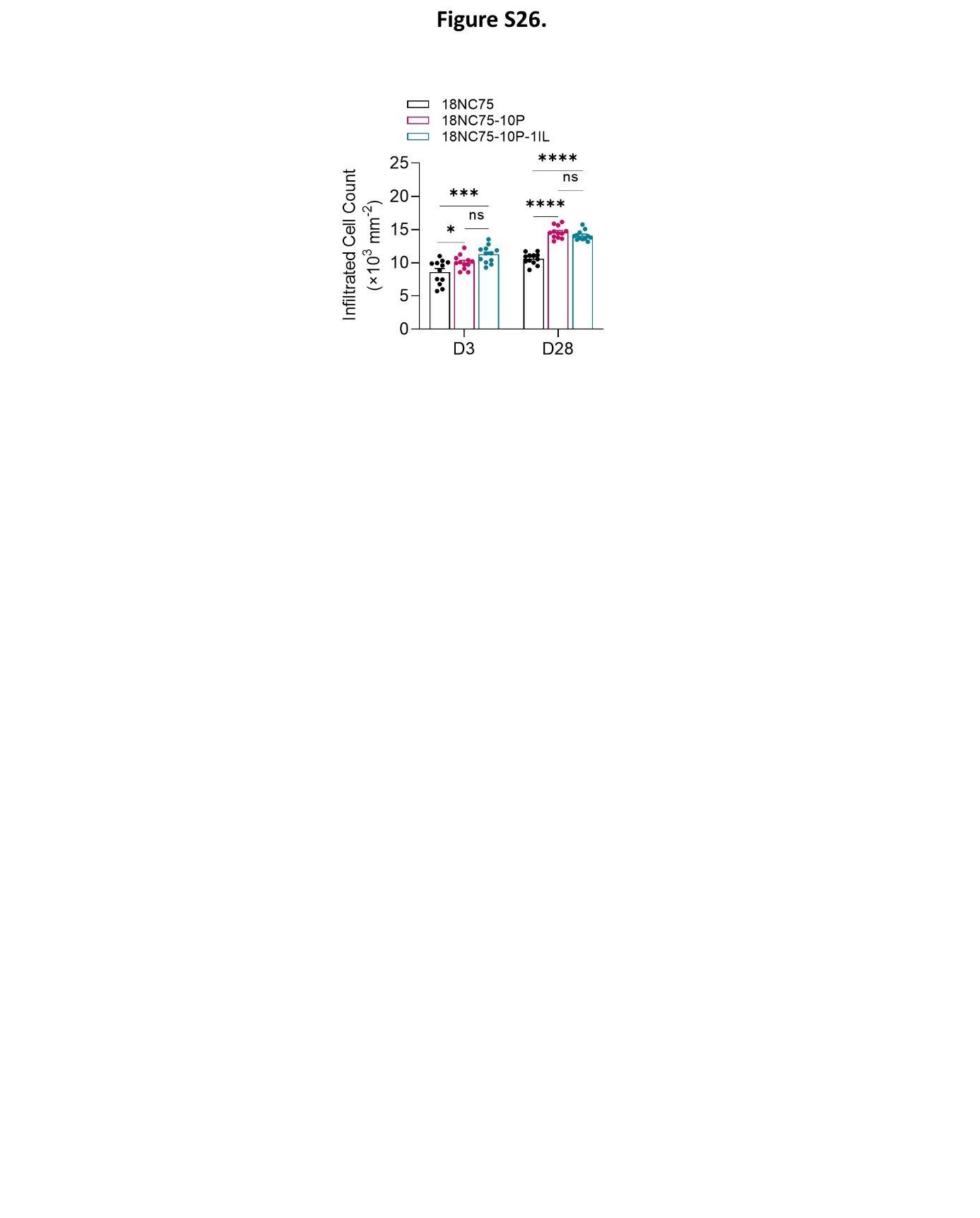


**Figure S27.** Graph depicting the average count of infiltrated cells within the dorsally injected site at D3 and D28 after the injection with 18NC75, 18NC75-10P, and 18NC75-10P-1IL. Data are expressed as mean ± s.e.m., and statistical significance was evaluated using a two-way ANOVA with Tukey’s multiple-comparison tests. ns, not significant, *p < 0.05, **p < 0.01, ***p < 0.001, ****p < 0.0001. The sample size for each analysis was n = 12.


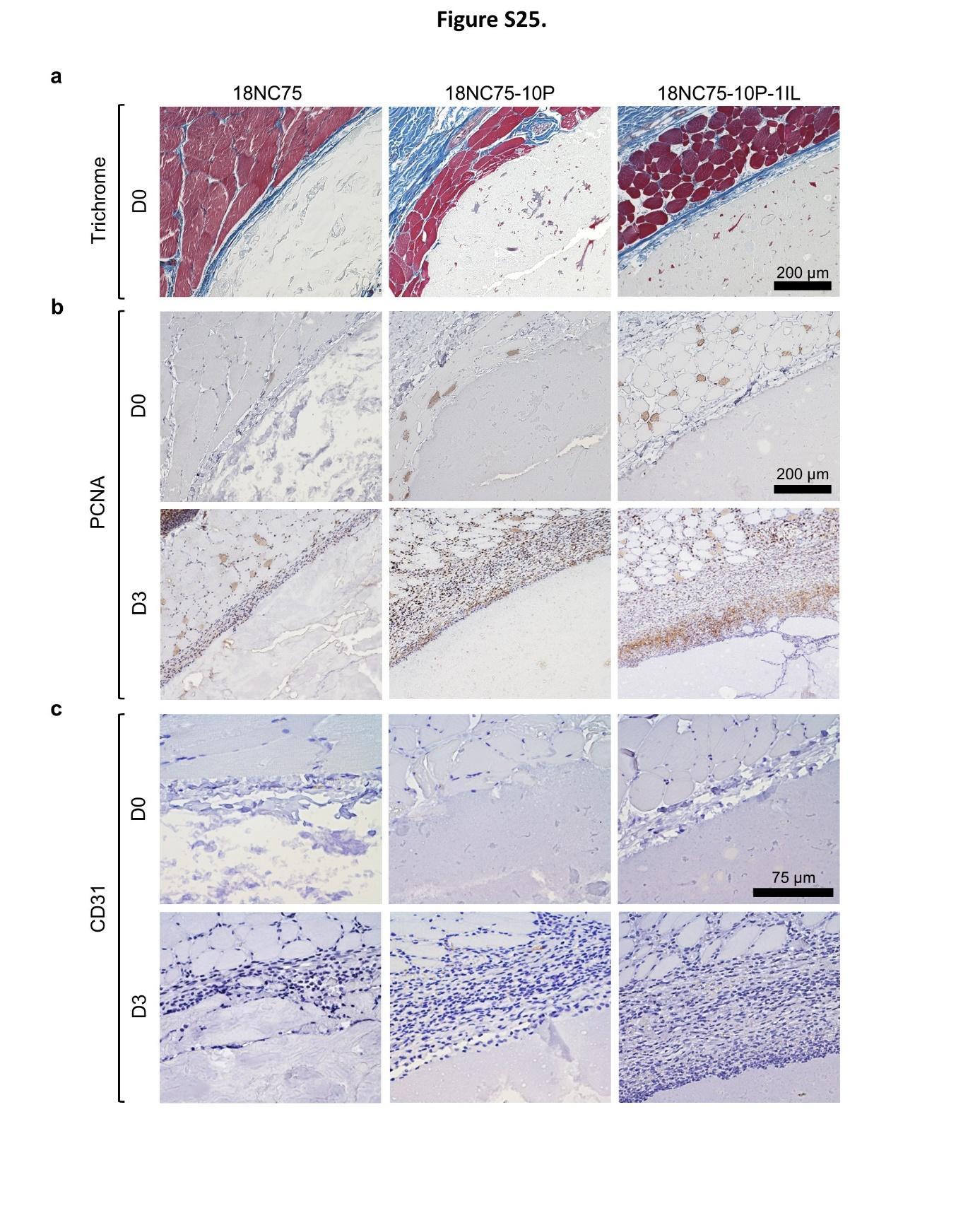


**Figure S28.** Representative images of Masson’s trichrome, PCNA^+^, and CD31^+^ immunostained histology sections corresponding to the subcutaneous injection sites of 18NC75, 18NC75-10P, and 18NC75-10P-1IL at D0 and D3 post-injection.


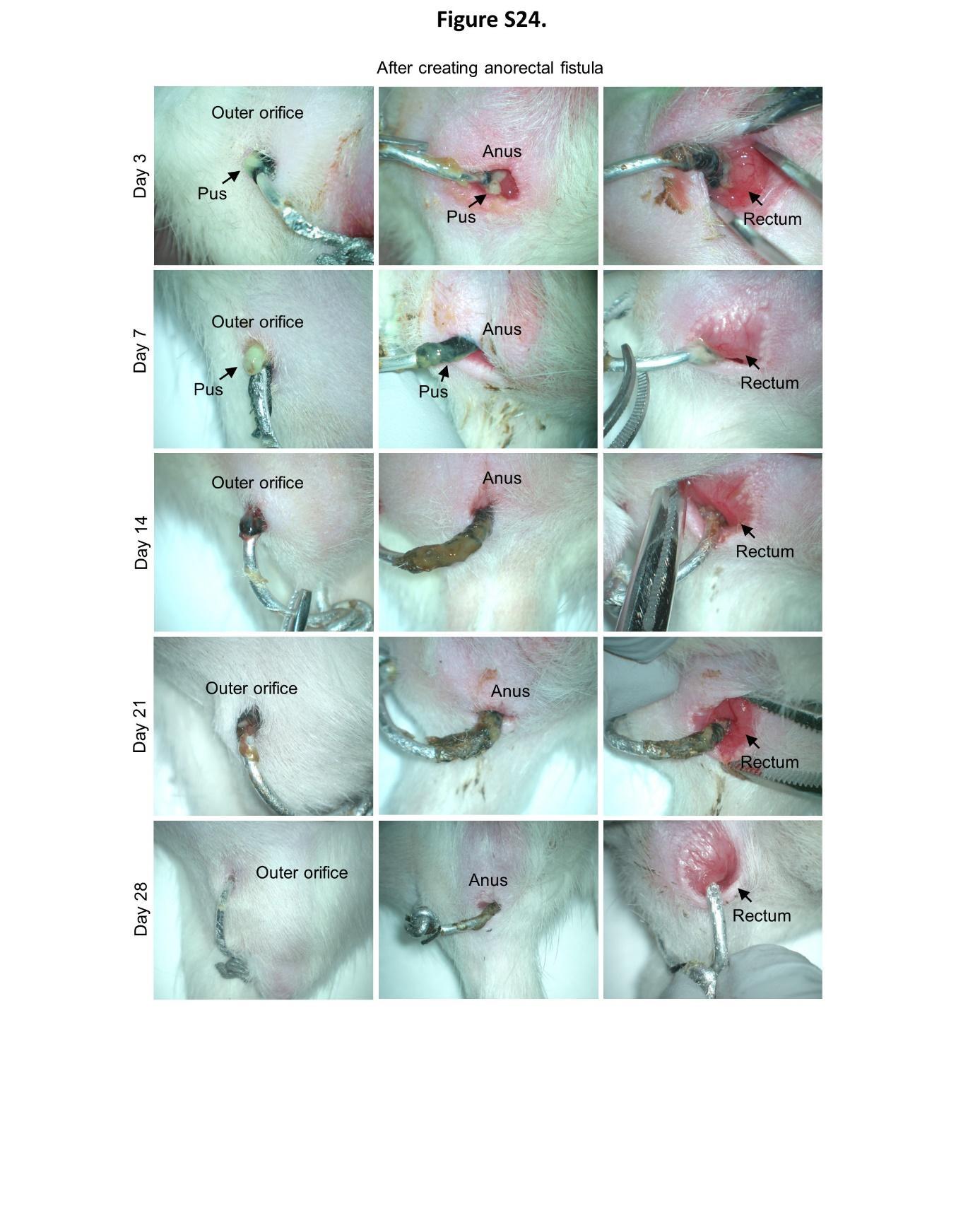


**Figure S29. Rat model of anorectal fistula.** Image panel depicting the outer orifice, anus, and rectum at various time points (D3, D7, D14, D21, and D28) following the induction of an infected anorectal fistula in rats.


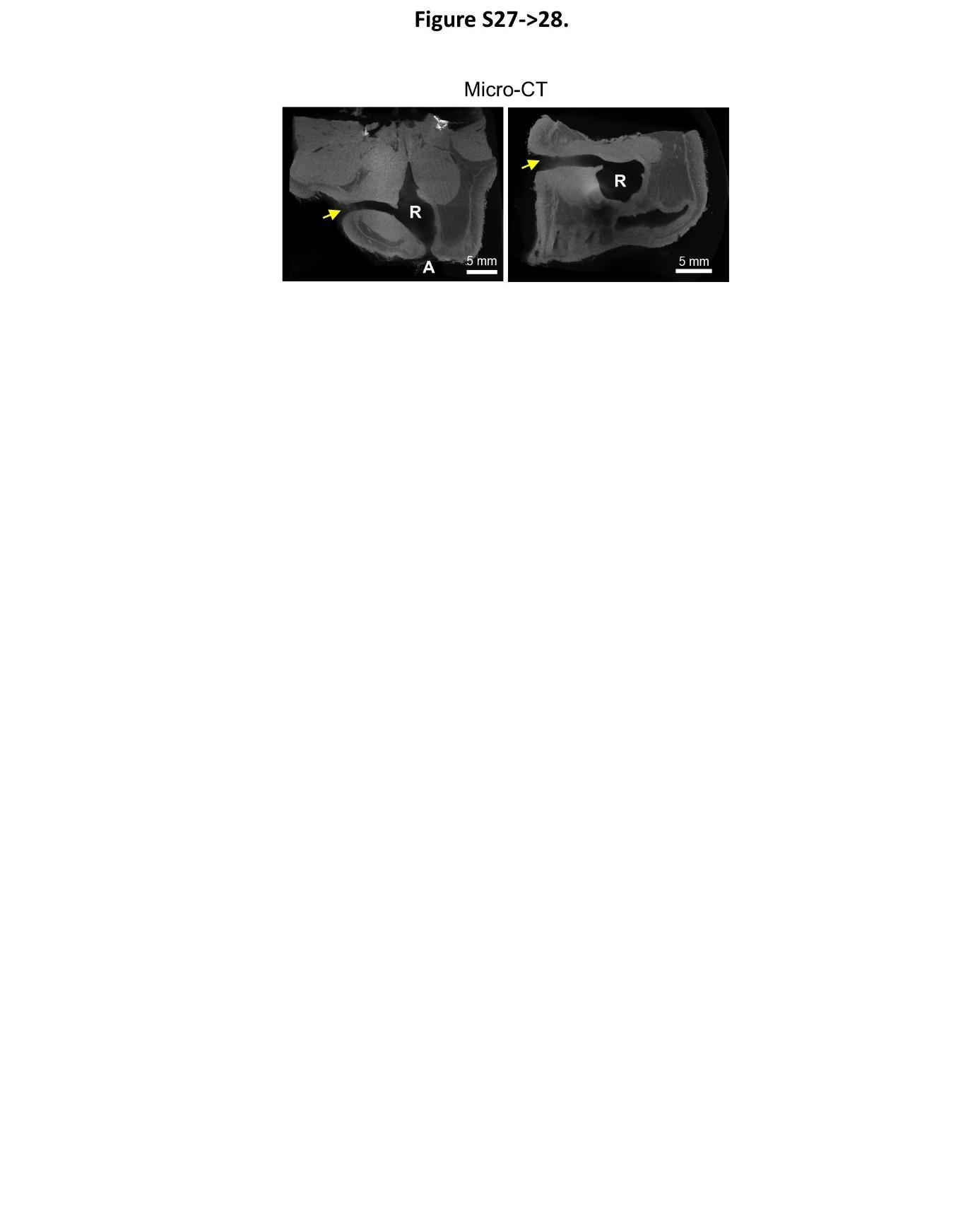
**Figure S30. Confirmation of anorectal fistula formation through micro-CT imaging.** Micro-CT scanning reveals the presence of an anorectal fistula in a rat, establishing a direct connection between the anorectal space and the skin. Key anatomical structures are labeled as follows: R (rectum), A (anus), and a yellow arrow indicates the outer orifice.


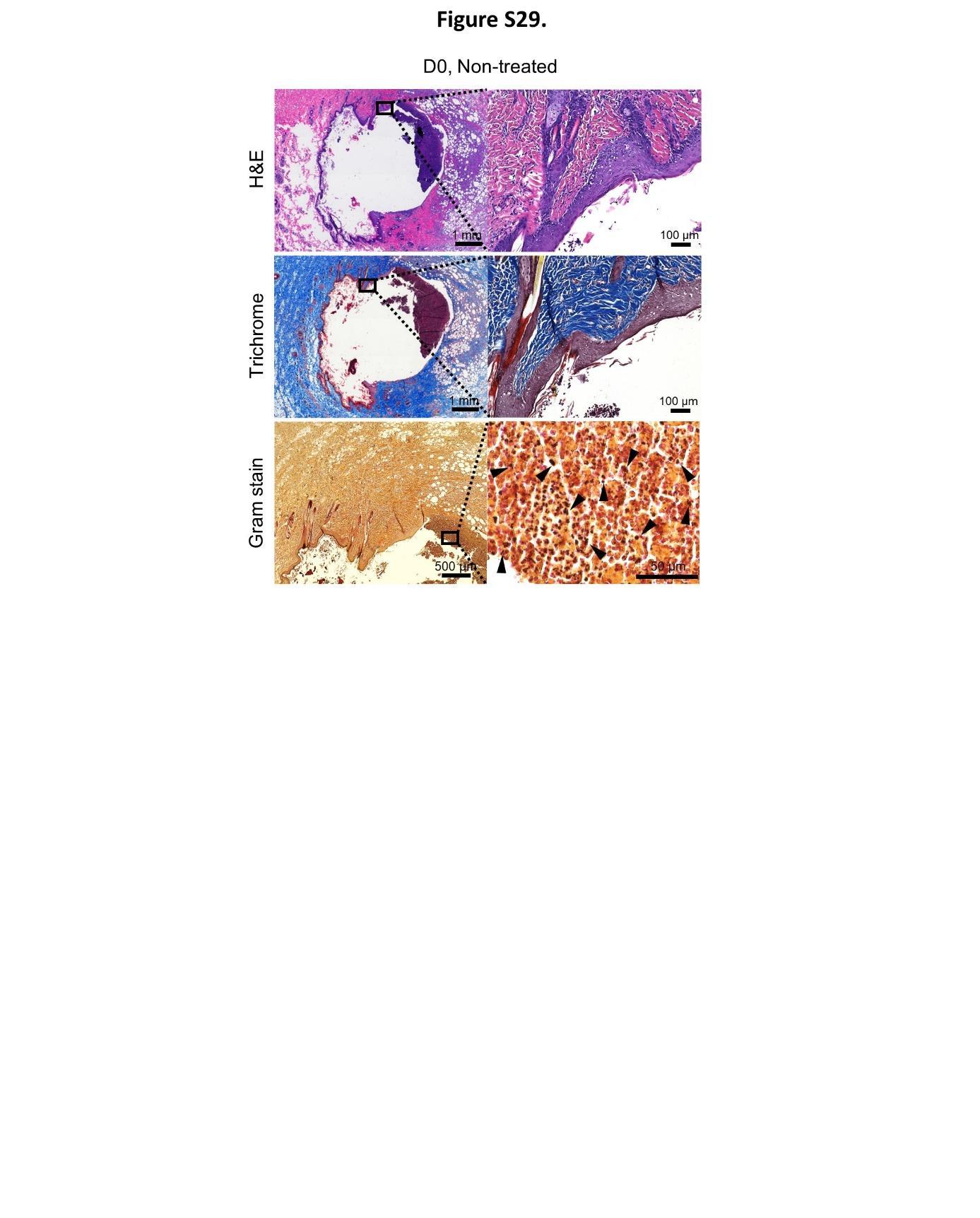
**Figure S31. Histological analysis of an untreated anorectal fistula post-wire removal.** Representative sections stained with Hematoxylin and Eosin (H&E), Masson's trichrome, and Gram stain respectively, provide insights into the untreated anorectal fistula after wire removal. The H&E-stained section reveals the fistula lumen surrounded by periluminal tissue with marked inflammatory cell infiltration, offering a detailed view of the fistula wall morphology and the host response. Masson's trichrome staining highlights the structural components, aiding in the assessment of collagen deposition and tissue architecture within the fistula tract. The Gram-stained section demonstrates substantial detection of Gram-positive bacteria, confirming the successful induction of infection in the fistula tract.


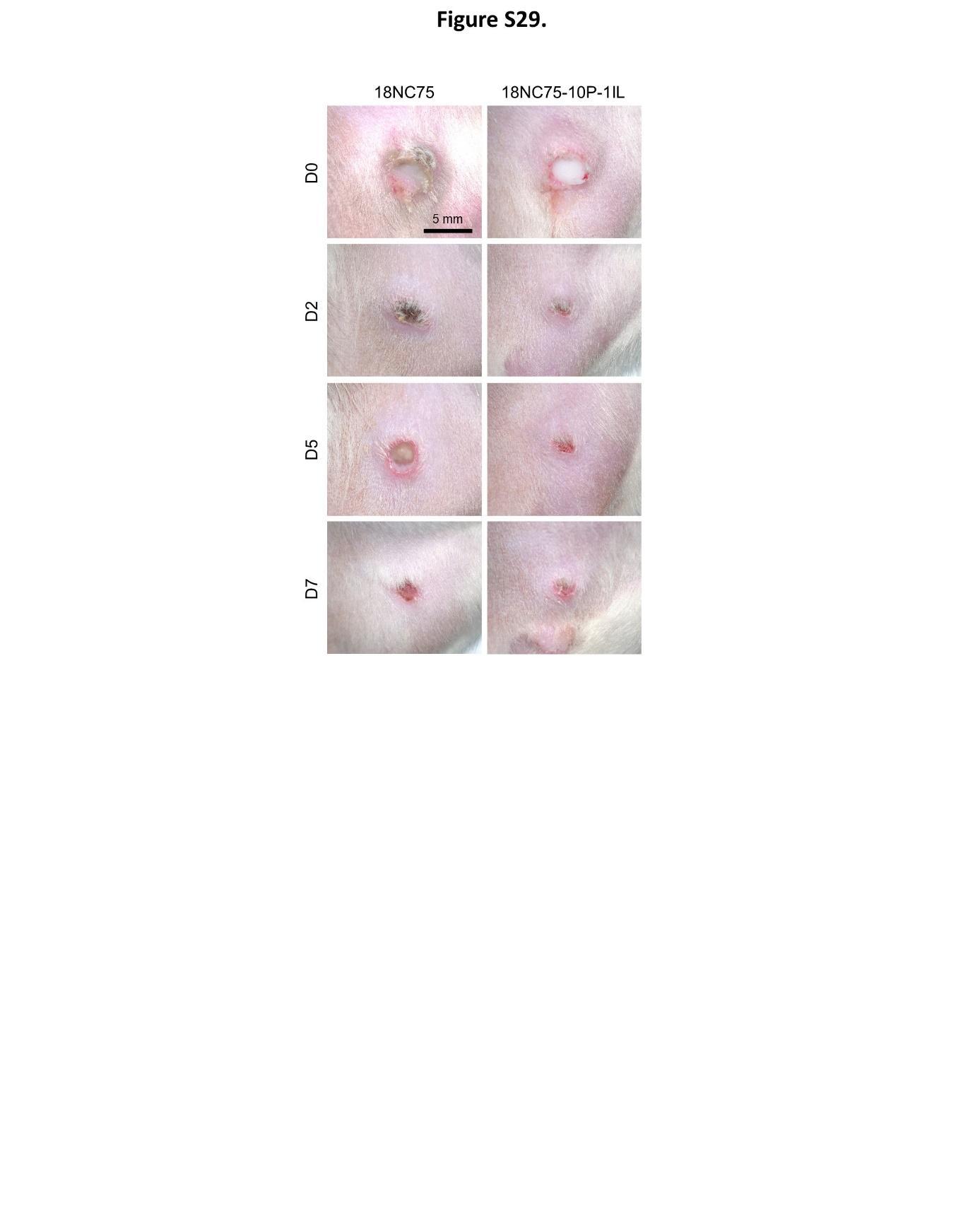


**Figure S32. Monitoring of the rat anorectal fistula models after material injection.**
Photographs of the outer orifice of a rat anorectal fistula at D0, D2, D5, and D7 after injection of 18NC75 or 18NC75-10P-1IL.


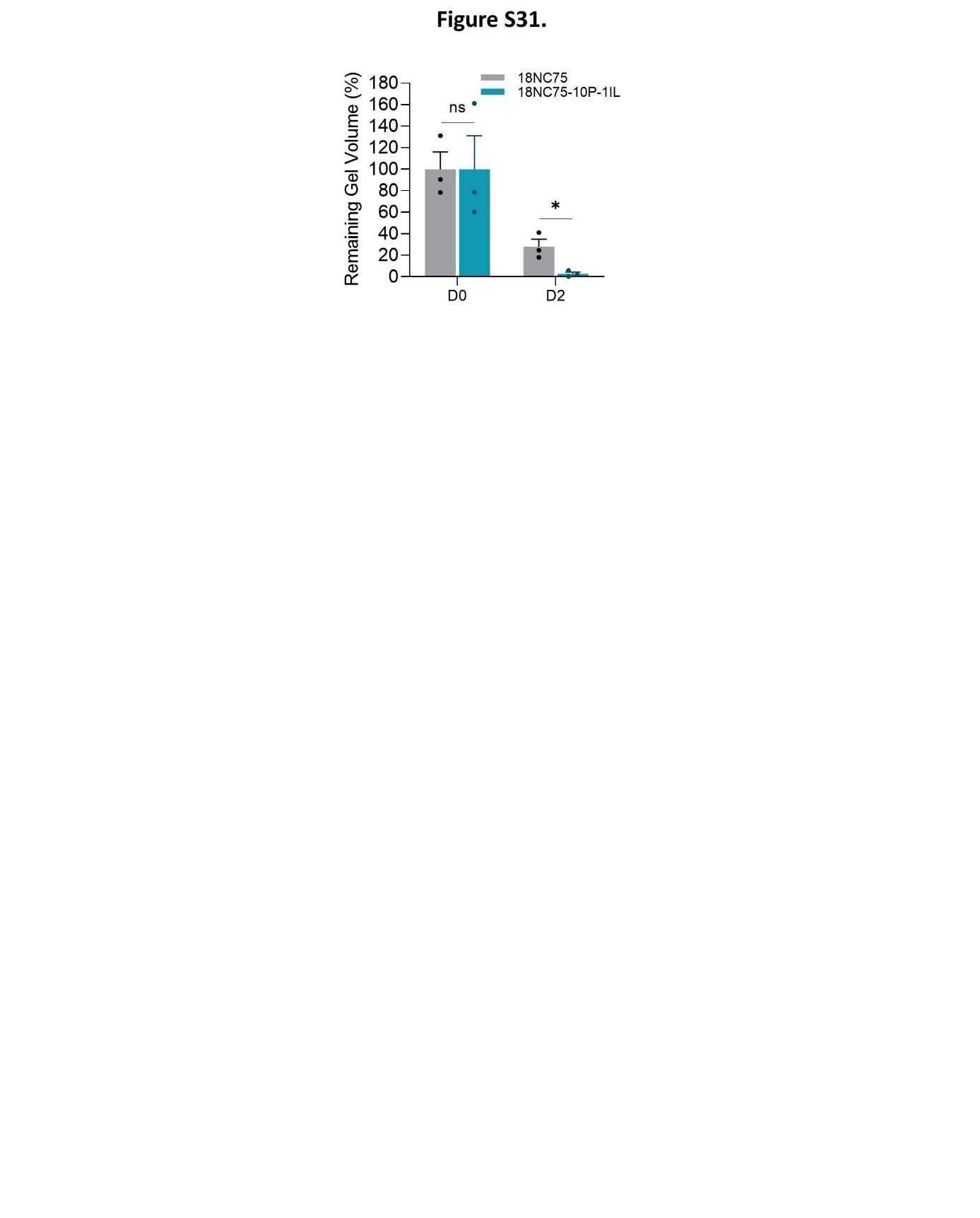


**Figure S33.** Measurement of remaining hydrogel volume (%) of 18NC75 or 18NC75-10P-1IL based on micro-CT scanning at D0 and D2 (n=3). Data are mean ± s.e.m.; statistical significance was determined by unpaired Student’s t-test. ns, not significant, *p < 0.05, **p < 0.01, ***p < 0.001, ****p < 0.0001.


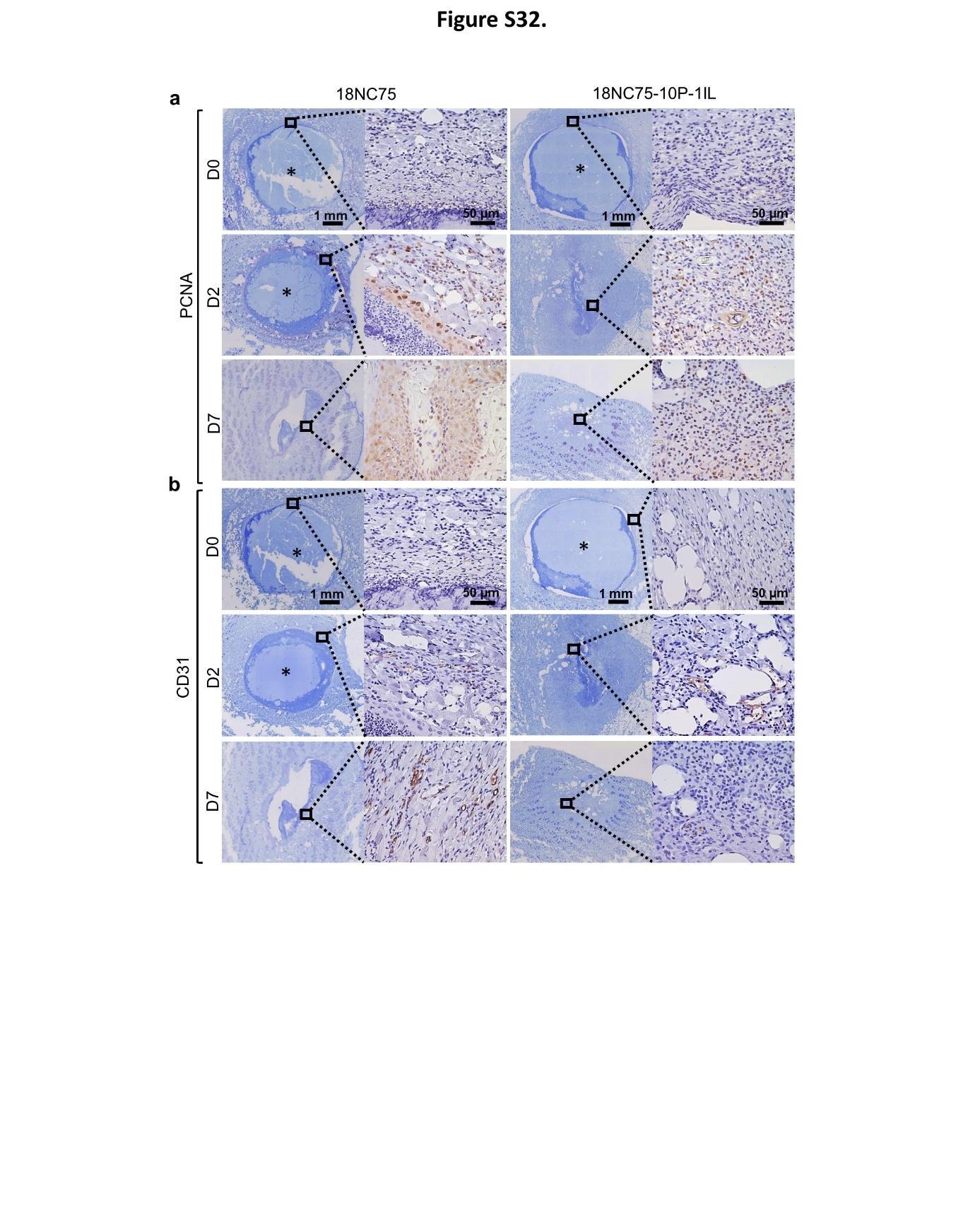


**Figure S34.** Representative immunohistochemistry images for PCNA^+^ (a) and CD31^+^ (b) cells in the histology sections of rat anorectal fistulas injected with 18NC75 or 18NC75-10P-1IL at D0, D2, and D7. Asterisks indicate the injected hydrogels.


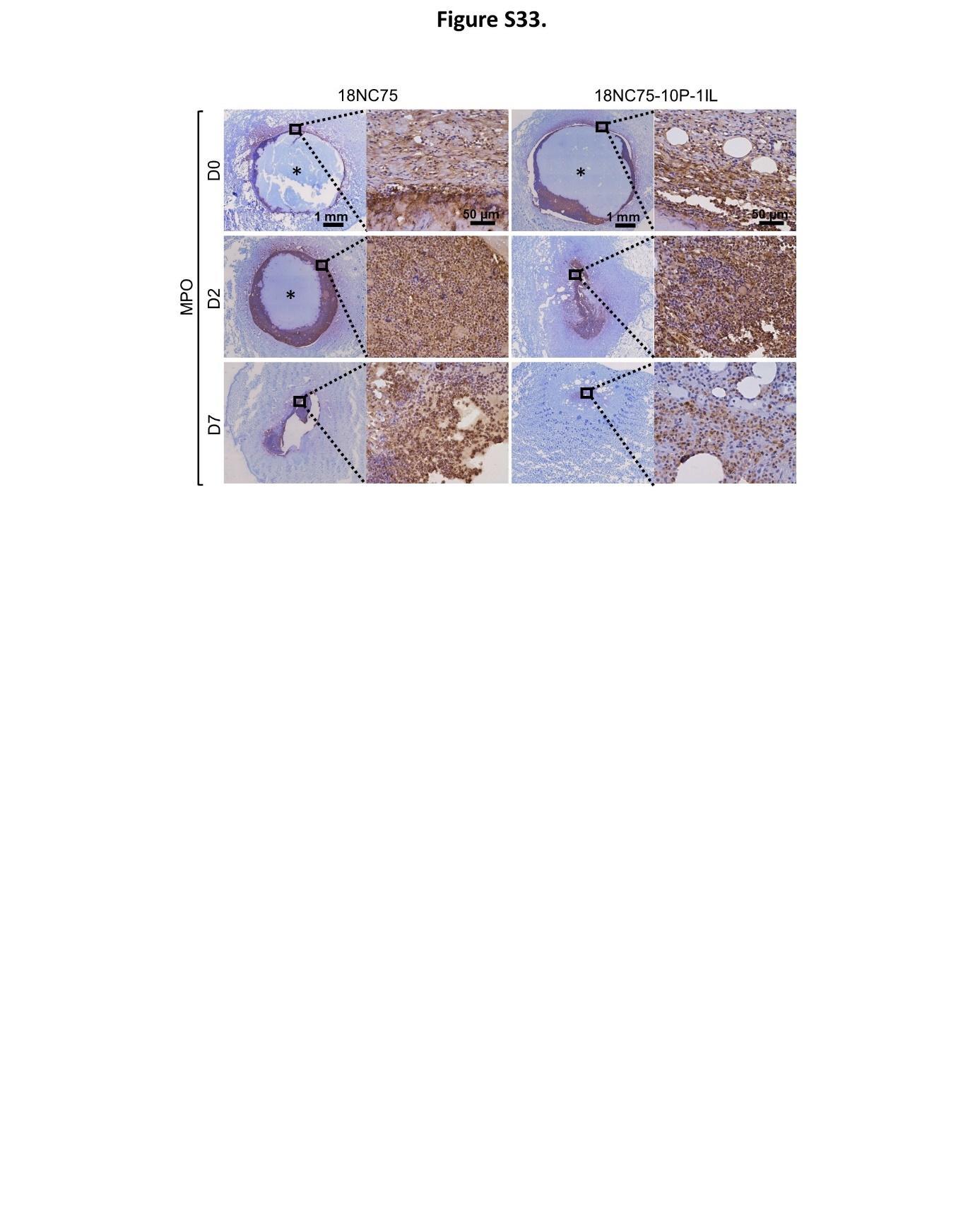


**Figure S35.** Representative immunohistochemistry images for MPO^+^ cells in the histology sections of rat anorectal fistulas injected with 18NC75 or 18NC75-10P-1IL at D0, D2, and D7. Asterisks indicate the injected hydrogels.


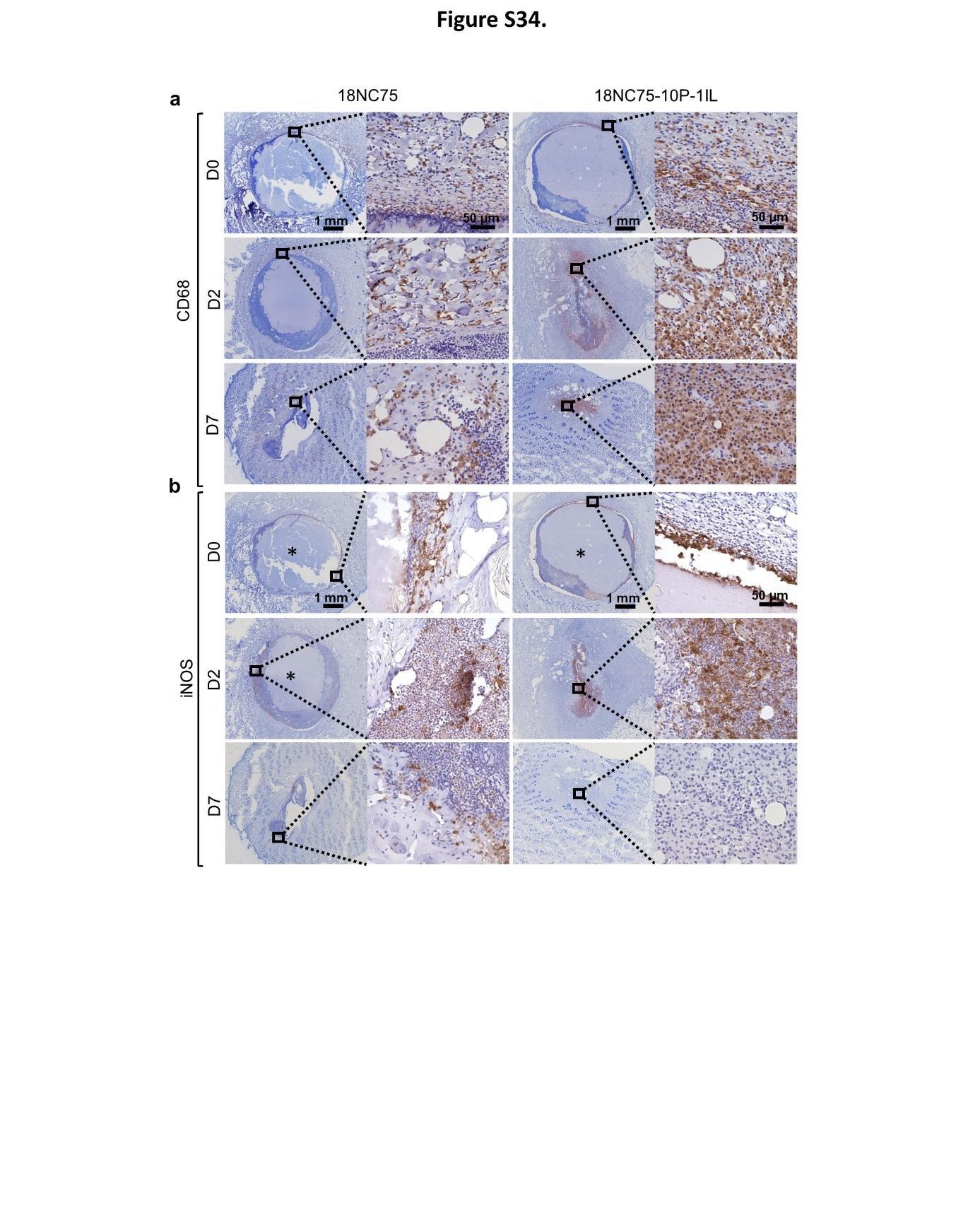
 **Figure S36.** Representative immunohistochemistry images for CD68^+^ (a) and iNOS^+^ (b) cells in the histology sections of rat anorectal fistulas injected with 18NC75 or 18NC75-10P-1IL at D0, D2, and D7. Asterisks indicate the injected hydrogels.


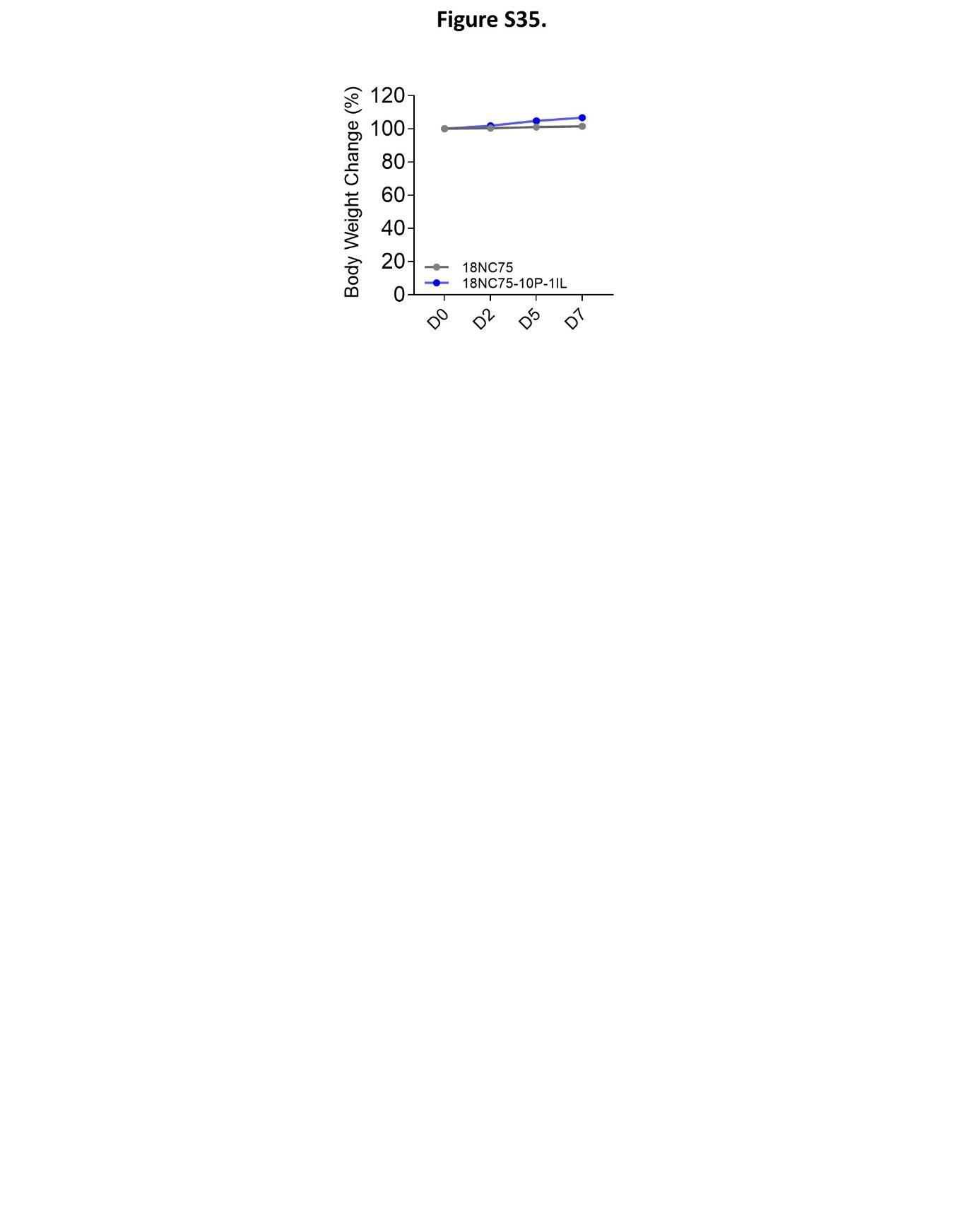


**Figure S37.** Body weight changes in rats with anorectal fistulas injected with 18NC75 or 18NC75-10P-1IL (n=3). Data are mean ± s.e.m.


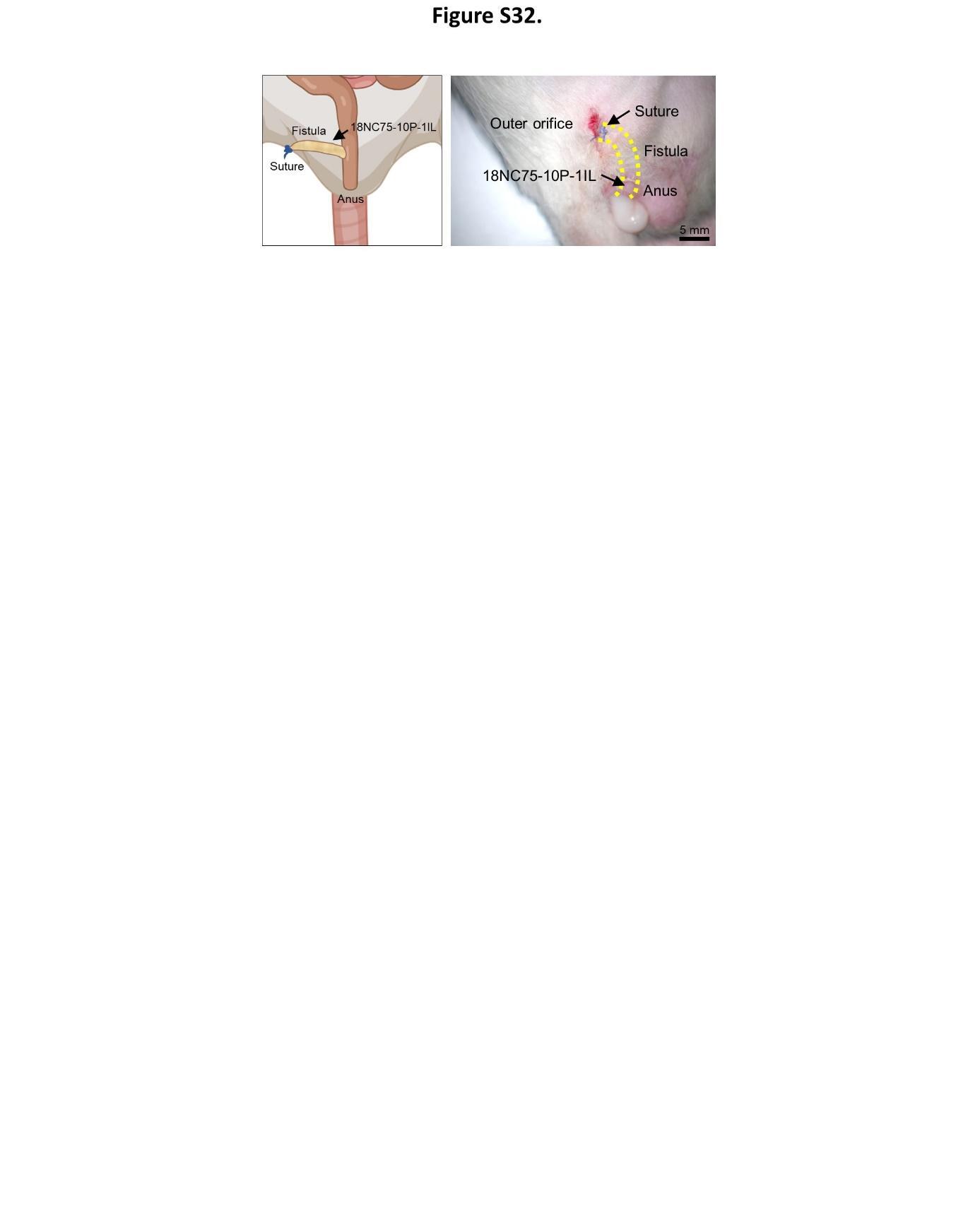


**Figure S38.** A schematic and a photograph illustrating the method of creating the modified anorectal fistula model with a suture in the outer orifice.


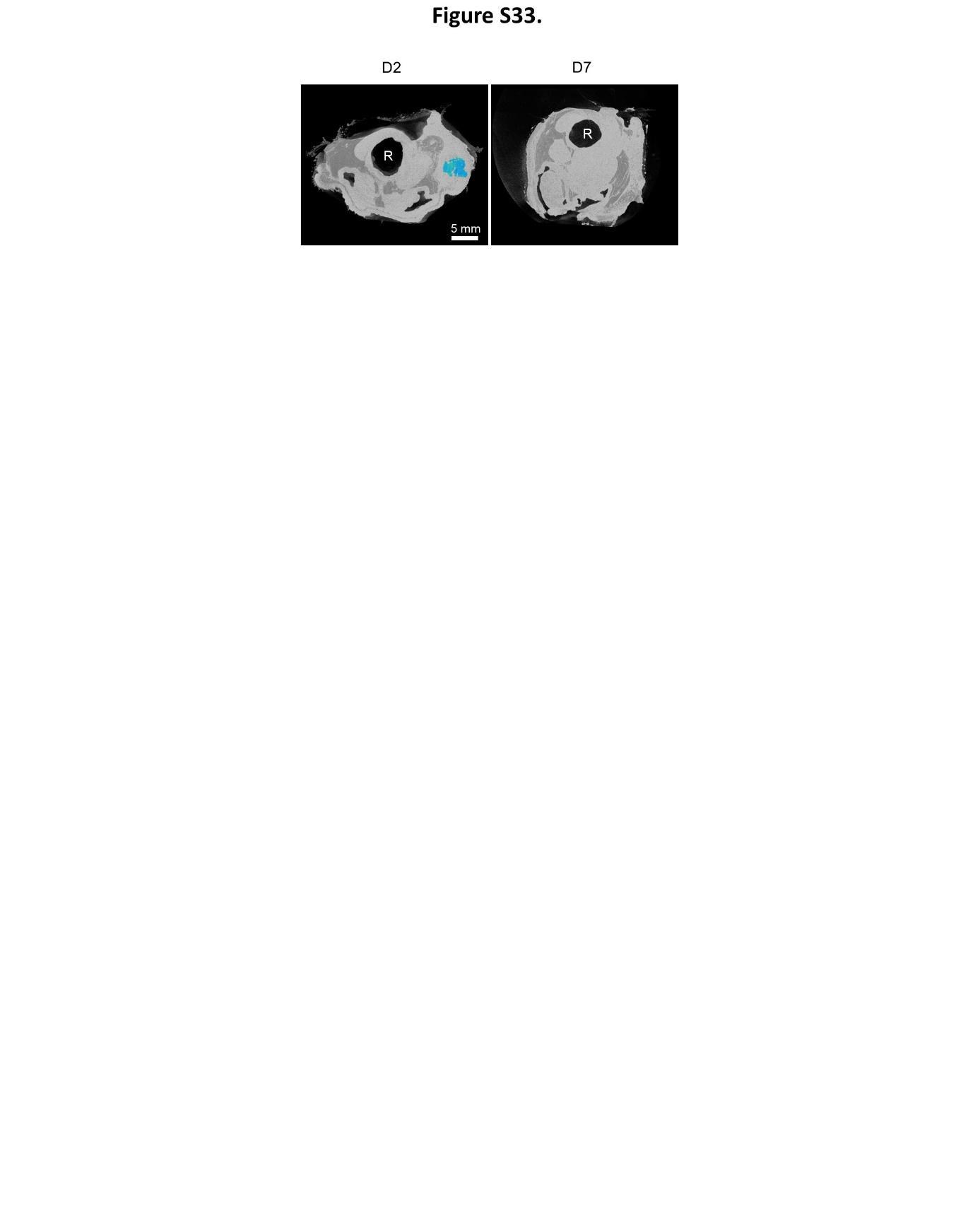


**Figure S39.** Micro-CT images displaying segmented hydrogel within the fistula tracts in blue at D2 and D7 after the injection of 18NC75-10P-1IL, followed by closure of the outer orifice with a suture.


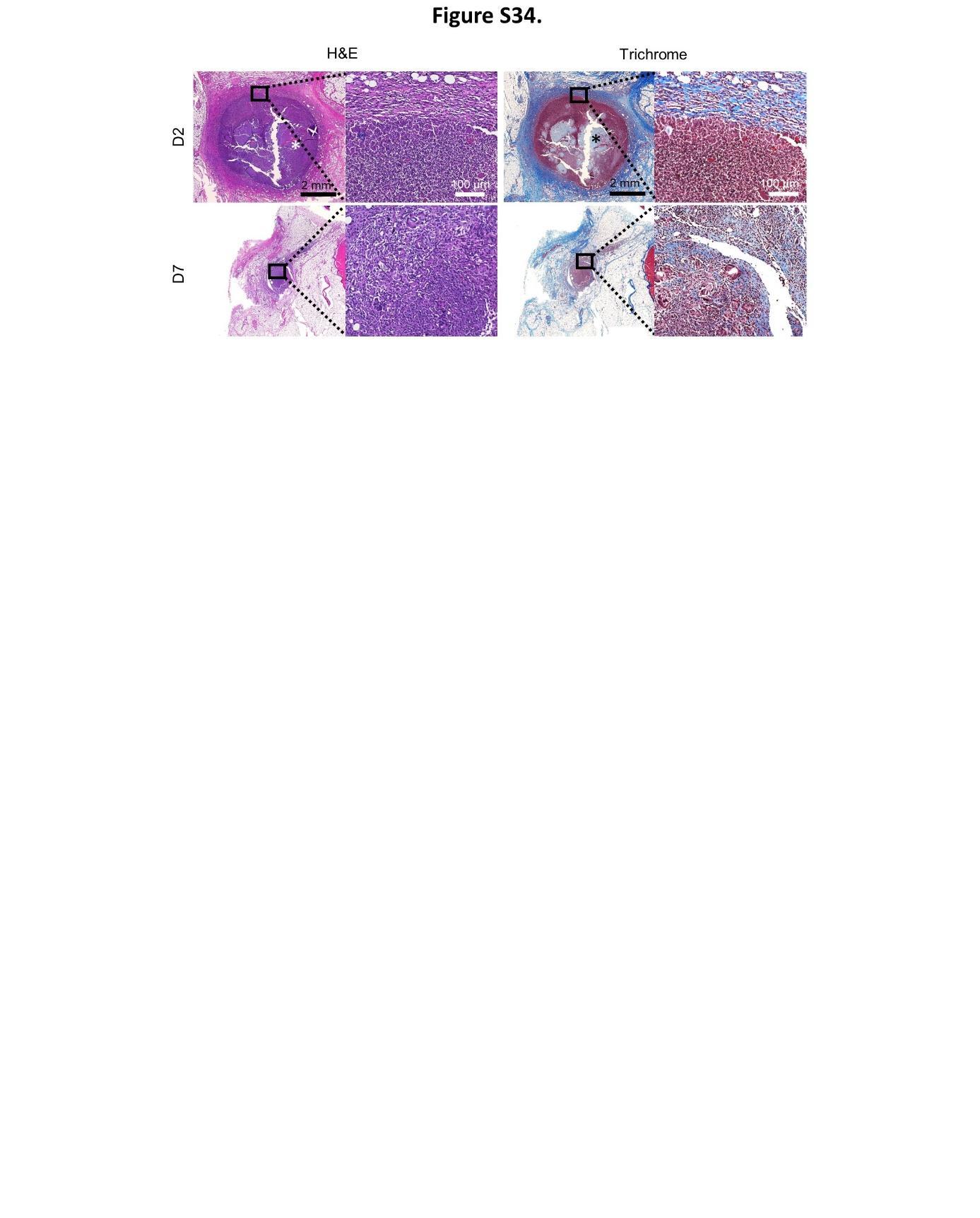


**Figure S40.** Representative H&E- and Masson’s trichrome-stained histology sections of rat anorectal fistulas at D2 and D7 after injection with 18NC75-10P-1IL and closure of the outer orifice with a suture. The asterisks indicate the injected hydrogels.


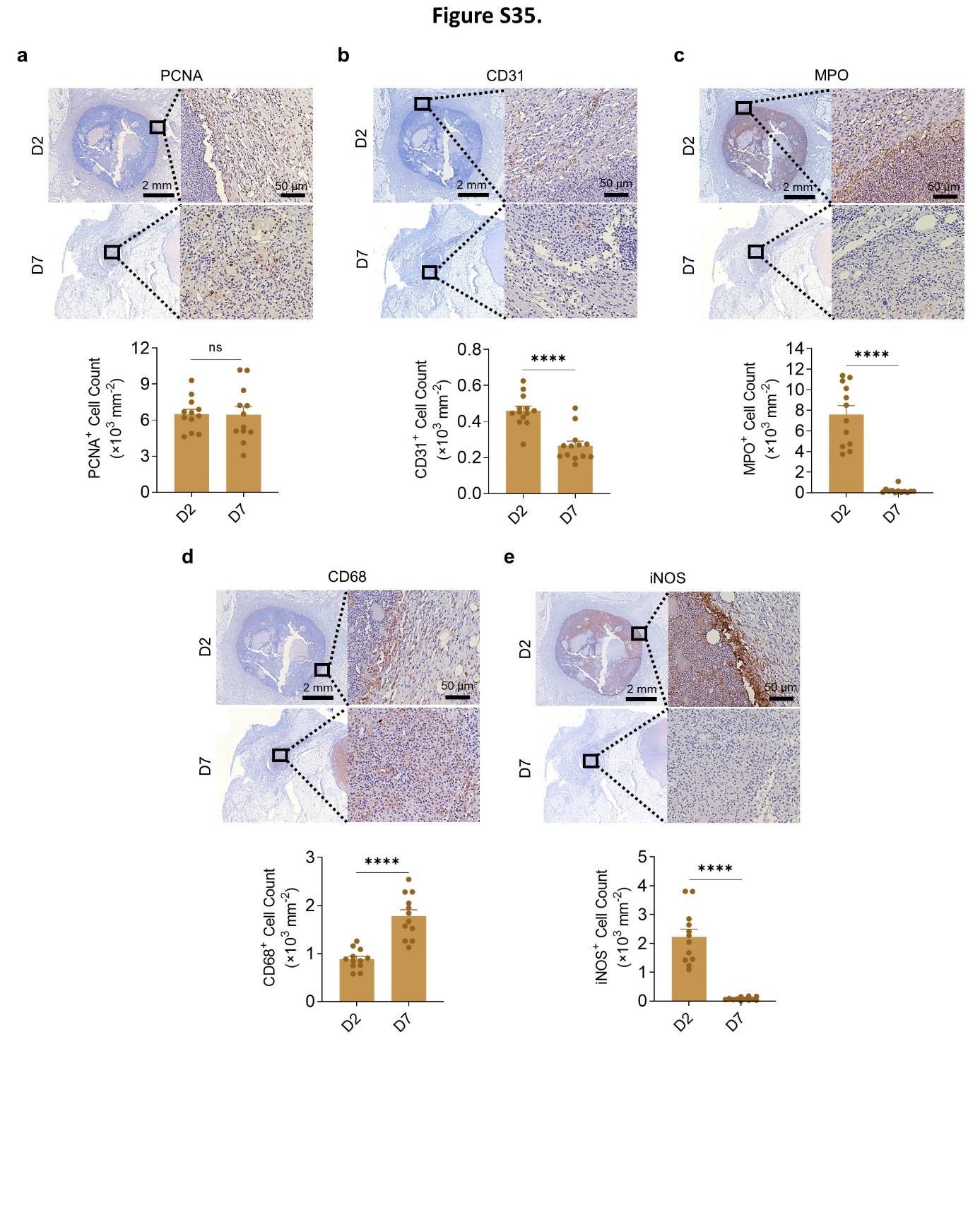


**Figure S41.** Representative immunohistochemistry images and average counts of PCNA^+^ (a), CD31^+^ (b), MPO^+^ (c), CD68^+^ (d), and iNOS^+^ (e) cells in 12 random fields from three individual histology sections of rat anorectal fistulas at D2 and D7 after injection with 18NC75-10P-1IL and closure of the outer orifice with a suture. (n=12) Data are mean ± s.e.m.; statistical significance was determined by unpaired Student’s t-test. ns, not significant, *p < 0.05, **p < 0.01, ***p < 0.001, ****p < 0.0001.


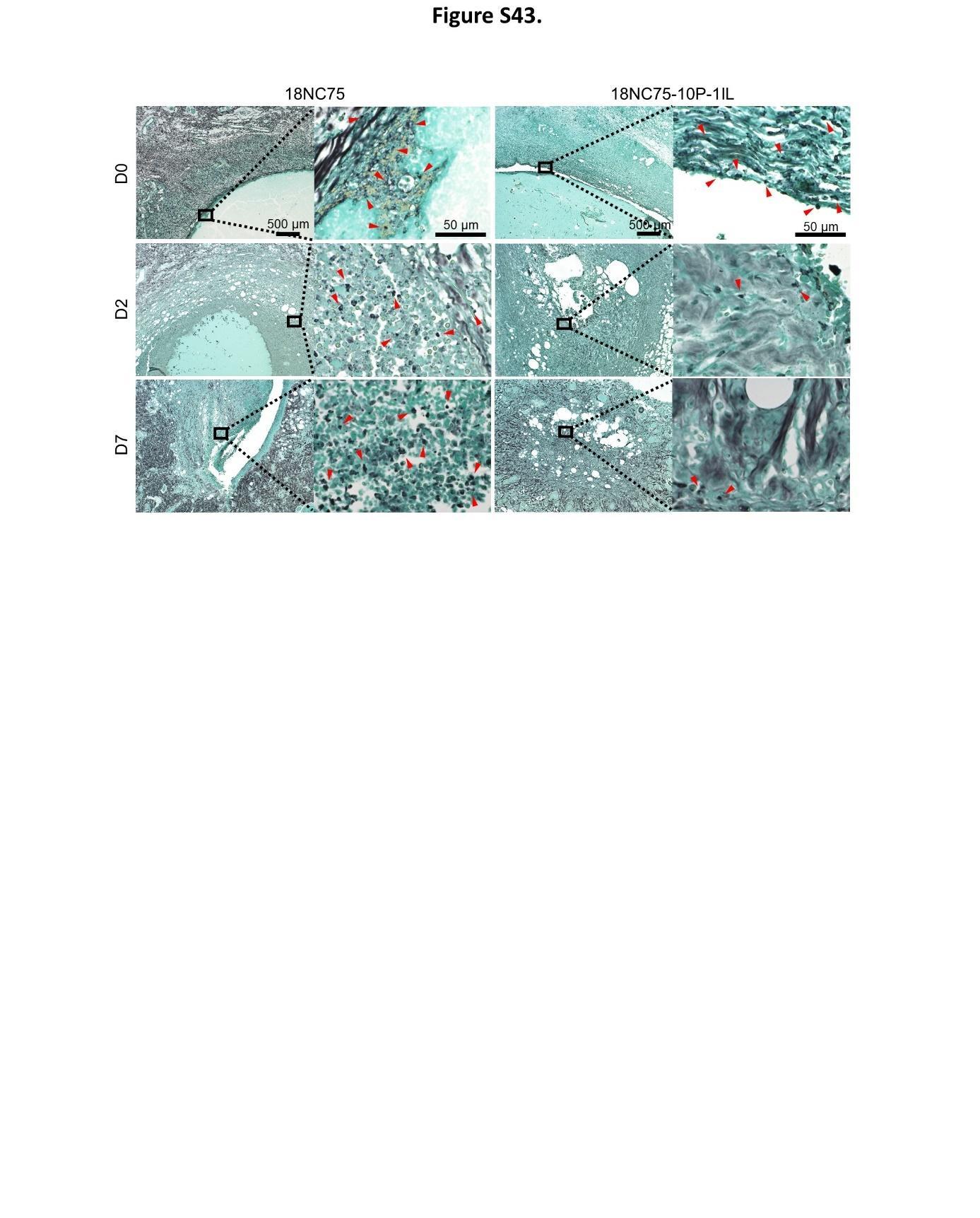


**Figure S42.** Representative Grocott’s methenamine silver (GMS) stained images of histology sections of rat anorectal fistulas injected with 18NC75 or 18NC75-10P-1IL at D0, D2, and D7 post injection. Arrowheads indicate fungi which were stained black.

**Table S1.** Serum chemistry levels in rats subcutaneously injected with 18NC75-10P-1IL at D3 and D28, compared to non-treated rats (Control). Data are mean ± s.e.m.; statistical significance was determined by two-way ANOVA with Tukey’s multiple-comparison test. ns, not significant, *p < 0.05, **p < 0.01.

| **Parameter [Unit]** | **Control** | **D3** | **D28** |
| --- | --- | --- | --- |
| Total Protein [g dL^-1^] | 6.07 ± 0.28 | 5.53 ± 0.19 ^ns^ | 7.40 ± 0.58 ^ns^ |
| Alkaline Phosphatase [U L^-1^] | 311.33 ± 49.59 | 338.67 ± 35.00 ^ns^ | 388.33 ± 32.77 ^ns^ |
| Glucose [mg dL^-1^] | 237.67 ± 54.78 | 338.00 ± 62.94* | 289.00 ± 24.33 ^ns^ |
| Alanine Aminotransferase [U L^-1^] | 48.67 ± 5.24 | 55.67 ± 10.20 ^ns^ | 68.00 ± 13.45 ^ns^ |
| Creatinine [mg dL^-1^] | 0.27 ± 0.07 | 0.23 ± 0.03 ^ns^ | 0.30 ± 0.00 ^ns^ |
| Blood Urea Nitrogen [mg dL^-1^] | 24.87 ± 3.35 | 21.10 ± 1.55 ^ns^ | 25.77 ± 1.23 ^ns^ |

**Table S2.** Serum cytokine/chemokine levels in rats subcutaneously injected with 18NC75-10P-1IL at D3 and D28, compared to non-treated rats (Control). Data are mean ± s.e.m.; statistical significance was determined by two-way ANOVA with Tukey’s multiple-comparison test. ns, not significant.

| **Parameter [Unit]** | **Control** | **D3** | **D28** |
| --- | --- | --- | --- |
| EGF [pg mL^-1^] | 8.74 ± 0.83 | 18.65 ± 7.65 ^ns^ | 8.46 ± 2.82 ^ns^ |
| Eotaxin [pg mL^-1^] | 28.16 ± 4.45 | 37.36 ± 14.75 ^ns^ | 41.49 ± 5.55 ^ns^ |
| Fractalkine [pg mL^-1^] | 81.18 ± 1.37 | 206.01 ± 53.70 ^ns^ | 35.30 ± 5.65 ^ns^ |
| G-CSF [pg mL^-1^] | 191.40 ± 78.15 | 485.84 ± 226.41 ^ns^ | 288.37 ± 102.10 ^ns^ |
| GM-CSF [pg mL^-1^] | 542.69 ± 109.10 | 793.31 ± 177.79 ^ns^ | 799.11 ± 306.58 ^ns^ |
| GRO/KC/CINC-1 [pg mL^-1^] | 150.01 ± 18.55 | 150.44 ± 49.46 ^ns^ | 198.12 ± 41.90 ^ns^ |
| IFNγ [pg mL^-1^] | 637.62 ± 293.48 | 1,235.37 ± 539.60 ^ns^ | 908.22 ± 166.58 ^ns^ |
| IL-1α [pg mL^-1^] | 151.22 ± 78.45 | 297.37 ± 136.87 ^ns^ | 245.77 ± 76.82 ^ns^ |
| IL-1β [pg mL^-1^] | 117.50 ± 40.25 | 358.62 ± 147.86 ^ns^ | 132.24 ± 40.19 ^ns^ |
| IL-2 [pg mL^-1^] | 356.37 ± 47.64 | 1,568.12 ± 658.79 ^ns^ | 143.04 ± 18.82 ^ns^ |
| IL-4 [pg mL^-1^] | 224.42 ± 102.81 | 447.98 ± 173.23 ^ns^ | 367.68 ± 71.19 ^ns^ |
| IL-5 [pg mL^-1^] | 321.51± 58.20 | 496.16 ± 146.31 ^ns^ | 415.72 ± 45.85 ^ns^ |
| IL-10 [pg mL^-1^] | 112.83 ± 33.81 | 339.63 ± 158.57 ^ns^ | 147.76 ± 18.99 ^ns^ |
| IL-12p70 [pg mL^-1^] | 1,354.07 ± 405.15 | 2,449.04 ± 835.50 ^ns^ | 1,903.01 ± 456.58 ^ns^ |
| IL-13 [pg mL^-1^] | 164.89 ± 32.33 | 255.86 ± 73.45 ^ns^ | 198.90 ± 46.12 ^ns^ |
| IL-17A [pg mL^-1^] | 255.58 ± 73.45 | 525.84 ± 156.69 ^ns^ | 399.49 ± 141.77 ^ns^ |
| IL-18 [pg mL^-1^] | 457.45 ± 76.22 | 1,891.56 ± 664.23 ^ns^ | 532.28 ± 228.11 ^ns^ |
| IP-10 [pg mL^-1^] | 200.08 ± 4.79 | 282.57 ± 63.77 ^ns^ | 144.23 ± 2.79 ^ns^ |
| LIX [pg mL^-1^] | 2,330.66 ± 127.53 | 3,735.39 ± 217.26 ^ns^ | 2,755.29 ± 905.24 ^ns^ |
| MCP-1 [pg mL^-1^] | 2,210.74 ± 166.19 | 3,966.96 ± 769.67 ^ns^ | 2,075.59 ± 156.34 ^ns^ |
| MIP-1α [pg mL^-1^] | 28.62± 2.77 | 71.33 ± 20.56 ^ns^ | 16.51 ± 3.30 ^ns^ |
| MIP-2 [pg mL^-1^] | 98.20 ± 22.43 | 182.81 ± 11.93 ^ns^ | 125.97 ± 38.27 ^ns^ |
| RANTES [pg mL^-1^] | 2,133.30 ± 414.14 | 2,981.34 ± 192.77 ^ns^ | 1,151.68 ± 454.27 ^ns^ |
| TNFα [pg mL^-1^] | 42.11 ± 8.09 | 89.36 ± 27.50 ^ns^ | 53.20 ± 13.63 ^ns^ |
| VEGF [pg mL^-1^] | 72.98 ± 12.43 | 306.25 ± 98.10 ^ns^ | <LOD |

**Table S3.** Complete blood count (CBC) in rats with anorectal fistulas treated with 18NC75 or 18NC75-10P-1IL compared to non-treated rats (Control) at D0 (n=3). Data are mean ± s.e.m.; statistical significance was determined by two-way ANOVA with Tukey’s multiple-comparison test. ns, not significant.

| **Parameter [Unit]** | **Control** | **18NC75** | **18NC75-10P-1IL** |
| --- | --- | --- | --- |
| White Blood Cells [10^3^ uL^-1^] | 8.48 ± 0.91 | 10.06 ± 2.09 ^ns^ | 10.86 ± 1.96 ^ns^ |
| Neutrophils [10^3^ uL^-1^] | 1.02 ± 0.10 | 3.49 ± 1.19 ^ns^ | 2.72 ± 1.09 ^ns^ |
| Lymphocytes [10^3^ uL^-1^] | 6.88 ± 1.00 | 5.53 ± 1.19 ^ns^ | 7.28 ± 0.85 ^ns^ |
| Monocytes [10^3^ uL^-1^] | 0.47 ± 0.04 | 0.88 ± 0.26 ^ns^ | 0.75 ± 0.17 ^ns^ |
| Eosinophils [10^3^ uL^-1^] | 0.11 ± 0.02 | 0.15 ± 0.04 ^ns^ | 0.12 ± 0.03 ^ns^ |
| Basophils [10^3^ uL^-1^] | 0.00 ± 0.00 | 0.00 ± 0.00 ^ns^ | 0.00 ± 0.00 ^ns^ |
| Red Blood Cells [10^6^ uL^-1^] | 7.88 ± 0.31 | 7.63 ± 0.18 ^ns^ | 7.98 ± 0.06 ^ns^ |
| HCT (%) | 43.17 ± 1.88 | 41.83 ± 1.11 ^ns^ | 43.93 ± 0.73 ^ns^ |
| Platelet [10^3^ uL^-1^] | 958.33 ± 26.69 | 961.33 ± 51.45 ^ns^ | 958 ± 50.54 ^ns^ |

**Table S4.** Complete blood count (CBC) in rats with anorectal fistulas treated with 18NC75 or 18NC75-10P-1IL compared to non-treated rats (Control) at D2 (n=3). Data are mean ± s.e.m.; statistical significance was determined by two-way ANOVA with Tukey’s multiple-comparison test. ns, not significant, ****p < 0.0001.

| **Parameter [Unit]** | **Control** | **18NC75** | **18NC75-10P-1IL** |
| --- | --- | --- | --- |
| White Blood Cells [10^3^ uL^-1^] | 8.48 ± 0.91 | 12.75 ± 1.27 ^ns^ | 8.61 ± 0.82 ^ns^ |
| Neutrophils [10^3^ uL^-1^] | 1.02 ± 0.10 | 4.77 ± 1.83 ^ns^ | 1.93 ± 0.61 ^ns^ |
| Lymphocytes [10^3^ uL^-1^] | 6.88 ± 1.00 | 6.54 ± 0.56 ^ns^ | 6.05 ± 0.23 ^ns^ |
| Monocytes [10^3^ uL^-1^] | 0.47 ± 0.04 | 1.26 ± 0.30 ^ns^ | 0.49 ± 0.01 ^ns^ |
| Eosinophils [10^3^ uL^-1^] | 0.11 ± 0.02 | 0.15 ± 0.00 ^ns^ | 0.13 ± 0.01 ^ns^ |
| Basophils [10^3^ uL^-1^] | 0.00 ± 0.00 | 0.03 ± 0.02 ^ns^ | 0.01 ± 0.003 ^ns^ |
| Red Blood Cells [10^6^ uL^-1^] | 7.88 ± 0.31 | 7.36 ± 0.34 ^ns^ | 7.65 ± 0.22 ^ns^ |
| HCT (%) | 43.17 ± 1.88 | 41.23 ± 1.99 ^ns^ | 41.43 ± 0.78 ^ns^ |
| Platelet [10^3^ uL^-1^] | 958.33 ± 26.69 | 787.67 ± 106.19**** | 956.67 ± 13.86 ^ns^ |

**Table S5.** Complete blood count (CBC) in rats with anorectal fistulas treated with 18NC75 or 18NC75-10P-1IL compared to non-treated rats (Control) at D7 (n=3). Data are mean ± s.e.m.; statistical significance was determined by two-way ANOVA with Tukey’s multiple-comparison test. ns, not significant, **p < 0.01, ***p < 0.001.

| **Parameter [Unit]** | **Control** | **18NC75** | **18NC75-10P-1IL** |
| --- | --- | --- | --- |
| White Blood Cells [10^3^ uL^-1^] | 8.48 ± 0.91 | 3.99 ± 0.04 ^ns^ | 6.83 ± 0.50 ^ns^ |
| Neutrophils [10^3^ uL^-1^] | 1.02 ± 0.10 | 0.57 ± 0.01 ^ns^ | 0.76 ± 0.11 ^ns^ |
| Lymphocytes [10^3^ uL^-1^] | 6.88 ± 1.00 | 3.09 ± 0.04 ^ns^ | 5.57 ± 0.38 ^ns^ |
| Monocytes [10^3^ uL^-1^] | 0.47 ± 0.04 | 0.30 ± 0.01 ^ns^ | 0.44 ± 0.11 ^ns^ |
| Eosinophils [10^3^ uL^-1^] | 0.11 ± 0.02 | 0.04 ± 0.01 ^ns^ | 0.07 ± 0.003 ^ns^ |
| Basophils [10^3^ uL^-1^] | 0.00 ± 0.00 | 0.00 ± 0.00 ^ns^ | 0.00 ± 0.00 ^ns^ |
| Red Blood Cells [10^6^ uL^-1^] | 7.88 ± 0.31 | 8.16 ± 0.04 ^ns^ | 7.56 ± 0.03 ^ns^ |
| HCT (%) | 43.17 ± 1.88 | 42.27 ± 0.18 ^ns^ | 41.3 ± 0.12 ^ns^ |
| Platelet [10^3^ uL^-1^] | 958.33 ± 26.69 | 927.67 ± 15.7** | 939.67 ± 11.5 ^ns^ |

**Table S6.** Serum chemistry levels in rats with anorectal fistulas treated with 18NC75 or 18NC75-10P-1IL compared to non-treated rats (Control) at D0 (n=3). Data are mean ± s.e.m.; statistical significance was determined by two-way ANOVA with Tukey’s multiple-comparison test. ns, not significant.

| **Parameter [Unit]** | **Control** | **18NC75** | **18NC75-10P-1IL** |
| --- | --- | --- | --- |
| Total Protein [g dL^-1^] | 6.07 ± 0.28 | 5.63 ± 0.07 ^ns^ | 6.40 ± 0.10 ^ns^ |
| Alkaline Phosphatase [U L^-1^] | 311.33 ± 49.59 | 259.00 ± 21.66 ^ns^ | 287.00 ± 19.31 ^ns^ |
| Glucose [mg dL^-1^] | 237.67 ± 54.78 | 203.67 ± 27.17 ^ns^ | 205.00 ± 9.54 ^ns^ |
| Alanine Aminotransferase [U L^-1^] | 48.67 ± 5.24 | 50.33 ± 5.61 ^ns^ | 41.67 ± 2.40 ^ns^ |
| Creatinine [mg dL^-1^] | 0.27 ± 0.07 | 0.23 ± 0.03 ^ns^ | 0.23 ± 0.03 ^ns^ |
| Blood Urea Nitrogen [mg dL^-1^] | 24.87 ± 3.35 | 17.87 ± 2.08 ^ns^ | 20.83 ± 0.85 ^ns^ |

**Table S7.** Serum chemistry levels in rats with anorectal fistulas treated with 18NC75 or 18NC75-10P-1IL compared to non-treated rats (Control) at D2 (n=3). Data are mean ± s.e.m.; statistical significance was determined by two-way ANOVA with Tukey’s multiple-comparison test. ns, not significant, ***p < 0.001, ****p < 0.0001.

| **Parameter [Unit]** | **Control** | **18NC75** | **18NC75-10P-1IL** |
| --- | --- | --- | --- |
| Total Protein [g dL^-1^] | 6.07 ± 0.28 | 6.63 ± 0.12 ^ns^ | 6.47 ± 0.18 ^ns^ |
| Alkaline Phosphatase [U L^-1^] | 311.33 ± 49.59 | 576.67 ± 161.12*** | 382.67 ± 43.24 ^ns^ |
| Glucose [mg dL^-1^] | 237.67 ± 54.78 | 201.67 ± 11.61 ^ns^ | 220.67 ± 15.56 ^ns^ |
| Alanine Aminotransferase [U L^-1^] | 48.67 ± 5.24 | 57.33 ± 5.61 ^ns^ | 46.67 ± 2.19 ^ns^ |
| Creatinine [mg dL^-1^] | 0.27 ± 0.07 | 0.27 ± 0.03 ^ns^ | 0.30 ± 0.00 ^ns^ |
| Blood Urea Nitrogen [mg dL^-1^] | 24.87 ± 3.35 | 22.70 ± 0.91 ^ns^ | 23.90 ± 1.71 ^ns^ |

**Table S8.** Serum chemistry levels in rats with anorectal fistulas treated with 18NC75 or 18NC75-10P-1IL compared to non-treated rats (Control) at D7 (n=3). Data are mean ± s.e.m.; statistical significance was determined by two-way ANOVA with Tukey’s multiple-comparison test. ns, not significant.

| **Parameter [Unit]** | **Control** | **18NC75** | **18NC75-10P-1IL** |
| --- | --- | --- | --- |
| Total Protein [g dL^-1^] | 6.07 ± 0.28 | 6.27 ± 0.19 ^ns^ | 5.97 ± 0.19 ^ns^ |
| Alkaline Phosphatase [U L^-1^] | 311.33 ± 49.59 | 340.67 ± 16.23 ^ns^ | 350.67 ± 37.83 ^ns^ |
| Glucose [mg dL^-1^] | 237.67 ± 54.78 | 181.33 ± 6.94 ^ns^ | 181.67 ± 5.70 ^ns^ |
| Alanine Aminotransferase [U L^-1^] | 48.67 ± 5.24 | 56.00 ± 2.52 ^ns^ | 47.33 ± 3.48 ^ns^ |
| Creatinine [mg dL^-1^] | 0.27 ± 0.07 | 0.20 ± 0.00 ^ns^ | 0.20 ± 0.00 ^ns^ |
| Blood Urea Nitrogen [mg dL^-1^] | 24.87 ± 3.35 | 18.90 ± 1.55 ^ns^ | 18.87 ± 0.15 ^ns^ |

**Table S9.** Serum cytokine/chemokine levels in rats with anorectal fistulas treated with 18NC75 or 18NC75-10P-1IL compared to non-treated rats (Control) at D0 (n=3). Data are mean ± s.e.m.; statistical significance was determined by two-way ANOVA with Tukey’s multiple-comparison test. ns, not significant.

| **Parameter [Unit]** | **Control** | **18NC75** | **18NC75-10P-1IL** |
| --- | --- | --- | --- |
| EGF [pg mL^-1^] | 8.74 ± 0.83 | 6.62 ± 2.29 ^ns^ | 12.66 ± 3.83 ^ns^ |
| Eotaxin [pg mL^-1^] | 28.16 ± 4.45 | 27.77 ± 4.64 ^ns^ | 36.56 ± 6.22 ^ns^ |
| Fractalkine [pg mL^-1^] | 81.18 ± 1.37 | 113.39 ± 135.63 ^ns^ | 135.63 ± 31.10 ^ns^ |
| G-CSF [pg mL^-1^] | 191.40 ± 78.15 | 165.81 ± 62.94 ^ns^ | 232.55 ± 80.46 ^ns^ |
| GM-CSF [pg mL^-1^] | 542.69 ± 109.10 | 320.18 ± 64.29 ^ns^ | 702.60 ± 288.69 ^ns^ |
| GRO/KC/CINC-1 [pg mL^-1^] | 150.01 ± 18.55 | 116.42 ± 5.26 ^ns^ | 122.43 ± 21.70 ^ns^ |
| IFNγ [pg mL^-1^] | 637.62 ± 293.48 | 662.31 ± 233.61 ^ns^ | 870.89 ± 173.30 ^ns^ |
| IL-1α [pg mL^-1^] | 151.22 ± 78.45 | 196.59 ± 74.55 ^ns^ | 287.91 ± 73.31 ^ns^ |
| IL-1β [pg mL^-1^] | 117.50 ± 40.25 | 173.35 ± 63.44 ^ns^ | 195.24 ± 74.55 ^ns^ |
| IL-2 [pg mL^-1^] | 356.37 ± 47.64 | 583.48 ± 193.02 ^ns^ | 830.87 ± 260.22 ^ns^ |
| IL-4 [pg mL^-1^] | 224.42 ± 102.81 | 242.90 ± 79.99 ^ns^ | 342.21 ± 58.59 ^ns^ |
| IL-5 [pg mL^-1^] | 321.51± 58.20 | 362.36 ± 73.85 ^ns^ | 400.10 ± 47.47 ^ns^ |
| IL-10 [pg mL^-1^] | 112.83 ± 33.81 | 202.83 ± 50.55 ^ns^ | 269.34 ± 64.41 ^ns^ |
| IL-12p70 [pg mL^-1^] | 1,354.07 ± 405.15 | 1,251.99 ± 383.43 ^ns^ | 2,019.01 ± 361.10 ^ns^ |
| IL-13 [pg mL^-1^] | 164.89 ± 32.33 | 162.32 ± 47.36 ^ns^ | 198.380 ± 58.96 ^ns^ |
| IL-17A [pg mL^-1^] | 255.58 ± 73.45 | 221.59 ± 69.46 ^ns^ | 275.62 ± 85.07 ^ns^ |
| IL-18 [pg mL^-1^] | 457.45 ± 76.22 | 931.12 ± 305.20 ^ns^ | 1,141.93 ± 270.67 ^ns^ |
| IP-10 [pg mL^-1^] | 200.08 ± 4.79 | 232.21 ± 16.49 ^ns^ | 209.19 ± 21.87 ^ns^ |
| LIX [pg mL^-1^] | 2,330.66 ± 127.53 | 2,721.24 ± 306.63 ^ns^ | 2,832.87 ± 597.79 ^ns^ |
| MCP-1 [pg mL^-1^] | 2,210.74 ± 166.19 | 2,470.31 ± 720.21 ^ns^ | 2,912.28 ± 262.05 ^ns^ |
| MIP-1α [pg mL^-1^] | 28.62± 2.77 | 42.25± 9.04 ^ns^ | 51.93 ± 10.48 ^ns^ |
| MIP-2 [pg mL^-1^] | 98.20 ± 22.43 | 114.68 ± 15.24 ^ns^ | 135.60 ± 12.95 ^ns^ |
| RANTES [pg mL^-1^] | 2,133.30 ± 414.14 | 1,726.53 ± 170.56 ^ns^ | 1,879.37 ± 459.53 ^ns^ |
| TNFα [pg mL^-1^] | 42.11 ± 8.09 | 52.46 ± 17.42 ^ns^ | 69.73 ± 14.95 ^ns^ |
| VEGF [pg mL^-1^] | 72.98 ± 12.43 | 133.40 ± 43.82 ^ns^ | 202.29 ± 63.07 ^ns^ |

**Table S10.** Serum cytokine/chemokine levels in rats with anorectal fistulas treated with 18NC75 or 18NC75-10P-1IL compared to non-treated rats (Control) at D2 (n=3). Data are mean ± s.e.m.; statistical significance was determined by two-way ANOVA with Tukey’s multiple-comparison test. ns, not significant.

| **Parameter [Unit]** | **Control** | **18NC75** | **18NC75-10P-1IL** |
| --- | --- | --- | --- |
| EGF [pg mL^-1^] | 8.74 ± 0.83 | 11.68 ± 3.45 ^ns^ | 9.28 ± 1.76 ^ns^ |
| Eotaxin [pg mL^-1^] | 28.16 ± 4.45 | 38.30 ± 6.16 ^ns^ | 36.29 ± 5.77 ^ns^ |
| Fractalkine [pg mL^-1^] | 81.18 ± 1.37 | 117.50 ± 4.52 ^ns^ | 116.42 ± 29.00 ^ns^ |
| G-CSF [pg mL^-1^] | 191.40 ± 78.15 | 573.63 ± 133.57 ^ns^ | 673.29 ± 187.37 ^ns^ |
| GM-CSF [pg mL^-1^] | 542.69 ± 109.10 | 573.63 ± 133.57 ^ns^ | 673.29 ± 187.37 ^ns^ |
| GRO/KC/CINC-1 [pg mL^-1^] | 150.01 ± 18.55 | 151.95 ± 31.76 ^ns^ | 133.92 ± 8.74 ^ns^ |
| IFNγ [pg mL^-1^] | 637.62 ± 293.48 | 831.98 ± 195.38 ^ns^ | 910.15 ± 180.66 ^ns^ |
| IL-1α [pg mL^-1^] | 151.22 ± 78.45 | 272.06 ± 68.07 ^ns^ | 271.42 ± 56.69 ^ns^ |
| IL-1β [pg mL^-1^] | 117.50 ± 40.25 | 172.46 ± 70.40 ^ns^ | 170.42 ± 73.52 ^ns^ |
| IL-2 [pg mL^-1^] | 356.37 ± 47.64 | 767.95 ± 270.81 ^ns^ | 664.99 ± 200.30 ^ns^ |
| IL-4 [pg mL^-1^] | 224.42 ± 102.81 | 357.08 ± 67.98 ^ns^ | 292.79 ± 71.32 ^ns^ |
| IL-5 [pg mL^-1^] | 321.51± 58.20 | 373.16 ± 52.99 ^ns^ | 378.13 ± 52.77 ^ns^ |
| IL-10 [pg mL^-1^] | 112.83 ± 33.81 | 252.29 ± 84.68 ^ns^ | 249.18 ± 69.18 ^ns^ |
| IL-12p70 [pg mL^-1^] | 1,354.07 ± 405.15 | 1,631.95 ± 381.83 ^ns^ | 1,543.20 ± 353.30 ^ns^ |
| IL-13 [pg mL^-1^] | 164.89 ± 32.33 | 182.37 ± 21.60 ^ns^ | 174.71 ± 26.96 ^ns^ |
| IL-17A [pg mL^-1^] | 255.58 ± 73.45 | 287.41 ± 118.26 ^ns^ | 250.43 ± 54.92 ^ns^ |
| IL-18 [pg mL^-1^] | 457.45 ± 76.22 | 865.18 ± 262.56 ^ns^ | 826.48 ± 190.93 ^ns^ |
| IP-10 [pg mL^-1^] | 200.08 ± 4.79 | 308.24 ± 56.29 ^ns^ | 201.63 ± 12.91 ^ns^ |
| LIX [pg mL^-1^] | 2,330.66 ± 127.53 | 2,025.85 ± 332.77 ^ns^ | 2,102.72 ± 436.78 ^ns^ |
| MCP-1 [pg mL^-1^] | 2,210.74 ± 166.19 | 2,733.09 ± 344.39 ^ns^ | 2,980.25 ± 435.68 ^ns^ |
| MIP-1α [pg mL^-1^] | 28.62± 2.77 | 51.05 ± 18.47 ^ns^ | 48.50 ± 10.30 ^ns^ |
| MIP-2 [pg mL^-1^] | 98.20 ± 22.43 | 136.12 ± 19.97 ^ns^ | 123.58 ± 19.17 ^ns^ |
| RANTES [pg mL^-1^] | 2,133.30 ± 414.14 | 1,899.98 ± 265.55 ^ns^ | 1,268.84 ± 347.44 ^ns^ |
| TNFα [pg mL^-1^] | 42.11 ± 8.09 | 63.47 ± 12.61 ^ns^ | 66.26 ± 15.55 ^ns^ |
| VEGF [pg mL^-1^] | 72.98 ± 12.43 | 158.57 ± 45.06 ^ns^ | 129.51 ± 47.62 ^ns^ |

**Table S11.** Serum cytokine/chemokine levels in rats with anorectal fistulas treated with 18NC75 or 18NC75-10P-1IL compared to non-treated rats (Control) at D7 (n=3). Data are mean ± s.e.m.; statistical significance was determined by two-way ANOVA with Tukey’s multiple-comparison test. ns, not significant.

| **Parameter [Unit]** | **Control** | **18NC75** | **18NC75-10P-1IL** |
| --- | --- | --- | --- |
| EGF [pg mL^-1^] | 8.74 ± 0.83 | 9.77 ± 3.54 ^ns^ | 11.86 ± 3.87 ^ns^ |
| Eotaxin [pg mL^-1^] | 28.16 ± 4.45 | 34.37 ± 7.52 ^ns^ | 37.52 ± 4.50 ^ns^ |
| Fractalkine [pg mL^-1^] | 81.18 ± 1.37 | 107.63 ± 24.07 ^ns^ | 108.60 ± 20.16 ^ns^ |
| G-CSF [pg mL^-1^] | 191.40 ± 78.15 | 295.49 ± 152.59 ^ns^ | 370.16 ± 151.73 ^ns^ |
| GM-CSF [pg mL^-1^] | 542.69 ± 109.10 | 505.67 ± 261.19 ^ns^ | 947.56 ± 201.07 ^ns^ |
| GRO/KC/CINC-1 [pg mL^-1^] | 150.01 ± 18.55 | 122.44 ± 19.83 ^ns^ | 176.80 ± 23.30 ^ns^ |
| IFNγ [pg mL^-1^] | 637.62 ± 293.48 | 674.08 ± 309.64 ^ns^ | 1,065.62 ± 347.87 ^ns^ |
| IL-1α [pg mL^-1^] | 151.22 ± 78.45 | 218.67 ± 85.14 ^ns^ | 323.31 ± 106.47 ^ns^ |
| IL-1β [pg mL^-1^] | 117.50 ± 40.25 | 147.73 ± 71.72 ^ns^ | 145.26 ± 40.87 ^ns^ |
| IL-2 [pg mL^-1^] | 356.37 ± 47.64 | 476.92 ± 132.02 ^ns^ | 596.69 ± 196.03 ^ns^ |
| IL-4 [pg mL^-1^] | 224.42 ± 102.81 | 293.51 ± 90.78 ^ns^ | 318.21 ± 83.05 ^ns^ |
| IL-5 [pg mL^-1^] | 321.51± 58.20 | 335.95 ± 66.86 ^ns^ | 381.21 ± 83.05 ^ns^ |
| IL-10 [pg mL^-1^] | 112.83 ± 33.81 | 184.82 ± 90.47 ^ns^ | 246.31 ± 73.18 ^ns^ |
| IL-12p70 [pg mL^-1^] | 1,354.07 ± 405.15 | 1,292.12 ± 408.08 ^ns^ | 1,599.66 ± 453.54 ^ns^ |
| IL-13 [pg mL^-1^] | 164.89 ± 32.33 | 163.16 ± 48.43 ^ns^ | 221.76 ± 52.18 ^ns^ |
| IL-17A [pg mL^-1^] | 255.58 ± 73.45 | 272.51 ± 92.68 ^ns^ | 334.56 ± 69.26 ^ns^ |
| IL-18 [pg mL^-1^] | 457.45 ± 76.22 | 692.21± 183.93 ^ns^ | 989.79 ± 313.37 ^ns^ |
| IP-10 [pg mL^-1^] | 200.08 ± 4.79 | 182.85 ± 26.23 ^ns^ | 172.90 ± 32.17 ^ns^ |
| LIX [pg mL^-1^] | 2,330.66 ± 127.53 | 2,618.58 ± 323.51 ^ns^ | 1,540.22 ± 258.70 ^ns^ |
| MCP-1 [pg mL^-1^] | 2,210.74 ± 166.19 | 2,375.41 ± 347.84 ^ns^ | 2,853.80 ± 410.66 ^ns^ |
| MIP-1α [pg mL^-1^] | 28.62± 2.77 | 42.39 ± 10.51 ^ns^ | 46.65 ± 11.78 ^ns^ |
| MIP-2 [pg mL^-1^] | 98.20 ± 22.43 | 112.10 ± 24.47 ^ns^ | 158.39 ± 26.33 ^ns^ |
| RANTES [pg mL^-1^] | 2,133.30 ± 414.14 | 1,744.36 ± 77.51 ^ns^ | 1,152.84 ± 271.65 ^ns^ |
| TNFα [pg mL^-1^] | 42.11 ± 8.09 | 59.91 ± 18.41 ^ns^ | 67.98 ± 20.91 ^ns^ |
| VEGF [pg mL^-1^] | 72.98 ± 12.43 | 96.99 ± 32.43 ^ns^ | 94.12 ± 49.99 ^ns^ |
